# Structure‐Activity Relationship of Phenylpyrazolones against *Trypanosoma cruzi*

**DOI:** 10.1002/cmdc.202000136

**Published:** 2020-04-27

**Authors:** Maarten Sijm, Geert Jan Sterk, Guy Caljon, Louis Maes, Iwan J. P. de Esch, Rob Leurs

**Affiliations:** ^1^ Division of Medicinal Chemistry, Faculty of Sciences Amsterdam Institute for Molecules, Medicines and Systems (AIMMS) Vrije Universiteit Amsterdam De Boelelaan 1108 1081 HZ Amsterdam The Netherlands; ^2^ Laboratory for Microbiology, Parasitology and Hygiene (LMPH) University of Antwerp Universiteitsplein 1 2610 Antwerpen Belgium

**Keywords:** Benznidazole, neglected parasitic diseases, phenylpyrazolones, structure-activity relationships, *Trypanosoma cruzi*

## Abstract

Chagas disease is a neglected parasitic disease caused by the parasitic protozoan *Trypanosoma cruzi* and currently affects around 8 million people. Previously, 2‐isopropyl‐5‐(4‐methoxy‐3‐(pyridin‐3‐yl)phenyl)‐4,4‐dimethyl‐2,4‐dihydro‐3*H*‐pyrazol‐3‐one (NPD‐0227) was discovered to be a sub‐micromolar inhibitor (pIC_50_=6.4) of *T. cruzi*. So far, SAR investigations of this scaffold have focused on the alkoxy substituent, the pyrazolone nitrogen substituent and the aromatic substituent of the core phenylpyrazolone. In this study, modifications of the phenyldihydropyrazolone scaffold are described. Variations were introduced by installing different substituents on the phenyl core, modifying the geminal dimethyl and installing various bio‐isosteres of the dihydropyrazolone group. The anti *T. cruzi* activity of NPD‐0227 could not be surpassed as the most potent compounds show pIC_50_ values of around 6.3. However, valuable additional SAR data for this interesting scaffold was obtained, and the data suggest that a scaffold hop is feasible as the pyrazolone moiety can be replaced by a oxazole or oxadiazole with minimal loss of activity.

## Graphical abstract

**Testing the scaffold**: A series of phenylpyrazolones has been investigated to find compounds that are active against *T. cruzi*. Although none could match the activity of NPD‐0227 (pIC_50_=6.4), the SAR study turned up valuable data for hit‐to‐lead optimization and improving the selectivity index, such as the possibility of replacing the pyrazolone moiety by an oxazole or oxadiazole.

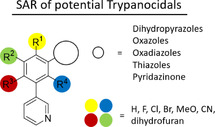

## Introduction

The protozoan parasite *Trypanosoma cruzi* is the causative agent of Chagas disease. This parasite is transmitted by the triatomine bug vector that used to be endemic only in Latin America, but is now slowly moving towards North America as well.^1^ It is estimated that currently around 8 million people are infected and many more are at risk of being infected.^2^ Upon infection, the disease first enters into an acute phase in which symptoms are generally mild, fever‐like, uncharacteristic or even absent.^3^ As a result, Chagas disease is often not diagnosed in this stage and proceeds untreated towards a chronic phase.^4^ Although the initial symptoms will disappear after a few weeks, the parasite will persist and evolve to an indeterminate symptomatic chronic phase.^5^ This ultimately develops into progressive chronic cardiomyopathy in 30 % of the patients while another 10 % develop neurological, digestive or mixed clinical symptoms. Although the remaining 60 % do not develop any symptoms, they remain infective if untreated and therefor remain an infection risk.^6^

**Figure 1:**
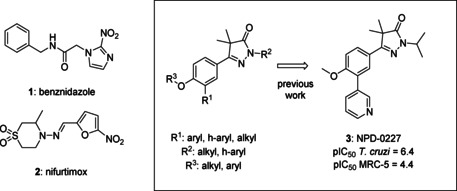
Current drugs for Chagas disease: benznidazole (**1**) and nifurtimox (**2**). NPD‐0227 (**3**) was obtained from earlier hit optimization against *T. cruzi*.^10^

While currently only two drugs are on the market, these are far from optimal as they have long treatment regimes, cause adverse drug effects and have limited efficacy during the chronic phase.^7^ Benznidazole (**1**, Figure 1) and nifurtimox (**2**) are nitro‐heteroaromatic drugs that were developed in the late 1960s. Their efficacy in the acute phase is widely accepted, however, their effectiveness during the chronic phase is still under debate.^8^ In addition, they are known to cause adverse drug effects such as weight loss, depression and amnesia.7c, 9

With these limitations of the current drugs, it is clear that the need for novel chemotherapies is high. The drug discovery pipeline of Chagas disease has a few potential clinical candidates, mostly from private‐public partnerships originating from the last decade. Meanwhile, drug resistance has been reported for both benznidazole (**1**, Figure 1) and nifurtimox (**2**) in *in vitro* strains.^11^ Within the PDE4NPD (phosphodiesterase inhibitors for neglected parasitic disease) consortium, an European Union‐funded public‐private partnership to target several neglected tropical diseases, Chagas disease was one of the focus points. We previously reported the discovery of NPD‐0227 (**3**, Figure 1), a sub‐micromolar inhibitor of *T. cruzi* and modifications of this hit have been described with focus on substituents of the aromatic substituent (R^1^), pyrazolone nitrogen (R^2^) and the alkoxy substituent (R^3^).^10^ In this previous work, modifications on R^2^ and R^3^ did not result in increased activities. To further investigate the structure‐activity‐relationships of this scaffold, the present work focused on the effect of modifications of the core phenyl ring replacing the gem‐dimethyl moiety of the dihydropyrazolone and replacement of the dihydropyrazolone ring with various heterocyclic and aromatic moieties.

## Chemistry

**Scheme 1:**
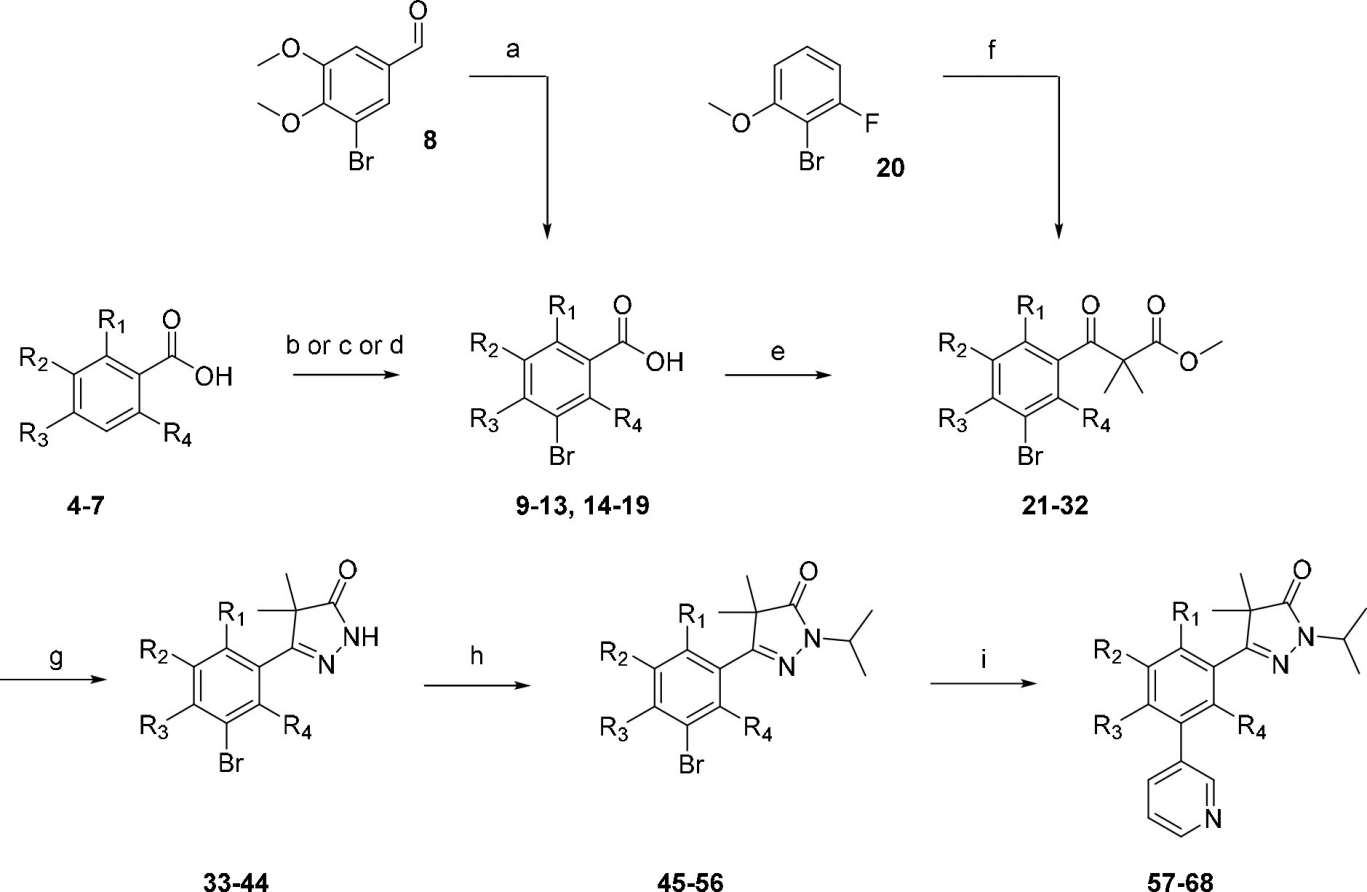
Preparation of pyrazolones **57**–**68** (Table 1) with modifications on the central phenyl ring. a) KMnO_4_, KPO_4_H_2_, *t*BuOH, RT, 16 h, 79 %; b) Br_2_, dioxane, RT, 16 h‐9d, 68–95 %; c) Br_2_, AcOH, 60 °C, 4 h, 75 %; d) Br_2_, Fe, CHCl_3_, RT, 8 h, 35 %; e) i: (COCl)_2_, DMF, CH_2_Cl_2_, RT, 4 h, ii: LDA, methylisobutyrate, THF, −78 °C to RT, 2 h; f) i: 3‐ethoxy‐2,2‐dimethyl‐3‐oxopropanoic acid, (COCl)_2_, DMF, CH_2_Cl_2_, ii: AlCl_3_, CH_2_Cl_2_; g) N_2_H_4_, EtOH, RT, 16 h, 6–91 %; h) 2‐bromopropane, NaH, DMF, RT, 16 h, 29–85 %; i) 3‐pyridinyl‐B(OH)_2_, Pd(dppf)Cl_2_ ⋅ CH_2_Cl_2_, Na_2_CO_3_, DME/H_2_O, 120 °C, 1 h, 6–88 %.

To introduce different substituents on the phenyl moiety of the phenyldihydropyrazole scaffold, the previously reported methodology by Sijm et al. was used, starting with the desired benzoic acids (**9**–**19**, Scheme 1).^10^ Several benzoic acids had to be prepared from their precursors. Bromination of the non‐halogenated benzoic acids **4**–**7** resulted in the desired brominated analogues (**10**–**13**). During the bromination which led to benzoic acid **13** (5‐bromo‐4‐methoxy‐2‐methylbenzoic acid), two regioisomers were formed in a 2 : 1 ratio (3‐Br/5‐Br). Quenching of the mixture with a sodium thiosulfate solution (∼1 M) in water gave a biphasic mixture with substantial precipitation. Filtering off the solids resulted in collection of a single (3‐Br) isomer (**13**). Despite substantial recrystallization efforts the 5‐Br‐isomer could not be isolated with sufficient purity. Subsequent oxidation of aldehyde **8** with potassium permanganate yielded benzoic acid **9**, while the remaining benzoic acids (**14**–**19**) were bought from commercial suppliers. The obtained benzoic acids (**9**–**19**) were converted to the corresponding acid chlorides with oxalyl chloride, which was followed by addition of the lithium enolate of methyl isobutyrate, yielding keto esters **21**–**28** and **30**–**32**. Exception was the 3‐bromo‐2‐fluoro‐3‐methoxy keto‐ester (**29**) which was obtained by the Friedel‐Craft acylation of anisole **20**. The obtained keto‐esters (**21**–**32**, Scheme 1) were subsequently condensed with hydrazine to give the core phenyl‐dihydropyrazolone scaffold with various substituents on the phenyl ring (**33**–**44**).

**Scheme 2:**
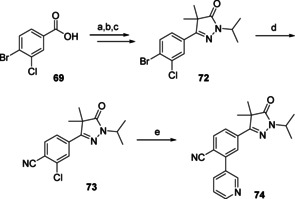
Preparation of pyrazolone **74** (Table 1) with modification on the central phenyl ring. a) i: (COCl)_2_, DMF, CH_2_Cl_2_, RT, 4 h; ii: LDA, methylisobutyrate, THF, −78 °C to RT, 2 h; b) N_2_H_4_, EtOH, RT, 16 h, 24 % over two steps; c) 2‐bromopropane, NaH, DMF, RT, 16 h, 75 %; d) CuCN, DMF, 150 °C, 18 h, 59 %; e) 3‐pyridinyl‐B(OH)_2_, Pd(dppf)Cl_2_ ⋅ CH_2_Cl_2_, Na_2_CO_3_, DME/H_2_O, 120 °C, 1 h, 61 %.

N‐Alkylation of the diydropyrazolones (**33**–**44**, Scheme 1) was done using sodium hydride and isopropylbromide, installing the desired isopropyl moiety (**45**–**56**). The final step was a Suzuki cross‐coupling to install a 3‐pyridinyl ring on the 3‐position yielding twelve analogues (**57**–**68**) of NPD‐0227 (**3**, Figure 1) with variations on the central phenyl ring. The 4‐cyanophenyl (**74**) was prepared via a similar route: starting from 4‐bromo‐3‐chlorobenzoic acid (**69**, scheme 2). This benzoic acid was transofrmed to the corresponding isopropyl‐pyrazolone (**72**) in three steps. The key step was the conversion of the bromine towards the cyano moiety by using CuCN in DMF, after which this intermediate (**73**) was used in a Suzuki cross‐couping to install the final 3‐pyridinyl moiety (**74**).

**Scheme 3:**
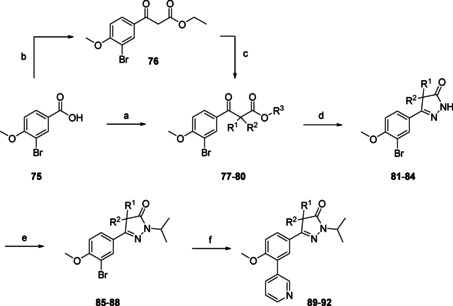
Introduction of variations on the gem‐dimethyl position of NPD‐0227 (**3**), resulting in **89**–**92** (Table 2). a) i: (COCl)_2_, DMF, CH_2_Cl_2_, RT, 4 h; ii: LDA, methylisobutyrate, THF, −78 °C to RT, 2 h; b) ethyl potassium malonate, TEA, CDI, MgCl_2_, ACN, THF, 44 %; c) K_2_CO_3_, 1,4‐dibromobutane, DMSO, RT, 51 %; d) N_2_H_4_, EtOH, RT, 16 h, 20–65 % over two steps; e) 2‐bromopropane, NaH, DMF, RT, 16 h, 74–96 %; f) 3‐pyridinyl‐B(OH)_2_, Pd(dppf)Cl_2_ ⋅ CH_2_Cl_2_, Na_2_CO_3_, DME/H_2_O, 120 °C, 1 h, 22–60 %.

Modifications of the gem‐dimethyl moiety present in NPD‐0227 (**3**) were installed in an early stage of the synthesis route (Scheme 3). Similar conditions were used as in Schemes 1 and 2; benzoic acid **75** was transformed to the corresponding acid chloride, after which the desired lithium enolates were added to yield the cyclopentene (**77**), methylpiperidine (**79**) and tetrahydropyran analogues (**80**). Exception was the cyclopentyl substituted keto‐ester (**78**), which was prepared by a one‐pot reaction in which carboxylic acid (**75**) was converted to the corresponding imidazolide, followed by addition of ethyl potassium malonate and subsequent decarboxylation. This unsubstituted keto‐ester (**76**) was then dialkylated with 1,4‐dibromobutane to yield the desired cyclopentyl ring. These four keto‐esters (**77**–**80**) were then exposed to the same sequence as in Scheme 1 to yield *spiro* analogues **89**–**92**.

**Scheme 4:**
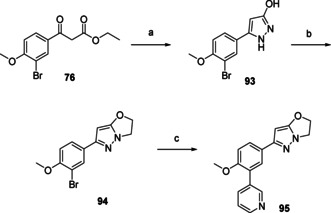
Preparation of dihydropyrazolo‐oxazole **95** (Table 3). a) N_2_H_4_, EtOAc, RT, o/n, 67 %; b) K_2_CO_3_, 1,2‐dibromoethane, DMF, 80 °C, 2 h, 37 %; c) 3‐pyridinyl‐B(OH)_2_, Pd(dppf)Cl_2_ ⋅ CH_2_Cl_2_, Na_2_CO_3_, DME/H_2_O, 120 °C, 1 h.

**Scheme 5:**
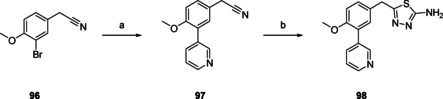
Preparation of thiadiazole **98** (Table 3). a) 3‐pyridinyl‐B(OH)_2_, Pd(dppf)Cl_2_ ⋅ CH_2_Cl_2_, Na_2_CO_3_, DME/H_2_O, 120 °C, 1 h, 91 %; b) hydrazinecarbothiamide, TFA, 60 °C, 6 h, 71 %.

**Scheme 6:**
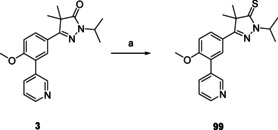
Preparation of the thione analogue of previous optimized hit NPD‐0227, **99** (Table 3). a) Lawessons reagent, THF, reflux, 32 h, 76 %.

**Scheme 7:**
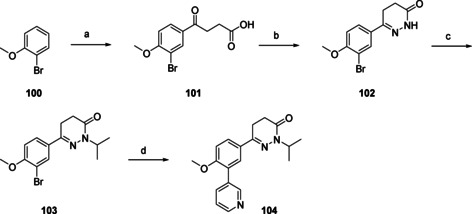
Preparation of the dihydropyridazinone analogue **104** (Table 3). a) AlCl_3_, succinic anhydride, PhNO_2_, 60 °C, 4 h, 42 %; b) N_2_H_4_ ⋅ H_2_O, EtOH, reflux, 1 h, 89 %; c) 2‐bromopropane, NaH, DMF, 50 °C, 3 h, 87 %; d) 3‐pyridinyl‐B(OH)_2_, Pd(dppf)Cl_2_ ⋅ CH_2_Cl_2_, Na_2_CO_3_, DME/H_2_O, 120 °C, 1 h, 63 %.

**Scheme 8:**
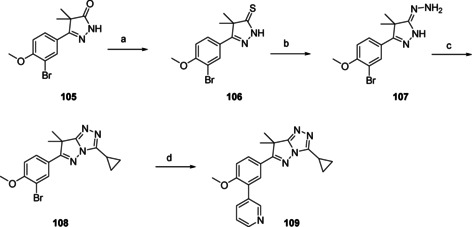
Preparation of cyclopropylpyrolotriazole **109** (Table 3). a) Lawessons reagent, toluene, reflux, o/n, 69 %; b) N_2_H_4_ ⋅ H_2_O, THF, RT→70 °C, 5 h, 82 %; c) cyclopropanecarbonylchloride, pyridine, 70 °C, 2 h, then DMF, 150 °C, 2 h, 47 %; d) 3‐pyridinyl‐B(OH)_2_, Pd(dppf)Cl_2_ ⋅ CH_2_Cl_2_, Na_2_CO_3_, DME/H_2_O, 120 °C, 1 h, 30 %.

**Scheme 9:**
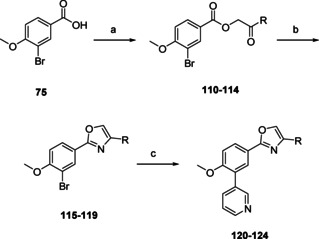
Preparation of oxazoles **120**–**124** (Table 4). a) BrCH_2_COR, NaH, DMF, RT, 30 min, 83–94 %; b) NH_4_OAc, AcOH, 170 °C, 6 h, 11–36 %; c) 3‐pyridinyl‐B(OH)_2_, Pd(dppf)Cl_2_ ⋅ CH_2_Cl_2_, Na_2_CO_3_, DME/H_2_O, 120 °C, 1 h, 36–74 %.

**Scheme 10:**
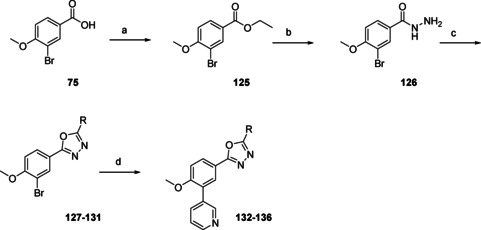
Preparations of oxadiazoles **132**–**136** (Table 4). a) H_2_SO_4_, EtOH, reflux, 6 h, 97 %; b) N_2_H_4_ ⋅ H_2_O, EtOH, reflux, 16 h, 53 %; c) R‐COOH, POCl_3_, reflux, 1 h, 42–80 %; d) 3‐pyridinyl‐B(OH)_2_, Pd(dppf)Cl_2_ ⋅ CH_2_Cl_2_, Na_2_CO_3_, DME/H_2_O, 120 °C, 1 h, 32–65 %.

**Scheme 11:**
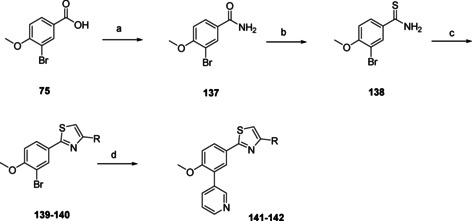
Synthesis of thiazoles **141** and **142** (Table 4). a) (COCl)_2_, DMF, CH_2_Cl_2_, RT, 18 h; b) 30 % NH_4_OH in H_2_O, CH_2_Cl_2_, RT, 5 min, 75 % over two steps; c) Lawessons reagent, toluene, reflux, 18 h, 13 %; d) BrCH_2_COR, propan‐2‐ol, RT, 2 h; e) 3‐pyridinyl‐B(OH)_2_, Pd(dppf)Cl_2_ ⋅ CH_2_Cl_2_, Na_2_CO_3_, DME/H_2_O, 120 °C, 1 h, 35–44 % over two steps.

Various bio‐isosteres of the dihydropyrazolone moiety were installed according to different synthetic routes (Schemes 4 and 5–11, below). Dihydropyrazolo‐oxazole **95** (Scheme 3) was prepared in three steps from keto‐ester **76**. First this molecule was ring‐closed with hydrazine resulting in pyrazole **93**, followed by an alkylation with 1,2‐dibromoethane, yielding the pyrazolo‐oxazole moiety (**94**). Final step was a Suzuki reaction using previously shown conditions to yield the desired 3‐pyridine substituted pyrazolo‐oxazole **95**.

Thiadiazole **98** (Scheme 5) was prepared in two steps from 2‐(3‐bromo‐4‐methoxyphenyl)acetonitrile (**96**). First step was a Suzuki cross coupling to yield 3‐pyridinyl intermediate **97**. Subsequent ring closure with hydrazinecarbothioamide yielded the final thiadiazole **98**.

Pyrazole‐3‐thione **99** (Scheme 6), the sulfur bio‐isostere of NPD‐0227 (**3**) was prepared in one step, from NPD‐0227 (**3**) by using Lawesson's reagent in THF.

The dihydropyridazinone moiety (Scheme 7) has some similarity to the dihydropyrazolone moiety of NPD‐0227 (**3**), although this ring contains one additional carbon. Installation of this moiety started with a Friedel‐Craft acylation of 2‐bromoanisole **100**, resulting in β‐keto‐acid **101**. Ring closure of this molecule with hydrazine gave the dihydropyridazinone ring (**102**) which was subsequently alkylated using sodium hydride to give the *N*‐isopropyl derivative (**103**). Similar to previously described synthetic routes, last step of installing the 3‐pyridinyl moiety was done using Suzuki conditions as described earlier, resulting in dihydropyridazinone **104**.

Pyrolotriazole **109** (Scheme 8) is a bicyclic heterocycle with similarity to the original dihydropyrazolone. Preparation starts from the previously reported dihydropyrazolone **105** which was refluxed with Lawessons reagent to yield pyrazol‐3‐thione **106**.^10^ Addition of hydrazine to this building block yielded hydrazineylidine **107**, from which the second heterocyclic ring was formed by adding cyclopropanecarbonyl chloride, resulting to the pyrolotriazole moiety (**108**). A final Suzuki cross‐coupling yielded pyrolotriazole **109**. Attempts to install an isopropyl, cyclopentyl or *n*‐propyl did not succeed as the ring‐closure step proved problematic.

Oxazoles **120**–**124** (Scheme 9) were prepared from benzoic acid **75**; first step of this route was a sodium hydride promoted nucleophilic attack of the benzoic acid on selected bromoketones, yielding keto‐esters **110**–**114**. Subsequent ring closure with ammonium acetate yielded mixtures of respective imidazoles and oxazoles (**115**–**119**), which were relatively easily separated by column chromatography. Although attempts were made to isolate the imidazoles, these could not be obtained in sufficient purity. The subsequent Suzuki cross coupling yielded the desired 3‐pyridine substituted phenyloxazoles **120**–**124**.

Oxadiazoles **132**–**136** (Scheme 10) were prepared from benzoic acid **75**, which was transformed to the ethyl ester by refluxing in EtOH in the presence of H_2_SO_4_. Addition of hydrazine to ester **125**, yielded hydrazide **126**, which was ring closed with the desired acid chlorides in the presence of POCl_3_ to yield oxadiazoles **127**–**131**. The final step was Suzuki cross‐coupling to yield 3‐pyridinyl substituted phenyloxadiazoles **132**–**136**.

The final heterocyclic replacement investigated the thiazole ring (Scheme 11). The starting material was benzoic acid **75**, which was chlorinated using oxalyl chloride, followed by a quench of ammonium hydroxide to yield benzamide **137**. The benzamide was converted to the corresponding thioamide **138** using Lawessons reagent. This intermediate (**138**) could be used to form the desired thiazoles upon addition of bromoketones, yielding analogues **139** and **140**. To finalize the molecules a Suzuki cross‐coupling was used to install the 3‐pyridinyl moiety, resulting in compounds **141** and **142**.

## Results and Discussion

In this work, the SAR around NPD‐0227 (**3**) has been further investigated with modifications on the central phenyl ring, the gem‐dimethyl moiety and the dihydropyrazolone headgroup. In Table 1, the screening results are shown of various substituents on the core phenyl group. Attempts to move the 4‐methoxy of NPD‐0227 to the 5‐position (**57**) leads to a decrease in activity with a pIC_50_ value of 5.1, while introducing a methoxy substituent on both the 4‐ and 5‐ position (**58**), shows an even larger decrease in activity (pIC_50_=4.3).

**Table 1:** Phenotypic activity of core‐phenyl modifications against intracellular amastigotes of *T. cruzi* (Tulahuen strain) and MRC‐5 cells.

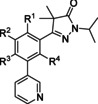							
Cmpd	R^1^	R^2^	R^3^	R^4^	pIC_50_^[a]^		SI^[b]^
					*T. cruzi*	MRC‐5	
**3**	H	H	MeO	H	6.4	4.4	100
**57**	H	MeO	H	H	5.1	4.5	4
**58**	H	MeO	MeO	H	4.3	<4.2	>1
**59**	H	−CH_2_CH_2_O−		H	5.8	4.6	16
**60**	H	Br	MeO	H	5.1	4.6	3
**61**	H	H	H	H	5.5	4.2	20
**62**	H	H	F	H	5.2	<4.2	>10
**63**	H	H	Cl	H	5.5	4.8	5
**64**	H	H	Me	H	5.6	<4.2	>25
**65**	H	H	MeO	F	5.6	<4.2	>25
**66**	F	H	MeO	H	5.3	<4.2	>13
**67**	H	Me	MeO	H	4.9	<4.2	>5
**68**	Me	H	MeO	H	4.5	<4.2	>2
**74**	H	H	CN	H	4.3	<4.2	>1

[a] All reported values are within a standard deviation of ±0.2 and the result of at least *n*=2. [b] The selectivity index is calculated by dividing the cytotoxicity (IC_50_) by the *T. cruzi* activity (IC_50_).

Constraining the methoxy‐substituent in a heterocycle resulting in dihydrobenzofuran **59** only resulted in a small decrease (pIC_50_=5.8) in activity compared to NPD‐0227. Installing a bromine on the phenyl ring next to the 4‐methoxy substituent (**60**) resulted in a decrease in activity with a pIC_50_ value of 5.1. Removal of the 4‐methoxy led to analogue **61**, which resulted in a tenfold drop in potency (pIC_50_=5.5), showing that a methoxy substituent on this position is beneficial. Installation of a fluorine (**62**), chlorine (**63**), or methyl (**64**) group instead of the original 4‐methoxy substituent resulted in compounds with similar pIC_50_ values around 5.5 as the unsubstituted analogue **61**. Introduction of a cyano moiety (**74**) instead of a methoxy resulted in an even further drop in activity with a pIC_50_ value of 4.3.

As the 4‐methoxy substituted analogue (**3**) performed the best thus far, additional substituents along with the methoxy were investigated. Both the 2‐fluoro‐4‐methoxy (**65**) and the 6‐fluoro‐4‐methoxy (**66**) showed an approximately tenfold decrease compared to NPD‐0227 (**3**); the 4‐methoxy‐5‐methyl (**67**) and 4‐methoxy‐6‐methyl (**68**) showed an even larger decrease with pIC_50_ values of 4.9 and 4.5, respectively. Selectivity index between *T. cruzi* activity and MRC‐5 cytotoxicity are lower then seen with NPD‐0227, with the best compounds showing a SI of >25‐fold. Exact SIs are not known as the lowest concentration measured for cytotoxicity is 62 μM

Four compounds were prepared with variations of the *gem*‐dimethyl moiety of NPD‐0227 (**3**); installing a cyclopentyl (**89**, Table 2) and a cyclopentene (**90**) both resulted in a more than tenfold loss in activity compared to NPD‐0227 (**3**). Introduction of the more polar methylpiperidine (**91**) and a tetrahydropyran (**92**) resulted in an even further decrease in potency with both compounds having a pIC_50_ value of 4.5. Selectivity index between *T. cruzi* activity and MRC‐5 cytotoxicity are poor for these series, with the best compounds (**89**, **90**) showing fivefold selectivity.

**Table 2:** Phenotypic activity of analogues with *gem*‐dimethyl modifications against intracellular amastigotes of *T. cruzi* (Tulahuen strain) and MRC‐5 cells.

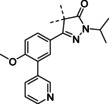				
Cmpd	R	pIC_50_^[a]^		SI^[b]^
		*T. cruzi*	MRC‐5	
**89**	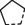	5.2	4.5	5
**90**	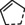	5.1	4.4	5
**91**	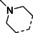	4.5	4.3	2
**92**	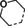	4.5	4.5	1

[a] All reported values are within a standard deviation of ±0.2 and the result of at least *n*=2. [b] The selectivity index is calculated by dividing the cytotoxicity (IC_50_) by the *T. cruzi* activity (IC_50_).

Diydropyrazolo‐oxazole **95** (Table 3), in which the dihydropyrazolone oxygen is constrained in a second five‐membered ring shows a loss in activity with a pIC_50_ value of 4.5. An even larger loss in activity is observed with aminothiodiazole **98**, which is inactive (pIC_50_<4.2). Replacing the oxygen of the dihydropyrazolone with a sulfur atom, leads to dihydropyrazolethione **99**, which had similar activity (pIC_50_=6.3) as NPD‐0227 (**3**, pIC_50_=6.4), although a small increase in toxicity against human MRC‐5 cells (pIC_50_=4.8) is observed, which is a 32‐fold selectivity. Adding an extra carbon to the ring, resulting in pyridazinone **104** leads to a pIC_50_ value of 5.0. Also pyrazolotriazole **109**, which has a bicyclic system with quite some similarity to the pyrazolone ring of NPD‐0227 (**3**), shows a decreased activity (pIC_50=_4.8).

**Table 3:** Phenotypic activity of dihydropyrazolone bio‐isosteres against intracellular amastigotes of *T. cruzi* (Tulahuen strain) and MRC‐5 cells.

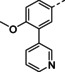				
Cmpd	Headgroup	pIC_50_^[a]^		SI^[b]^
		*T. cruzi*	MRC‐5	
**95**	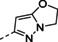	4.5	4.3	2
**98**	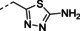	<4.2	<4.2	‐
**99**	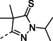	6.3	4.8	32
**104**	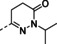	5.0	4.6	3
**109**	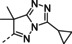	4.8	<4.2	>4

[a] All reported values are within a standard deviation of ±0.2 and the result of at least *n*=2. [b] The selectivity index is calculated by dividing the cytotoxicity (IC_50_) by the *T. cruzi* activity (IC_50_).

To investigate if the dihydropyrazolone moiety could be replaced, three different aromatic heterocycles were installed on this position while still being able to address the same region as the isopropyl moiety of NPD‐0227 (**3**). This resulted in a series of five membered heterocycles: oxazoles (**120**‐**124**, Table 4), oxadiazoles (**132**–**136**) and thiazoles (**141**–**142**). The synthesized oxazoles (**120**–**124**) all showed fairly similar activities with pIC_50_ values around 6.0, although large differences can be seen in toxicity towards MRC‐5 cells. While the *tert*‐butyl (**120**), phenyl (**121**) and 3‐fluorophenyl (**122**) substituted oxazole and phenyl substituted methyloxazole (**124**) all have MRC‐5 toxicities above 5.2, the 4‐fluorophenyl (**123**) substituted oxazole shows no MRC‐5 toxicity at 62 μM. With its low activity and high selectivity (SI>100‐fold) over MRC‐5 cells, 4‐fluorophenyloxazole **123** is the most promising of this series.

**Table 4:** Phenotypic activity of analogues with gem‐dimethyl modifications against intracellular amastigotes of *T. cruzi* (Tulahuen strain) and MRC‐5 cells.

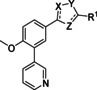							
Cmpd	X	Y	Z	R^1^	pIC_50_^[a]^		SI^[b]^
					*T. cruzi*	MRC‐5	
**120**	O	CH	N	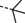	5.7	5.2	3
**121**	O	CH	N	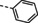	6.0	5.5	3
**122**	O	CH	N	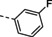	5.9	6.1	1
**123**	O	CH	N	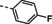	6.2	<4.2	>100
**124**	O	CCH_3_	N	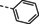	5.7	5.3	3
**132**	N	N	O	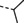	4.7	<4.2	>3
**133**	N	N	O	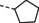	5.0	<4.2	>6
**134**	N	N	O	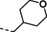	4.6	<4.2	>3
**135**	N	N	O	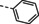	5.8	<4.2	>40
**136**	N	N	O	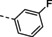	5.7	<4.2	>32
**141**	S	CH	N	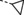	5.6	5.1	3
**142**	S	CH	N	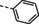	5.7	5.6	1

[a] All reported values are within a standard deviation of ±0.2 and the result of at least *n*=2. [b] The selectivity index is calculated by dividing the cytotoxicity (IC_50_) by the *T. cruzi* activity (IC_50_).

Addition of an extra nitrogen to the heteroaromatic ring lead to oxadiazoles **132**–**136** which showed no toxicity towards MRC‐5 cells at the lowest concentration screened, as all compounds reported pIC_50_ values below 4.2 (62 μM). Aliphatic substituents (**132**–**134**) on the oxadiazole gave some activity against *T. cruzi* with pIC_50_ values around 4.9. However, aromatic substituents (**135**–**136**) are preferred as both phenyloxazole (**135**) and 3‐fluorophenyl (**136**) showed activities around pIC_50_5.8. Finally, the thiazoles showed decent activities (pIC_50_ around 5.8) but toxicity of these compounds against MRC‐5 cells is equally high, making these compounds less favorable for future studies.

## Conclusion

Multiple approaches were explored to optimize the activity of NPD‐0227 (**3**). Modification of the core phenyl moiety delivered interesting SAR data, but activity was generally quite low, with dihydrobenzofuran (**59**) performing best with a pIC_50_ value of 5.8. Replacing the *gem*‐dimethyl moiety with several *spiro*‐analogues resulted in four analogues with a maximum activity of 5.2 (pIC_50_) hence showing substantially lower activities than NPD‐0227 (pIC_50_=6.4). The sulfur analogue of NPD‐0227, dihydropyrazolethione **99** showed a similar activity as NPD‐0227 (**3**) with a pIC_50_ of 6.3, however this was accompanied with an increase in cytotoxicity. As these compounds could not match the activity of NPD‐0227, no further screenings were done on other strains or life stages, focusing on SAR of these series. Introduction of heterocycles instead of the pyrazolone moiety also yielded several compounds with promising activities, amongst which oxazole **123** showed a pIC_50_ value of 6.2 (SI>100‐fold over MRC‐5 cytotoxicity) and oxadiazole **135** which had a pIC_50_ value of 5.8. Although these compounds do not show higher activities then optimized lead NPD‐0227 (**3**), these scaffold hops could be new starting points for future hit‐to‐lead optimization, especially with the promising selectivity index seen for oxadiazole **123**.

## Experimental Section

### Biology

*Trypanosoma cruzi in vitro assay*: Bloodstream trypomastigotes (BT) of the Y strain of *T. cruzi* were obtained by cardiac puncture of infected Swiss Webster mice on the parasitaemia peak.^12^ For the standard *in vitro* susceptibility assay on intracellular amastigotes, *T. cruzi* Tulahuen CL2, β‐galactosidase strain (DTU VI, nifurtimox‐sensitive) was used. The strain is maintained on MRC‐5_SV2_ (human lung fibroblast) cells in MEM medium, supplemented with 200 mM L‐glutamine, 16.5 mM NaHCO_3_, and 5 % heat inactivated fetal calf serum (FCSi). After incubation at 37 °C for 7 d, parasite growth was assessed by adding the α‐galactosidase substrate chlorophenol red‐α‐d‐galactopyranoside. The color reaction was read at 540 nm after 4 h, and absorbance values were expressed as a percentage of the blank controls. All cultures and assays are conducted at 37 °C under 5 % CO_2_.^13^ Benznidazole was used as a reference compound.

*MRC‐5 cytotoxicity in vitro assay*: MRC‐5‐_SV2_ cells, originally from a human diploid lung cell line, were cultivated in MEM, supplemented with L‐glutamine (20 mM), 16.5 mM sodium hydrogen carbonate and 5 % FCSi. For the assay, 10^4^ cells/well were seeded onto the test plates containing the pre‐diluted sample and incubated at 37 °C and 5 % CO_2_ for 72 h. Cell viability was assessed fluorometrically 4 h after addition of resazurin (excitation 550 nm, emission 590 nm). The results are expressed as percentage reduction in cell viability compared to untreated controls. Tamoxifen was used as a reference compound.

### Chemistry

Chemicals and reagents were obtained from commercial suppliers and were used without further purification. Anhydrous DMF, THF and CH_2_Cl_2_ were obtained by passing them through an activated alumina column prior to use. Microwave reactions were executed using a Biotage® Initiator microwave system. ^1^H NMR spectra were recorded on a Bruker Avance 250 (250 MHz), Bruker Avance 400 (400 MHz), Bruker Avance 500 (500 MHz) or Bruker 600 Avance (600 MHz) spectrometer. Chemical shifts are reported in ppm with the natural abundance of deuterium in the solvent as the internal reference (CDCl_3_: *δ* 7.26, (CD_3_)_2_SO: *δ* 2.50). ^13^C NMR spectra were recorded on a Bruker Avance 500 (126 MHz) or Bruker Avance 600 (150 MHz). Chemical shifts are reported in ppm with the solvent resonance resulting from incomplete deuteration as the internal reference (CDCl_3_: *δ* 77.16 or (CD_3_)_2_SO: *δ* 39.52). Systematic names for molecules according to IUPAC rules were generated using the Chemdraw AutoName program. LC‐MS data was gathered using a Shimadzu HPLC/MS workstation with a LC‐20AD pump system, SPD‐M20A diode array detection, and a LCMS‐2010 EV mass spectrometer. The column used is an Xbridge C_18_ 5 μm column (100 mm×4.6 mm). Solvents used were the following: solvent B=ACN, 0.1 % formic Acid; solvent A=water, 0.1 % formic acid. The analysis was conducted using a flow rate of 1.0 mL/min, start 5 % B, linear gradient to 90 % B in 4.5 min, then 1.5 min at 90 % B, linear gradient to 5 % B in 0.5 min and then 1.5 min at 5 % B, total run time of 8 min. All reported compounds have purities >95 %, measured at 254 nm, unless otherwise mentioned. All HRMS spectra were recorded on a Bruker micrOTOF mass spectrometer using ESI in positive‐ion mode. Column purifications were either carried out automatically using Biotage equipment or manually, using 60–200 mesh silica. TLC analyses were performed with Merck F254 alumina silica plates using UV visualization. All reactions were done under N_2_ atmosphere, unless specifically mentioned.

### Experimental data

Compounds **10**, **23**, **35**, **47**, **59**, **110**, **115**, **120**, **125**–**127** and **132** are reported in the main article. The experimental data of the other compounds can be found in the Supporting Information.

#### 7‐Bromo‐2,3‐dihydrobenzofuran‐5‐carboxylic acid (10)


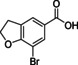


2,3‐Dihydrobenzofuran‐5‐carboxylic acid (5 g, 31 mmol) was dissolved in dioxane (100 mL), and bromine (3.14 mL, 61 mmol) was added. The reaction was stirred for 16 h after which it was quenched with sodium bisulfate. Solids were collected and recrystallized from MeOH yielding 7 g (29 mmol, 95 %) of the title compound as a white solid.^1^H NMR (600 MHz, [D_6_]DMSO) *δ* 12.86 (s, 1H), 7.85 (s, 1H), 7.78 (s, 1H), 4.72 (t, *J=*8.8 Hz, 2H), 3.34 (app. s, overlap with H_2_O peak, 3H); ^13^C NMR (151 MHz, [D_6_]DMSO) *δ* 166.4, 161.1, 133.1, 130.2, 126.2, 125.3, 101.6, 73.1, 29.9; LC‐MS (ESI) *m*/*z* found: no mass observed; *t*_R_=3.67 min.

#### Methyl 3‐(7‐bromo‐2,3‐dihydrobenzofuran‐5‐yl)‐2,2‐dimethyl‐3‐oxopropanoate (23)


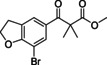


Benzoic acid **10** (2.0 g, 8.2 mmol) was dissolved in CH_2_Cl_2_ (100 mL). The mixture was stirred at RT, and oxalyl dichloride (1.1 mL, 12.3 mmol) and a few drops of DMF were added After 3 h, volatiles were evaporated, and the mixture was redissolved in 30 mL of THF. In a separate flask methyl isobutyrate (1.41 mL, 12.3 mmol) was stirred in THF (100 mL) at −78 °C and 2 M LDA (4.94 mL, 9.87 mmol) was added. After 30 min of stirring, the previously prepared acid chloride in THF was added dropwise, maintaining the temperate at −78 °C. The mixture was allowed to warm up to RT after which the mixture was quenched with sat. aq. NH_4_Cl (200 mL), extracted with Et_2_O (300 mL) and dried over MgSO_4_.The resulting crude was used in the next step without further purification. Crude ^1^H NMR (600 MHz, CDCl_3_) *δ* 7.85 (d, *J=*1.4 Hz, 1H), 7.61 (d, 1H), 4.75 (t, *J=*8.8 Hz, 2H), 3.67 (s, 3H), 3.34 (t, *J=*8.8 Hz, 2H), 1.52 (s, 7H); ^13^C NMR (151 MHz, CDCl_3_) *δ* 194.9, 175.6, 161.1, 133.3, 129.7, 128.6, 124.6, 102.7, 72.6, 53.1, 52.7, 30.1, 24.1.

#### 3‐(7‐Bromo‐2,3‐dihydrobenzofuran‐5‐yl)‐4,4‐dimethyl‐1H‐pyrazol‐5(4H)‐one (35)


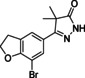


Crude keto‐ester **23** (1.0 g, 3.0 mmol) was dissolved in ethanol (10 mL), and hydrazine hydrate (1.6 mL, 31 mmol) was added. The mixture was stirred overnight, after which 50 mL of water was added and the precipitate was collected yielding 800 mg (2.6 mmol, 83 % over two steps) of the title compound as a white solid. ^1^H NMR (500 MHz, [D_6_]DMSO) *δ* 11.47 (s, 1H), 7.71–7.65 (m, 2H), 4.68 (t, *J=*8.8 Hz, 2H), 3.36 (t, *J=*8.8 Hz, 2H), 1.34 (s, 6H); ^13^C NMR (126 MHz, [D_6_]DMSO) *δ* 181.0, 161.0, 158.5, 130.4, 128.6, 125.8, 122.6, 102.3, 72.6, 46.8, 30.4, 22.3; LC‐MS (ESI) *m*/*z* found: 309 [*M*+H]^+^; *t*_R_=3.91 min; HRMS‐ESI [*M*+H]^+^ calcd for C_13_H_14_BrN_2_O_2_: 309.0233, found: 309.0232.

#### 3‐(7‐Bromo‐2,3‐dihydrobenzofuran‐5‐yl)‐1‐isopropyl‐4,4‐dimethyl‐1H‐pyrazol‐5(4H)‐one (47)


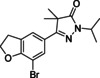


Dihydropyrazolone **35** (500 mg, 1.62 mmol) was stirred in DMF (5 mL), and sodium hydride (60 % in mineral oil; 68 mg, 1.70 mmol) was added. Stirred for 10 min, after which 2‐bromopropane (0.15 mL, 1.62 mmol) was added and the reaction was stirred for 18 h. The reaction was quenched with water, extracted with EtOAc (40 mL), which was washed with water (2×20 mL) and brine (20 mL). The resulting crude was purified over SiO_2_ using a gradient of 80 % c‐hexane in EtOAc towards EtOAc to yield 475 mg (1.3 mmol, 80 %) of the title compound as a white solid. ^1^H NMR (600 MHz, CDCl_3_) *δ* 7.74 (s, 1H), 7.63 (s, 1H), 4.75 (t, *J=*8.8 Hz, 2H), 4.52 (hept, *J=*6.7 Hz, 1H), 3.39 (t, *J=*8.8 Hz, 2H), 1.47 (s, 6H), 1.38 (d, *J=*6.7 Hz, 6H); ^13^C NMR (151 MHz, CDCl_3_) *δ* 177.7, 160.5, 158.6, 129.5, 128.9, 125.7, 121.9, 102.7, 72.1, 48.8, 45.3, 30.5, 22.6, 20.8; LC‐MS (ESI) *m*/*z* found: 351 [*M*+H]^+^; *t*_R_=4.97 min; HRMS‐ESI [*M*+H]^+^ calcd for C_16_H_20_BrN_2_O_2_: 351.0703, found: 351.0686.

#### 1‐Isopropyl‐4,4‐dimethyl‐3‐(7‐(pyridin‐3‐yl)‐2,3‐dihydrobenzofuran‐5‐yl)‐1H‐pyrazol‐5(4H)‐one (59)


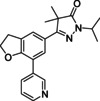


Dihydropyrazolone **47** (100 mg, 0.28 mmol) and pyridin‐3‐ylboronic acid (53 mg, 0.43 mmol) were charged to a microwave vial, after which DME (3.5 mL) and 1 M Na_2_CO_3_ (1.1 mL, 1.1 mmol) were added. The mixture was degassed with N_2_ for 5 min after which Pd(dppf)Cl_2_ (23 mg, 0.03 mmol) was added. The reaction was heated in the microwave for 1 h at 120 °C. The reaction mixture was diluted with MTBE and filtered over Celite. The residue was washed with saturated NaHCO_3_ (2×) and brine (1×). The organic phase was dried over Na_2_SO_4_, filtered and concentrated *in vacuo* to be further purified over SiO_2_ using a gradient of 20 % EtOAc in heptane towards 80 % EtOAc and subsequently recrystallized from *i*PrOH/H_2_O (2:1) to yield 39 mg (0.11 mmol, 39 %) of the title compound. ^1^H NMR (600 MHz, CDCl_3_) *δ* 8.97–8.93 (m, 1H), 8.59 (dd, *J=*5.0, 1.6 Hz, 1H), 8.07 (d, *J=*7.9 Hz, 1H), 7.74–7.71 (m, 2H), 7.41 (dd, *J=*7.8, 4.9 Hz, 1H), 4.71 (t, *J=*8.8 Hz, 2H), 4.52 (hept, *J=*6.7 Hz, 1H), 3.34 (t, *J=*8.7 Hz, 2H), 1.49 (s, 6H), 1.37 (d, *J=*6.7 Hz, 6H); ^13^C NMR (151 MHz, CDCl_3_) *δ* 177.7, 161.4, 158.8, 148.6, 147.9, 136.0, 132.8, 129.0, 126.0, 125.0, 123.5, 123.0, 119.7, 72.0, 48.9, 45.3, 29.6, 22.8, 20.8; LC‐MS (ESI) *m*/*z* found: 350 [*M*+H]^+^; *t*_R_=3.59 min; HRMS‐ESI [*M*+H]^+^ calcd for C_21_H_24_N_3_O_2_: 350.1863, found: 350.1850.

#### 3,3‐Dimethyl‐2‐oxobutyl 3‐bromo‐4‐methoxybenzoate (110)


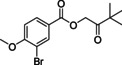


Benzoic acid **75** (1.0 g, 4.3 mmol) was added to round bottom flask and DMF (20 ml) was added, followed by sodium hydride (60 % in mineral oil) (0.18 g, 4.5 mmol). The mixture was stirred for 15 min, after which 1‐bromo‐3,3‐dimethylbutan‐2‐one (0.58 mL, 4.3 mmol) was added. The reaction mixture was stirred for another 3 h, after which it was quenched with water and extracted with MTBE (50 mL). The organic layer was washed with brine and dried over MgSO_4_. Volatiles were evaporated and recrystallization attempts from EtOH and ACN were done without success. The product was obtained as a colorless oil, yielding 1.32 g (4.0 mmol, 93 %) of the title compound. ^1^H NMR (600 MHz, CDCl_3_) *δ* 8.33–8.22 (m, 1H), 8.09–7.97 (m, 1H), 6.97–6.86 (m, 1H), 5.10 (s, 2H), 3.96 (s, 3H), 1.25 (s, 9H); ^13^C NMR (150 MHz, CDCl_3_) *δ* 207.7, 164.6, 159.7, 135.1, 131.0, 123.0, 111.5, 111.0, 64.9, 56.5, 42.9, 26.2; LC‐MS (ESI) *m*/*z* found: no mass observed; *t*_R_=4.99 min.

#### 2‐(3‐Bromo‐4‐methoxyphenyl)‐4‐(tert‐butyl)oxazole (115)


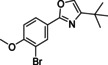


Keto‐ester **110** (500 mg, 1.52 mmol) was added to a microwave tube, followed by ammonium acetate (468 mg, 6.81 mmol) and acetic acid (5 mL). The mixture was heated in the microwave at 170 °C for 6 h, after which the reaction mixture was diluted with EtOAc and washed with sat. aq. Na_2_CO_3_ (2×50 mL) and brine (50 mL). The organic layer was dried over MgSO_4_, and volatiles were evaporated. The resulting crude was purified over SiO_2_ using a gradient of 50 % heptane in EtOAc towards 5 % MeOH in EtOAc yielding 170 mg (0.55 mmol, 36 %) of the title compound as a white solid. ^1^H NMR (600 MHz, CDCl_3_) *δ* 8.22 (s, 1H), 7.91 (d, *J=*8.4 Hz, 1H), 7.31 (s, 1H), 6.90 (d, *J=*8.5 Hz, 1H), 3.91 (s, 3H), 1.30 (s, 9H); ^13^C NMR (150 MHz, CDCl_3_) *δ* 159.9, 157.1, 151.9, 131.6, 131.4, 126.8, 122.1, 111.9, 111.6, 56.3, 31.0, 29.3; LC‐MS (ESI) *m*/*z* found: 310 [*M*+H]^+^; *t*_R_=5.75 min; HRMS‐ESI [*M*+H]^+^ calcd for C_14_H_17_BrNO_2_: 310.0437, found: 310.0439.

#### 4‐(tert‐Butyl)‐2‐(4‐methoxy‐3‐(pyridin‐3‐yl)phenyl)oxazole (120)


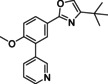


Oxazole **115** (135 mg, 0.44 mmol) and pyridin‐3‐ylboronic acid (70 mg, 0.57 mmol) were charged to a microwave vial, after which DME (4 mL) and 1 M Na_2_CO_3_ (1.3 mL, 1.3 mmol) were added. The mixture was degassed with N_2_ for 5 min, after which Pd(dppf)Cl_2_ (35 mg, 0.04 mmol) was added. The reaction was heated in the microwave for 1 h at 120 °C. The reaction mixture was diluted with EtOAc (30 mL) and filtered over Celite. The residue was washed with saturated NaHCO_3_ (2×20 mL) and brine (20 mL). The organic phase was dried over Na_2_SO_4_, filtered and concentrated *in vacuo* to be further purified over SiO_2_ using a gradient of 30 % EtOAc in heptane towards 100 % EtOAc to yield 83 mg (0.27 mmol, 62 %) of the title compound. ^1^H NMR (600 MHz, CDCl_3_) *δ* 8.79 (s, 1H), 8.55 (d, *J=*4.7 Hz, 1H), 8.03–7.96 (m, 2H), 7.86 (d, *J=*7.8 Hz, 1H), 7.35–7.28 (m, 2H), 7.01 (d, *J=*8.5 Hz, 1H), 3.83 (s, 3H), 1.29 (s, 9H); ^13^C NMR (150 MHz, CDCl_3_) *δ* 160.86, 157.86, 151.83, 150.23, 148.16, 136.87, 133.56, 131.35, 128.87, 127.84, 127.29, 122.97, 121.25, 111.18, 55.69, 31.01, 29.29; LC‐MS (ESI) *m*/*z* found: 309 [*M*+H]^+^; *t*_R_=4.13 min; HRMS‐ESI [*M*+H]^+^ calcd for C_19_H_21_N_2_O_2_: 309.1598, found: 309.1591.

#### Ethyl 3‐bromo‐4‐methoxybenzoate (125)


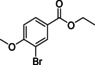


Benzoic acid **75** (10 g, 43.3 mmol) was added to a round bottom flask followed by EtOH (150 mL) and sulfuric acid (0.3 ml, 5.6 mmol). The mixture was refluxed for 6 h. Subsequently the mixture concentrated *in vacuo*, basified by slowly adding 200 mL of sat. aq. Na_2_CO_3_ and extracted with EtOAc (200 mL). The organic layer was washed with brine (150 mL) and dried over Na_2_SO_4_, after which volatiles were evaporated to give 10.9 g (42.1 mmol, 97 %) of the title compound as a white solid. ^1^H NMR (500 MHz, CDCl_3_) *δ* 8.23 (d, *J=*1.3 Hz, 1H), 7.99 (dd, *J=*8.6, 1.8 Hz, 1H), 6.91 (d, *J=*8.6 Hz, 1H), 4.35 (q, *J=*7.1 Hz, 2H), 3.95 (s, 3H), 1.38 (t, *J=*7.1 Hz, 3H); ^13^C NMR (126 MHz, CDCl_3_) *δ* 165.3, 159.4, 134.7, 130.6, 124.1, 111.4, 111.0, 61.1, 56.5, 14.4.

#### 3‐Bromo‐4‐methoxybenzohydrazide (126)


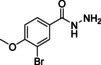


Ethyl ester **125** (6.0 g, 23.2 mmol) was dissolved in ethanol (150 mL), and hydrazine monohydrate (12.1 mL, 232 mmol) was added. The mixture was refluxed overnight after which the mixture was cooled and reduced in volume to ∼100 mL. Subsequently water (100 mL) was added and the precipitate was collected. Solids were dried *in vacuo* to yield 3.0 g (12.2 mmol, 53 %) of the title compound as a white solid. ^1^H NMR (500 MHz, CDCl_3_) *δ* 9.73 (s, 1H), 8.04 (d, *J=*2.1 Hz, 1H), 7.86 (dd, *J=*8.6, 2.1 Hz, 1H), 7.17 (d, *J=*8.6 Hz, 1H), 4.47 (s, 2H), 3.89 (s, 3H); ^13^C NMR (126 MHz, CDCl_3_) *δ* 169.4, 162.7, 136.8, 133.3, 131.9, 117.4, 115.5, 61.7. LC‐MS (ESI) *m*/*z* found: 245 [*M*+H]^+^; *t*_R_=2.68 min.

#### 2‐(3‐Bromo‐4‐methoxyphenyl)‐5‐isopropyl‐1,3,4‐oxadiazole (127)


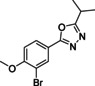


POCl_3_ (2.0 ml, 21.5 mmol) was added to hydrazide **126** (400 mg, 1.63 mmol) and isobutyric acid (144 mg, 1.63 mmol) after which the mixture was refluxed for 1 h. The reaction was quenched by pouring it over crushed ice, after which the resulting solids were collected and dried *in vacuo* to yield 380 mg (1.28 mmol, 78 %) of the title compound as a white solid. ^1^H NMR (500 MHz, CDCl_3_) *δ* 8.13 (s, 1H), 7.91 (d, *J=*8.1 Hz, 1H), 6.92 (d, *J=*8.6 Hz, 1H), 3.89 (s, 3H), 3.18 (hept, *J=*6.9 Hz, 1H), 1.37 (d, *J=*7.0 Hz, 6H); ^13^C NMR (126 MHz, CDCl_3_) *δ* 170.7, 163.3, 158.3, 131.7, 127.5, 117.9, 112.2, 111.9, 56.5, 26.5, 20.1; LC‐MS (ESI) *m*/*z* found: 297 [*M*+H]^+^; *t*_R_=4.49 min.

#### 2‐Isopropyl‐5‐(4‐methoxy‐3‐(pyridin‐3‐yl)phenyl)‐1,3,4‐oxadiazole (132)


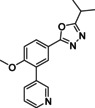


Oxadiazole **127** (100 mg, 0.34 mmol) and pyridin‐3‐ylboronic acid (54 mg, 0.44 mmol) were charged to a microwave vial after which DME (3 mL) and 1 M Na_2_CO_3_ (1.0 mL, 1.0 mmol) were added. The mixture was degassed with N_2_ for 5 min, after which Pd(dppf)Cl_2_ (14 mg, 0.02 mmol) was added. The reaction was heated in the microwave for 1 h at 120 °C. The reaction mixture was diluted with EtOAc (30 mL) and filtered over Celite. The residue was washed with saturated NaHCO_3_ (2×20 mL) and brine (20 mL). The organic phase was dried over Na_2_SO_4_, filtered and concentrated *in vacuo* to be further purified over SiO_2_ using a gradient of 40 % EtOAc in c‐hexane towards 100 % EtOAc to yield 58 mg (0.20 mmol, 58 %) of the title compound. ^1^H NMR (500 MHz, CDCl_3_) *δ* 8.80 (s, 1H), 8.60 (d, *J=*4.3 Hz, 1H), 8.07 (dd, *J=*8.6, 2.0 Hz, 1H), 7.97 (d, *J=*2.1 Hz, 1H), 7.89 (d, *J=*7.7 Hz, 1H), 7.38 (dd, *J=*7.3, 5.2 Hz, 1H), 7.10 (d, *J=*8.7 Hz, 1H), 3.90 (s, 3H), 3.26 (hept, *J=*7.0 Hz, 1H), 1.45 (d, *J=*7.0 Hz, 6H); ^13^C NMR (126 MHz, CDCl_3_) *δ* 170.6, 164.2, 159.0, 150.0, 148.2, 137.1, 133.2, 129.1, 128.5, 127.7, 123.1, 117.2, 111.6, 55.9, 26.5, 20.1; LC‐MS (ESI) *m*/*z* found: 296 [*M*+H]^+^; *t*_R_=3.10 min; HRMS‐ESI [*M*+H]^+^ calcd for C_17_H_18_N_3_O_2_: 296.1394, found: 296.1397.

## Abbreviations

AcOH: acetic acid
CDI: 1,1′‐carbonyldiimidazole
clog*P*: calculated logarithm of the partition‐coefficient
DME: dimethoxyethane
DMF: dimethylformamide
DMSO: dimethylsulfoxide
DTU: discrete typing units
DMF: dimethylformamide
dppf: 1,1′‐ferrocenediyl‐bis(diphenylphosphine)
ESI: electron spray ionization
FCS: fetal calf serum
LDA: lithium di‐isopropyl amide
MRC‐5: medical research council cell strain 5
MTBE: methyl *tert*‐butyl ether
o/n: overnight
PDE: phosphodiesterase
SAR: structure‐activity relationship
SEM: standard error of the mean
Tcr: *Trypanosoma cruzi*

## Author contributions

All molecules were synthesized and characterized by M.S., G.C. and L.M. were involved in obtaining the biological data. G.S. and R.L. contributed to the molecular design. L.M., G.S. I.J.P.E. and R.L. obtained the necessary funding to do this research.

## Conflict of interest

The authors declare no conflict of interest.

## Supporting information

As a service to our authors and readers, this journal provides supporting information supplied by the authors. Such materials are peer reviewed and may be re‐organized for online delivery, but are not copy‐edited or typeset. Technical support issues arising from supporting information (other than missing files) should be addressed to the authors.

Supplementary
