# Supplementary material for: Structure‐Activity Relationship of Phenylpyrazolones against Trypanosoma cruzi
Source: ChemMedChem. 2020 Apr 27;15(14):1310–21. doi: 10.1002/cmdc.202000136 (PMC7496920; doi:10.1002/cmdc.202000136)
Supplement: Supplementary file 1 — Supplementary [file CMDC-15-1310-s001.pdf]

ChemMedChem

Supporting Information

Structure-Activity Relationship of Phenylpyrazolones against *Trypanosoma cruzi*

Maarten Sijm, Geert Jan Sterk, Guy Caljon, Louis Maes, Iwan J. P. de Esch, and Rob Leurs*© 2020 The Authors. Published by Wiley-VCH Verlag GmbH & Co. KGaA. This is an open access article under the terms of the Creative Commons Attribution License, which permits use, distribution and reproduction in any medium, provided the original work is properly cited.

Contents

Experimental data 6

3-bromo-4,5-dimethoxybenzoic acid (**9**) 6

5-bromo-2-fluoro-4-methoxybenzoic acid (**11**) 6

3-bromo-4-methoxy-5-methylbenzoic acid (**12**) 7

5-bromo-4-methoxy-2-methylbenzoic acid (**13**) 7

methyl 3-(3-bromo-5-methoxyphenyl)-2,2-dimethyl-3-oxopropanoate (**21**) 8

methyl 3-(3-bromo-4,5-dimethoxyphenyl)-2,2-dimethyl-3-oxopropanoate (**22**) 8

methyl 3-(3,5-dibromo-4-methoxyphenyl)-2,2-dimethyl-3-oxopropanoate (**24**) 9

methyl 3-(3-bromophenyl)-2,2-dimethyl-3-oxopropanoate (**25**) 9

methyl 3-(3-bromo-4-fluorophenyl)-2,2-dimethyl-3-oxopropanoate (**26**) 10

methyl 3-(3-bromo-4-chlorophenyl)-2,2-dimethyl-3-oxopropanoate (**27**) 10

methyl 3-(3-bromo-4-methylphenyl)-2,2-dimethyl-3-oxopropanoate (**28**) 11

methyl 3-(5-bromo-2-fluoro-4-methoxyphenyl)-2,2-dimethyl-3-oxopropanoate (**29**) 11

ethyl 3-(3-bromo-2-fluoro-4-methoxyphenyl)-2,2-dimethyl-3-oxopropanoate (**30**) 12

methyl 3-(5-bromo-4-methoxy-2-methylphenyl)-2,2-dimethyl-3-oxopropanoate (**31**) 12

methyl 3-(3-bromo-4-methoxy-5-methylphenyl)-2,2-dimethyl-3-oxopropanoate (**32**) 13

3-(3-bromo-5-methoxyphenyl)-4,4-dimethyl-1H-pyrazol-5(4H)-one (**33**) 14

3-(3-bromo-4,5-dimethoxyphenyl)-4,4-dimethyl-1H-pyrazol-5(4H)-one (**34**) 14

5-(3,5-dibromo-4-methoxyphenyl)-4,4-dimethyl-2,4-dihydro-3H-pyrazol-3-one (**36**) 14

3-(3-bromophenyl)-4,4-dimethyl-1H-pyrazol-5(4H)-one (**37**) 15

3-(3-bromo-4-fluorophenyl)-4,4-dimethyl-1H-pyrazol-5(4H)-one (**38**) 15

3-(3-bromo-4-chlorophenyl)-4,4-dimethyl-1H-pyrazol-5(4H)-one (**39**) 15

3-(3-bromo-4-methylphenyl)-4,4-dimethyl-1H-pyrazol-5(4H)-one (**40**) 16

3-(3-bromo-2-fluoro-4-methoxyphenyl)-4,4-dimethyl-1H-pyrazol-5(4H)-one (**41**) 16

3-(5-bromo-2-fluoro-4-methoxyphenyl)-4,4-dimethyl-1H-pyrazol-5(4H)-one (**42**) 17

3-(3-bromo-4-methoxy-5-methylphenyl)-4,4-dimethyl-1H-pyrazol-5(4H)-one (**43**) 17

3-(5-bromo-4-methoxy-2-methylphenyl)-4,4-dimethyl-1H-pyrazol-5(4H)-one (**44**) 18

3-(3-bromo-5-methoxyphenyl)-1-isopropyl-4,4-dimethyl-1H-pyrazol-5(4H)-one (**45**)....................18

3-(3-bromo-4,5-dimethoxyphenyl)-1-isopropyl-4,4-dimethyl-1H-pyrazol-5(4H)-one (**46**)............19

5-(3,5-dibromo-4-methoxyphenyl)-2-isopropyl-4,4-dimethyl-2,4-dihydro-3H-pyrazol-3-one (**48**) 19

3-(3-bromophenyl)-1-isopropyl-4,4-dimethyl-1H-pyrazol-5(4H)-one (**49**) 20

3-(3-bromo-4-fluorophenyl)-1-isopropyl-4,4-dimethyl-1H-pyrazol-5(4H)-one (**50**).......................21

3-(3-bromo-4-chlorophenyl)-1-isopropyl-4,4-dimethyl-1H-pyrazol-5(4H)-one (**51**).......................21

3-(3-bromo-4-methylphenyl)-1-isopropyl-4,4-dimethyl-1H-pyrazol-5(4H)-one (**52**).......................22

3-(3-bromo-2-fluoro-4-methoxyphenyl)-1-isopropyl-4,4-dimethyl-1H-pyrazol-5(4H)-one (**53**) 22

3-(5-bromo-2-fluoro-4-methoxyphenyl)-1-isopropyl-4,4-dimethyl-1H-pyrazol-5(4H)-one (**54**) 23

3-(3-bromo-4-methoxy-5-methylphenyl)-1-isopropyl-4,4-dimethyl-1H-pyrazol-5(4H)-one (**55**)...24

3-(5-bromo-4-methoxy-2-methylphenyl)-1-isopropyl-4,4-dimethyl-1H-pyrazol-5(4H)-one (**56**)...25

1-isopropyl-3-(3-methoxy-5-(pyridin-3-yl)phenyl)-4,4-dimethyl-1H-pyrazol-5(4H)-one (**57**) 25

3-(3,4-dimethoxy-5-(pyridin-3-yl)phenyl)-1-isopropyl-4,4-dimethyl-1H-pyrazol-5(4H)-one (**58**) 26

5-(3-bromo-4-methoxy-5-(pyridin-3-yl)phenyl)-2-isopropyl-4,4-dimethyl-2,4-dihydro-3H-pyrazol-3-one (**60**)...27

1-isopropyl-4,4-dimethyl-3-(3-(pyridin-3-yl)phenyl)-1H-pyrazol-5(4H)-one (**61**)..........................28

3-(4-fluoro-3-(pyridin-3-yl)phenyl)-1-isopropyl-4,4-dimethyl-1H-pyrazol-5(4H)-one (**62**) 29

3-(4-chloro-3-(pyridin-3-yl)phenyl)-1-isopropyl-4,4-dimethyl-1H-pyrazol-5(4H)-one (**63**)...........30

1-isopropyl-4,4-dimethyl-3-(4-methyl-3-(pyridin-3-yl)phenyl)-1H-pyrazol-5(4H)-one (**64**)..........31

5-(2-fluoro-4-methoxy-3-(pyridin-3-yl)phenyl)-2-isopropyl-4,4-dimethyl-2,4-dihydro-3H-pyrazol-3-one (**65**)...32

3-(2-fluoro-4-methoxy-5-(pyridin-3-yl)phenyl)-1-isopropyl-4,4-dimethyl-1H-pyrazol-5(4H)-one (**66**)...33

1-isopropyl-3-(4-methoxy-3-methyl-5-(pyridin-3-yl)phenyl)-4,4-dimethyl-1H-pyrazol-5(4H)-one (**67**)...34

1-isopropyl-3-(4-methoxy-2-methyl-5-(pyridin-3-yl)phenyl)-4,4-dimethyl-1H-pyrazol-5(4H)-one (**68**)...34

methyl 3-(4-bromo-3-chlorophenyl)-2,2-dimethyl-3-oxopropanoate (**70**) 35

3-(4-bromo-3-chlorophenyl)-4,4-dimethyl-1H-pyrazol-5(4H)-one (**71**) 36

3-(4-bromo-3-chlorophenyl)-1-isopropyl-4,4-dimethyl-1H-pyrazol-5(4H)-one (**72**).......................36

2-chloro-4-(1-isopropyl-4,4-dimethyl-5-oxo-4,5-dihydro-1H-pyrazol-3-yl)benzonitrile (**73**)......... 37

4-(1-isopropyl-4,4-dimethyl-5-oxo-4,5-dihydro-1H-pyrazol-3-yl)-2-(pyridin-3-yl)benzonitrile (**74**) 38

ethyl 3-(3-bromo-4-methoxyphenyl)-3-oxopropanoate (**76**) 39

ethyl 1-(3-bromo-4-methoxybenzoyl)cyclopentanecarboxylate (**77**) 39

methyl 1-(3-bromo-4-methoxybenzoyl)cyclopent-3-enecarboxylate (**78**)......... 40

methyl 4-(3-bromo-4-methoxybenzoyl)-1-methylpiperidine-4-carboxylate (**79**)......... 41

methyl 4-(3-bromo-4-methoxybenzoyl)tetrahydro-2H-pyran-4-carboxylate (**80**)......... 41

4-(3-bromo-4-methoxyphenyl)-2,3-diazaspiro[4.4]non-3-en-1-one (**81**) 42

4-(3-bromo-4-methoxyphenyl)-2,3-diazaspiro[4.4]nona-3,7-dien-1-one (**82**) 43

4-(3-bromo-4-methoxyphenyl)-8-methyl-2,3,8-triazaspiro[4.5]dec-3-en-1-one (**83**)......... 43

4-(3-bromo-4-methoxyphenyl)-8-oxa-2,3-diazaspiro[4.5]dec-3-en-1-one (Tcr385) (**84**)......... 44

4-(3-bromo-4-methoxyphenyl)-2-isopropyl-2,3-diazaspiro[4.4]non-3-en-1-one (**85**) 44

4-(3-bromo-4-methoxyphenyl)-2-isopropyl-2,3-diazaspiro[4.4]nona-3,7-dien-1-one (Tcr403) (**86**) 45

4-(3-bromo-4-methoxyphenyl)-2-isopropyl-8-methyl-2,3,8-triazaspiro[4.5]dec-3-en-1-one (Tcr402) (**87**)......... 46

4-(3-bromo-4-methoxyphenyl)-2-isopropyl-8-oxa-2,3-diazaspiro[4.5]dec-3-en-1-one (**88**)......... 46

2-isopropyl-4-(4-methoxy-3-(pyridin-3-yl)phenyl)-2,3-diazaspiro[4.4]non-3-en-1-one (**89**)......... 47

2-isopropyl-4-(4-methoxy-3-(pyridin-3-yl)phenyl)-2,3-diazaspiro[4.4]nona-3,7-dien-1-one (**90**) .. 48

2-isopropyl-4-(4-methoxy-3-(pyridin-3-yl)phenyl)-8-methyl-2,3,8-triazaspiro[4.5]dec-3-en-1-one (**91**)......... 49

2-isopropyl-4-(4-methoxy-3-(pyridin-3-yl)phenyl)-8-oxa-2,3-diazaspiro[4.5]dec-3-en-1-one (**92**) 50

5-(3-bromo-4-methoxyphenyl)-1H-pyrazol-3-ol (**93**) 51

6-(3-bromo-4-methoxyphenyl)-2,3-dihydropyrazolo[5,1-b]oxazole (**94**) 51

6-(4-methoxy-3-(pyridin-3-yl)phenyl)-2,3-dihydropyrazolo[5,1-b]oxazole (**95**)......... 52

2-(4-methoxy-3-(pyridin-3-yl)phenyl)acetonitrile (**97**) 53

5-(4-methoxy-3-(pyridin-3-yl)benzyl)-1,3,4-thiadiazol-2-amine (**98**)......... 53

2-isopropyl-5-(4-methoxy-3-(pyridin-3-yl)phenyl)-4,4-dimethyl-2,4-dihydro-3H-pyrazole-3-thione (**99**)......... 54

4-(3-bromo-4-methoxyphenyl)-4-oxobutanoic acid (**101**) 55

6-(3-bromo-4-methoxyphenyl)-4,5-dihydropyridazin-3(2H)-one (**102**) 55

6-(3-bromo-4-methoxyphenyl)-2-isopropyl-4,5-dihydropyridazin-3(2H)-one (**103**) 56

2-isopropyl-6-(4-methoxy-3-(pyridin-3-yl)phenyl)-4,5-dihydropyridazin-3(2H)-one (**104**) 57

3-(3-bromo-4-methoxyphenyl)-4,4-dimethyl-1H-pyrazole-5(4H)-thione (**106**) 57

3-(3-bromo-4-methoxyphenyl)-5-hydrazono-4,4-dimethyl-4,5-dihydro-1H-pyrazole (**107**) 58

6-(3-bromo-4-methoxyphenyl)-3-cyclopropyl-7,7-dimethyl-7H-pyrazolo[5,1-c][1,2,4]triazole (**108**) 58

3-cyclopropyl-6-(4-methoxy-3-(pyridin-3-yl)phenyl)-7,7-dimethyl-7H-pyrazolo[5,1-c][1,2,4]triazole (**109**) 59

2-oxo-2-phenylethyl 3-bromo-4-methoxybenzoate (**111**) 60

2-(3-fluorophenyl)-2-oxoethyl 3-bromo-4-methoxybenzoate (**112**) 61

2-(4-fluorophenyl)-2-oxoethyl 3-bromo-4-methoxybenzoate (**113**) 61

1-oxo-1-phenylpropan-2-yl 3-bromo-4-methoxybenzoate (**114**) 62

2-(3-bromo-4-methoxyphenyl)-4-phenyloxazole (**116**) 63

2-(3-bromo-4-methoxyphenyl)-4-(3-fluorophenyl)oxazole) MSMTcr676) (**117**) 63

2-(3-bromo-4-methoxyphenyl)-4-(4-fluorophenyl)oxazole (**118**) 64

2-(3-bromo-4-methoxyphenyl)-5-methyl-4-phenyloxazole (**119**) 65

2-(4-methoxy-3-(pyridin-3-yl)phenyl)-4-phenyloxazole (**121**) 65

4-(3-fluorophenyl)-2-(4-methoxy-3-(pyridin-3-yl)phenyl)oxazole (**122**) 66

4-(4-fluorophenyl)-2-(4-methoxy-3-(pyridin-3-yl)phenyl)oxazole (**123**) 67

2-(4-methoxy-3-(pyridin-3-yl)phenyl)-5-methyl-4-phenyloxazole (**124**) 68

2-(3-bromo-4-methoxyphenyl)-5-cyclopentyl-1,3,4-oxadiazole (**128**) 69

2-(3-bromo-4-methoxyphenyl)-5-((tetrahydro-2H-pyran-4-yl)methyl)-1,3,4-oxadiazole (**129**) 69

2-(3-bromo-4-methoxyphenyl)-5-phenyl-1,3,4-oxadiazole (**130**) 70

2-(3-bromo-4-methoxyphenyl)-5-(3-fluorophenyl)-1,3,4-oxadiazole (**131**) 70

2-cyclopentyl-5-(4-methoxy-3-(pyridin-3-yl)phenyl)-1,3,4-oxadiazole (**133**) 71

2-(4-methoxy-3-(pyridin-3-yl)phenyl)-5-((tetrahydro-2H-pyran-4-yl)methyl)-1,3,4-oxadiazole (**134**) 72

2-(4-methoxy-3-(pyridin-3-yl)phenyl)-5-phenyl-1,3,4-oxadiazole (**135**) 73

2-(3-fluorophenyl)-5-(4-methoxy-3-(pyridin-3-yl)phenyl)-1,3,4-oxadiazole (**136**) 74

3-bromo-4-methoxybenzamide (**137**) 75

3-bromo-4-methoxybenzothioamide (**138**) 75

2-(3-bromo-4-methoxyphenyl)-4-phenylthiazole (**140**)... 76

4-cyclopropyl-2-(4-methoxy-3-(pyridin-3-yl)phenyl)thiazole (**141**) 77

2-(4-methoxy-3-(pyridin-3-yl)phenyl)-4-phenylthiazole (**142**) 77

Experimental data

Compounds **10**, **23**, **35**, **47**, **59**, **110**, **115**, **120**, **125-127** and **132** are reported in the main article. The experimental data of the other compounds can be found here.

3-bromo-4,5-dimethoxybenzoic acid (**9**)

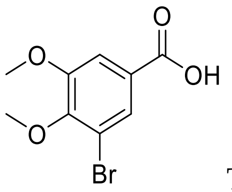

To a solution of KH_2PO_3 buffer (50 mL, pH = 7) 3-bromo-4,5-dimethoxybenzaldehyde (5 g, 20 mmol) and 2-methylpropan-2-ol (90 mL, 40 mmol) were added followed by a 1M potassium permanganate solution in water (50 mL, 50.0 mmol). The reaction was stirred for 16 hours after which it was quenched with sodium bisulfate. Precipitate was collected and recrystallized twice from MeOH yielding 4.2 g (16.1 mmol, 79%) of the title compound. ^{1}H NMR (600 MHz, DMSO-*d*6) δ 13.38 – 13.14 (m, 1H), 7.70 (d, *J* = 1.8 Hz, 1H), 7.54 (d, *J* = 1.8 Hz, 1H), 3.89 (s, 3H), 3.81 (s, 3H). ^{13}C NMR (151 MHz, DMSO-*d*6) δ 166.2, 153.6, 149.8, 128.2, 125.9, 116.9, 113.4, 60.7, 56.7. LC-MS (ESI) *m*/*z* found: no mass observed; retention time: 3.81 minutes.

5-bromo-2-fluoro-4-methoxybenzoic acid (**11**)

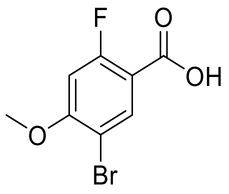

2-fluoro-4-methoxybenzoic acid (10.0 g, 58.8 mmol) was suspended in acetic acid (80 mL) after which Br_2 (6.36 mL, 123 mmol) in acetic acid (20 mL) was added dropwise at room temperature. Stirred for 3 hours, after which the mixture was heated to 60°C for 4 hours. Subsequently the reaction mixture was quenched with sodium thiosulfate and

precipirated solids were collected. After two recrystallizations from MeOH, 11.0 g (44 mmol, 75%) of the title compound was obtained as a white solid.^{1}H NMR (600 MHz, DMSO-*d*6) δ 13.21 (s, 1H), 8.00 (d, *J* = 7.9 Hz, 1H), 7.16 (d, *J* = 12.7 Hz, 1H), 3.94 (s, 3H). ^{13}C NMR (151 MHz, DMSO-*d*6) δ 164.1 (d, *J* = 4 Hz), 162.5 (d, *J* = 258 Hz), 160.4 (d, *J* = 11 Hz), 135.5, 112.7 (d, *J* = 11 Hz), 105.6 (d, *J* = 3 Hz), 102.7 (d, *J* = 28 Hz), 57.8. LC-MS (ESI) *m*/*z* found: no mass observed; retention time: 3.66 minutes.

3-bromo-4-methoxy-5-methylbenzoic acid (**12**)

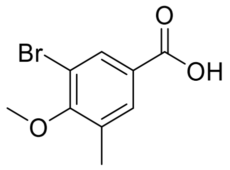

4-methoxy-3-methylbenzoic acid (4 g, 24.07 mmol) was dissolved in dioxane (100 mL) and Br_2 (4.96 mL, 96 mmol) was added. The reaction was stirred for 10 days after which it was quenched with sodium thiosulfate. Solids were collected and recrystallized twice from MeOH yielding 4.0 g (16.3 mmol, 68%) of the title compound as a white solid. ^{1}H NMR (600 MHz, DMSO-*d*6) δ 7.93 (d, 1H), 7.80 (d, 1H), 3.78 (s, 3H), 2.33 (s, 3H). ^{13}C NMR (151 MHz, DMSO-*d*6) δ 166.2, 159.0, 133.9, 132.3, 132.2, 128.4, 117.0, 60.5, 16.7. LC-MS (ESI) *m*/*z* found: no mass observed; retention time: 4.01 minutes.

5-bromo-4-methoxy-2-methylbenzoic acid (**13**)

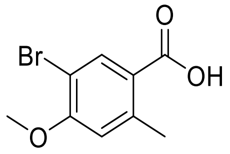

4-methoxy-2-methylbenzoic acid (4 g, 24.07 mmol) was dissolved in chloroform (100 mL) and Br_2 (1.240 mL, 24.07 mmol) and iron powder (0.134 g, 2.407 mmol) were added. The mixture was stirred for 36 hours after which the reaction was quenched with sodium thiosulfate. From this biphasic mixture precipitated solids were collected, which upon NMR revealed to be only the 1,2,4,5-substituted product. Upon acidification of the aquaous layer with 2N HCl and extraction with DCM a 2:1 ratio (based on NMR) of 1,2,4,5: 1,2,3,4

substituted products was found in the organic layer. The collected solids were recrystallized from MeOH yielding 2.1 g (8.5 mmol, 35%) of the title compound as purple crystals. ^{1}H NMR (600 MHz, DMSO-*d*6) δ 12.77 (s, 1H), 8.00 (s, 1H), 7.05 (s, 1H), 3.90 (s, 3H), 2.53 (s, 3H). ^{13}C NMR (151 MHz, DMSO-*d*6) δ 167.3, 158.2, 142.5, 135.4, 123.8, 115.8, 107.5, 57.0, 22.1. LC-MS (ESI) *m*/*z* found: no mass observed; retention time: 3.90 minutes.

methyl 3-(3-bromo-5-methoxyphenyl)-2,2-dimethyl-3-oxopropanoate (**21**)

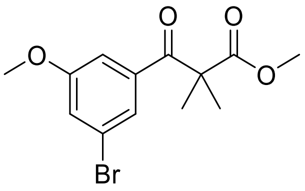

3-bromo-5-methoxybenzoic acid (2.5 g, 10.8 mmol) was dissolved in DCM (100 mL) and oxalyl dichloride (1.2 mL, 14.1 mmol) and a few drops of DMF were added. After 3 hours volatiles were evaporated and the mixture was redissolved in 60 mL of THF. In a separate flask methyl isobutyrate (1.6 mL, 14.1 mmol) was stirred in THF (100 mL) at -78°C and 2M LDA in THF/heptane/ethylbenzene (6 mL, 11.9 mmol) was added. After stirring for 30 minutes the previously prepared acid chloride in THF was added dropwise, maintaining the temperate at -78°C. The mixture was allowed to warm up to rt after which the mixture was quenched with sat. aq. NH_4Cl (300 mL), extracted with Et_2O (400 mL) and dried over $MgSO_4$.The resulting 3.0 g crude was used in the next step without further purification.

methyl 3-(3-bromo-4,5-dimethoxyphenyl)-2,2-dimethyl-3-oxopropanoate (**22**)

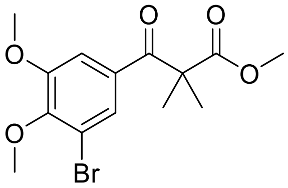

Benzoic acid **9** (2.0 g, 7.7 mmol) was dissolved in DCM (100 mL). The mixture was stirred at rt, and oxalyl dichloride (0.97 mL, 11.5 mmol) and a few drops of DMF were added After 3 hours volatiles were evaporated and the mixture was redissolved in 30 mL of THF. In a separate flask methyl isobutyrate (1.32 mL, 11.5 mmol) was stirred in THF (100

mL) at -78°C and 2M LDA (4.6 mL, 9.2 mmol) was added. After stirring for 30 minutes the previously prepared acid chloride in THF was added dropwise, maintaining the temperate at -78°C. The mixture was allowed to warm up to rt after which the mixture was quenched with sat. aq. NH_4Cl (200 mL), extracted with Et_2O (300 mL) and dried over $MgSO_4$.The resulting crude was used in the next step without further purification. Crude ^{1}H NMR (300 MHz, Chloroform-d) δ 7.59 (d, *J* = 2.0 Hz, 1H), 7.40 (d, *J* = 1.7 Hz, 1H), 3.92 (s, 3H), 3.88 (s, 3H), 3.68 (s, 3H), 1.54 (s, 6H).

methyl 3-(3,5-dibromo-4-methoxyphenyl)-2,2-dimethyl-3-oxopropanoate (**24**)

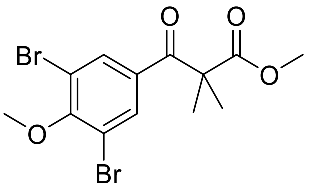

3,5-dibromo-4-methoxybenzoic acid (1.5 g, 4.8 mmol) was dissolved in DCM (100 mL). The mixture was stirred at rt, and oxalyl dichloride (0.61 mL, 7.3 mmol) and a few drops of DMF were added After 3 hours volatiles were evaporated and the mixture was redissolved in 30 mL of THF. In a separate flask methyl isobutyrate (0.83 mL, 7.3 mmol) was stirred in THF (20 mL) at -78°C and 2M LDA (2.9 mL, 5.8 mmol) was added. After stirring for 30 minutes the previously prepared acid chloride in THF was added dropwise, maintaining the temperate at -78°C. The mixture was allowed to warm up to rt after which the mixture was quenched with sat. aq. NH_4Cl (150 mL), extracted with Et_2O (200 mL) and dried over $MgSO_4$.The resulting crude was used in the next step without further purification.

methyl 3-(3-bromophenyl)-2,2-dimethyl-3-oxopropanoate (**25**)

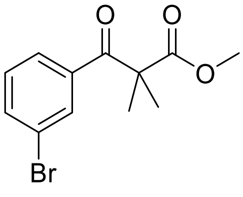

3-bromobenzoic acid (5.0 g, 24.9 mmol) was dissolved in DCM (30 mL) and oxalyl chloride (2.74 ml, 32.3 mmol) was added followed by a drop of DMF. This mixture

was stirred for 3 h, after which volatiles were evaporated and the mixture was redissolved in ~50 mL THF. In a different flask methyl isobutyrate (3.71 mL, 32.3 mmol) was dissolved in THF (10 mL) and the mixture was cooled down to -78 °C after which 2M LDA (in toluene, THF, heptane) (13.7 ml, 5.2 mmol) was added dropwise, while maintaining -78 °C. Upon completion of addition of the LDA the mixture was stirred for 15 minutes followed by the dropwise addition of the chlorinated 3-bromobenzoic acid in THF, maintaining the temperate at -78 °C. Upon completion of the addition the mixture was allowed to warm up to room temperature and the reaction was quenched by the addition of 30 mL water. The aqueous layer was extracted with Et_2O (50 mL) after which the organic layer was washed with brine (30 mL). The resulting crude was used in the next step without further purification.

methyl 3-(3-bromo-4-fluorophenyl)-2,2-dimethyl-3-oxopropanoate (**26**)

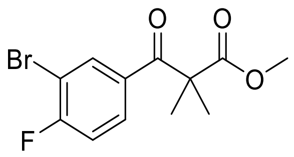

3-bromo-4-fluorobenzoic acid (5.0 g, 22.8 mmol) was dissolved in DCM (100 mL) and oxalyl dichloride (2.9 mL, 34.2 mmol) and a few drops of DMF were added. After 3 hours volatiles were evaporated and the mixture was redissolved in 60 mL of THF. In a separate flask methyl isobutyrate (3.93 mL, 34.2 mmol) was stirred in THF (100 mL) at -78°C and 2M LDA in THF/heptane/ethylbenzene (13.7 mL, 27.4 mmol) was added. After stirring for 30 minutes the previously prepared acid chloride in THF was added dropwise, maintaining the temperate at -78°C. The mixture was allowed to warm up to rt after which the mixture was quenched with sat. aq. NH_4Cl (300 mL), extracted with Et_2O (400 mL) and dried over $MgSO_4$.The resulting 6.9 g crude was used in the next step without further purification.

methyl 3-(3-bromo-4-chlorophenyl)-2,2-dimethyl-3-oxopropanoate (**27**)

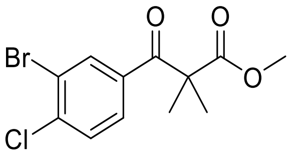
3-bromo-4-chlorobenzoic acid (5.0 g, 21.3 mmol) was dissolved in DCM (100 mL) and oxalyl dichloride (2.7 mL, 31.9 mmol) and a few drops of DMF were added. After 3 hours volatiles were evaporated and the mixture was redissolved in 60 mL of THF. In a separate flask methyl isobutyrate (3.65 mL, 31.9 mmol) was stirred in THF (100 mL) at -78°C and 2M LDA in THF/heptane/ethylbenzene (12.7 mL, 25.5 mmol) was added. After stirring for 30 minutes the previously prepared acid chloride in THF was added dropwise, maintaining the temperate at -78°C. The mixture was allowed to warm up to rt after which the mixture was quenched with sat. aq. NH_4Cl (300 mL), extracted with Et_2O (400 mL) and dried over $MgSO_4$.The resulting 6.8 g crude was used in the next step without further purification.

methyl 3-(3-bromo-4-methylphenyl)-2,2-dimethyl-3-oxopropanoate (**28**)

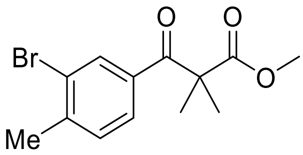
3-bromo-4-methylbenzoic acid (5.0 g, 23.3 mmol) was dissolved in DCM (100 mL) and oxalyl dichloride (3.0 mL, 34.9 mmol) and a few drops of DMF were added. After 3 hours volatiles were evaporated and the mixture was redissolved in 60 mL of THF. In a separate flask methyl isobutyrate (4.0 mL, 34.9 mmol) was stirred in THF (100 mL) at -78°C and 2M LDA in THF/heptane/ethylbenzene (14.0 mL, 28 mmol) was added. After stirring for 30 minutes the previously prepared acid chloride in THF was added dropwise, maintaining the temperate at -78°C. The mixture was allowed to warm up to rt after which the mixture was quenched with sat. aq. NH_4Cl (300 mL), extracted with Et_2O (400 mL) and dried over $MgSO_4$.The resulting crude was used in the next step without further purification.

methyl 3-(5-bromo-2-fluoro-4-methoxyphenyl)-2,2-dimethyl-3-oxopropanoate (**29**)

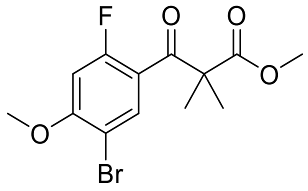

5-bromo-2-fluoro-4-methoxybenzoic acid (2.0 g, 8.03 mmol) was dissolved in DCM (100 mL) and oxalyl dichloride (1.02 mL, 12.1 mmol) and a few drops of DMF were added. After 3 hours volatiles were evaporated and the mixture was redissolved in 30 mL of THF. In a separate flask methyl isobutyrate (1.38 mL, 12.1 mmol) was stirred in THF (100 mL) at -78°C and 2M LDA in THF/heptane/ethylbenzene (4.82 mL, 9.64 mmol) was added. After stirring for 30 minutes the previously prepared acid chloride in THF was added dropwise, maintaining the temperate at -78°C. The mixture was allowed to warm up to rt after which the mixture was quenched with sat. aq. NH_4Cl (200 mL), extracted with Et_2O (300 mL) and dried over $MgSO_4$.The resulting crude was used in the next step without further purification. Crude ^1H NMR (300 MHz, Chloroform-d) δ 8.12 (d, *J* = 7.7 Hz, 1H), 6.59 (d, *J* = 12.8 Hz, 1H), 3.94 (s, 3H), 3.68 (s, 3H), 1.48 (s, 6H).

ethyl 3-(3-bromo-2-fluoro-4-methoxyphenyl)-2,2-dimethyl-3-oxopropanoate (**30**)

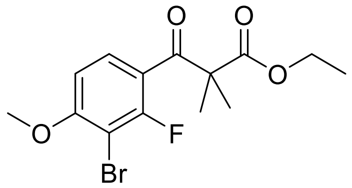

Benzoic acid **11** (1.56 g, 9.8 mmol) was dissolved in DCM (20 ml) and oxalyl chloride (0.94 ml, 10.7 mmol) and a few drops of DMF were added. After 2 h volatiles were evaporated and the resulting crude was redissolved in DCM (20 ml). Subsequently 2-bromo-1-fluoro-3-methoxybenzene (2.0 g, 9.8 mmol) and $AlCl_3$ (1.43 g, 10.7 mmol) were added and reaction was stirred at room temperature for 72 hours. The reaction was quenched in 2M HCl and extracted with DCM (3x 100 ml). Organic layers were collected and dried over $MgSO_4$. The resulting crude was used in the next step without further purification.

methyl 3-(5-bromo-4-methoxy-2-methylphenyl)-2,2-dimethyl-3-oxopropanoate (**31**)

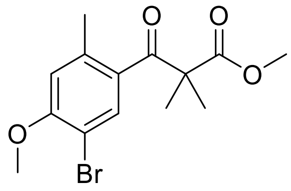

Benzoic acid **12** (1.2 g, 4.9 mmol) was dissolved in DCM (100 mL). The mixture was stirred at rt, and oxalyl dichloride (0.62 mL, 7.3 mmol) and a few drops of DMF were added After 3 hours volatiles were evaporated and the mixture was redissolved in 30 mL of THF. In a separate flask methyl isobutyrate (0.84 mL, 7.3 mmol) was stirred in THF (100 mL) at -78°C and 2M LDA (2.9 mL, 5.9 mmol) was added. After stirring for 30 minutes the previously prepared acid chloride in THF was added dropwise, maintaining the temperate at -78°C. The mixture was allowed to warm up to rt after which the mixture was quenched with sat. aq. NH_4Cl (200 mL), extracted with Et_2O (300 mL) and dried over $MgSO_4$.The resulting crude was used in the next step without further purification. Crude ^{1}H NMR (300 MHz, Chloroform-d) δ 7.52 (s, 1H), 6.74 (s, 1H), 3.91 (s, 3H), 3.68 (s, 3H), 2.38 (s, 3H), 1.49 (s, 6H).

methyl 3-(3-bromo-4-methoxy-5-methylphenyl)-2,2-dimethyl-3-oxopropanoate (**32**)

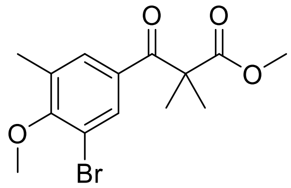

Benzoic acid **13** (2.0 g, 8.2 mmol) was dissolved in DCM (100 mL). The mixture was stirred at rt, and oxalyl dichloride (1.1 mL, 12.2 mmol) and a few drops of DMF were added After 3 hours volatiles were evaporated and the mixture was redissolved in 30 mL of THF. In a separate flask methyl isobutyrate (1.40 mL, 12.2 mmol) was stirred in THF (100 mL) at -78°C and 2M LDA (4.9 mL, 9.8 mmol) was added. After stirring for 30 minutes the previously prepared acid chloride in THF was added dropwise, maintaining the temperate at -78°C. The mixture was allowed to warm up to rt after which the mixture was quenched with sat. aq. NH_4Cl (200 mL), extracted with Et_2O (300 mL) and dried over $MgSO_4$.The resulting crude was used in the next step without further purification. Crude ^{1}H NMR (300 MHz,

Chloroform-d) δ 7.86 (d, *J* = 2.0 Hz, 1H), 7.58 (d, *J* = 1.6 Hz, 1H), 3.85 (s, 3H), 3.67 (s, 3H), 2.34 (s, 3H), 1.52 (s, 6H).

3-(3-bromo-5-methoxyphenyl)-4,4-dimethyl-1H-pyrazol-5(4H)-one (**33**)

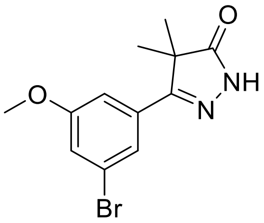

Crude keto-ester **21** (3.0 g) was dissolved in ethanol (7 mL) and hydrazine hydrate (11.4 mL, 234 mmol) was added. The mixture was stirred overnight, after which 50 mL of water was added and the precipitate was collected yielding 3.2 g of solids which were used in the following step without further purification.

3-(3-bromo-4,5-dimethoxyphenyl)-4,4-dimethyl-1H-pyrazol-5(4H)-one (**34**)

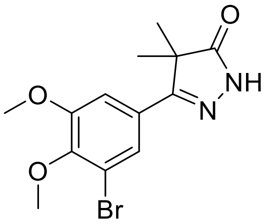

Crude keto-ester **22** (1.7 g, 4.9 mmol) was dissolved in ethanol (10 mL) and hydrazine hydrate (2.6 mL, 49 mmol) was added. Stirred for 18 h after which 10 mL of water was added. Solids were collected to yield 1.2 mg (3.7 mmol, 75% over two steps) of the title compound. ^{1}H NMR (600 MHz, DMSO-*d*6) δ 11.62 (s, 1H), 7.48 (d, *J* = 2.1 Hz, 1H), 7.36 (d, *J* = 1.7 Hz, 1H), 3.89 (s, 3H), 3.78 (s, 3H), 1.36 (s, 6H). ^{13}C NMR (151 MHz, DMSO-*d*6) δ 181.1, 160.4, 154.0, 147.39, 128.73, 121.96, 117.51, 109.82, 60.69, 56.60, 46.85, 22.39. LC-MS (ESI) *m*/*z* found: 327 $[M+H]^+$; retention time: 4.01 minutes. HRMS-ESI $[M+H]^+$ calculated for $C_{13}H_{16}BrN_2O_3$: 327.0339, found: 327.0353.

5-(3,5-dibromo-4-methoxyphenyl)-4,4-dimethyl-2,4-dihydro-3H-pyrazol-3-one (**36**)

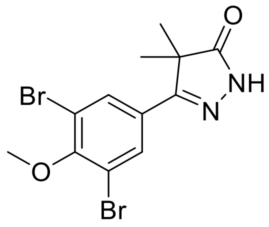

Crude keto-ester **24** (1.9 g, 3.0 mmol) was dissolved in ethanol (10 mL) and hydrazine hydrate (2.3 mL, 48 mmol) was added. The mixture was stirred overnight, after which 50 mL of water was added and the precipitate was collected yielding 720 mg of the title compound as a white solid which was used in the following steps without further purification.

3-(3-bromophenyl)-4,4-dimethyl-1H-pyrazol-5(4H)-one (**37**)

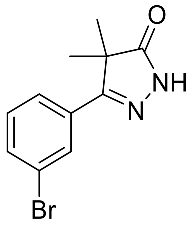

Crude keto-ester **25** (7.0 g, 24.6 mmol) was dissolved in EtOH (20 mL) and hydrazine hydrate (60-65%) (11.7 mL, 245 mmol) was added and the reaction was stirred overnight at room temperature. The reaction was quenched with water (10 mL) after precipitation was visible, solids were filtered off and dried *in vacuo* to 5.0 gram of crude which was continued in the next step without further purification.

3-(3-bromo-4-fluorophenyl)-4,4-dimethyl-1H-pyrazol-5(4H)-one (**38**)

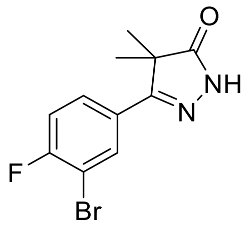

Crude **26** (6.9 g, 22.8 mmol) was dissolved in ethanol (18 mL) and hydrazine hydrate (11.1 mL, 228 mmol) was added. The mixture was stirred overnight, after which 50 mL of water was added and the precipitate was collected yielding 2.7 g of solids which were used in the following step without further purification.

3-(3-bromo-4-chlorophenyl)-4,4-dimethyl-1H-pyrazol-5(4H)-one (**39**)

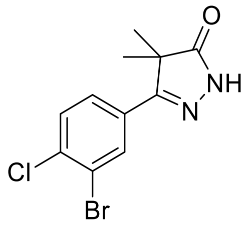

Crude **27** (6.7 g, 21.3 mmol) was dissolved in ethanol (18 mL) and hydrazine hydrate (10.4 mL, 213 mmol) was added. The mixture was stirred overnight, after which 50 mL of water was added and the precipitate was collected yielding 2.4 g of solids which were used in the following step without further purification.

3-(3-bromo-4-methylphenyl)-4,4-dimethyl-1H-pyrazol-5(4H)-one (**40**)

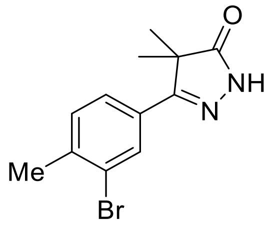

Crude **28** (7.0 g, 23.4 mmol) was dissolved in ethanol (18 mL) and hydrazine hydrate (11.4 mL, 234 mmol) was added. The mixture was stirred overnight, after which 50 mL of water was added and the precipitate was collected yielding 3.2 g of solids which were used in the following step without further purification.

3-(3-bromo-2-fluoro-4-methoxyphenyl)-4,4-dimethyl-1H-pyrazol-5(4H)-one (**41**)

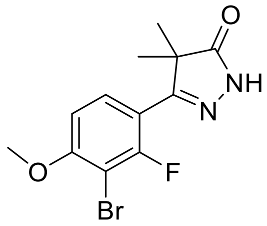

Crude keto-ester **30** (3.3 g, 9.9 mmol) was dissolved in ethanol (15 mL) and hydrazine hydrate (4.7 mL, 99 mmol) was added. The mixture was stirred overnight, after which volatiles were evaporated and the resulting crude was dissolved in EtOAc (100 mL) and washed with water (3x 100 mL) and brine (100 mL). The remaining organic layer was dried over MgSO4 and evaporated to dryness. The remaining crude was purified over SiO_2 using a gradient of 20% EtOAc in heptane towards EtOAc yielding 190 mg (0.6 mmol, 6% over two

steps) of the title compound as a white solid. ^{1}H-NMR: (500 MHz, Chloroform-*d*) δ 9.30 (s, 1H), 7.61 (t, *J* = 8.5 Hz, 1H), 6.77 (dd, *J* = 8.9, 1.4 Hz, 1H), 3.96 (s, 3H), 1.40 (d, *J* = 1.7 Hz, 6H). ^{13}C-NMR: (151 MHz, Chloroform-d) 181.1, 160.6 (d, 4.3 Hz), 158.7 (d, 4.3 Hz), 157.5 (d, 250.7 Hz), 128.9 (d, 5.2 Hz), 113.1 (d, 15,7 Hz), 107.3 (d, 3.2 Hz), 100.4 (d, 23.7 Hz), 56.8, 48.4, 21.3. LC-MS (ESI) *m/z* found: 315 [M+H]$^{+}$; retention time: 4.03 minutes. HRMS-ESI [M+H]$^{+}$ calculated for $C_{12}H_{13}BrFN_{2}O_{2}$: 315.0124, found 315.0139.

3-(5-bromo-2-fluoro-4-methoxyphenyl)-4,4-dimethyl-1H-pyrazol-5(4H)-one (**42**)

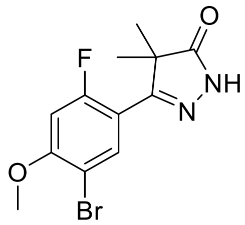

Crude keto-ester **31** (1.8 g, 5.4 mmol) was dissolved in ethanol (10 mL) and hydrazine (2.8 mL, 54 mmol) was added. Stirred for 18 h after which 10 mL of water was added. Solids were collected to yield 1.4 g (4.4 mmol, 82% over two steps) of the title compound. ^{1}H NMR (600 MHz, DMSO-*d*6) δ 11.63 (s, 1H), 7.86 (d, *J* = 7.9 Hz, 1H), 7.21 (d, *J* = 13.0 Hz, 1H), 3.92 (s, 3H), 1.23 (s, 6H). ^{13}C NMR (151 MHz, DMSO-*d*6) δ 180.7, 160.0 (d, *J* = 249 Hz), 158.5 (d, *J* = 3 Hz), 158.0 (d, *J* = 11 Hz), 132.9 (d, *J* = 5 Hz), 113.1 (d, *J* = 16 Hz), 106.4 (d, *J* = 3 Hz), 102.4 (d, *J* = 27 Hz), 57.6, 47.9, 21.3. LC-MS (ESI) *m/z* found: 315 [M+H]$^{+}$; retention time: 3.97 minutes. HRMS-ESI [M+H]$^{+}$ calculated for $C_{12}H_{13}BrFN_{2}O_{2}$: 315.0139, found: 315.0145.

3-(3-bromo-4-methoxy-5-methylphenyl)-4,4-dimethyl-1H-pyrazol-5(4H)-one (**43**)

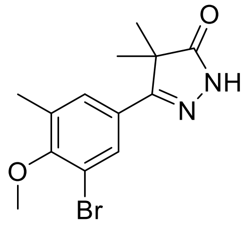

Crude keto-ester **32** (1.4 g, 4.25 mmol) was dissolved in ethanol (10 mL) and hydrazine hydrate (2.2 mL, 43 mmol) was added. The mixture was stirred overnight, after

which 50 mL of water was added and the precipitate was collected yielding 1.1 g (3.5 mmol, 83% over two steps) of the title compound as a white solid. ^{1}H NMR (600 MHz, DMSO-*d*6) δ 11.59 (s, 1H), 7.81 (d, *J* = 1.9 Hz, 1H), 7.64 (d, *J* = 1.5 Hz, 1H), 3.77 (s, 3H), 2.35 (s, 3H), 1.35 (s, 6H). ^{13}C NMR (151 MHz, DMSO-*d*6) δ 181.1, 160.5, 156.3, 134.2, 128.8, 128.5, 128.2, 117.6, 60.4, 46.8, 22.2, 16.8. LC-MS (ESI) *m/z* found: 311 $[M+H]^+$; retention time: 4.22 minutes. HRMS-ESI $[M+H]^+$ calculated for $C_{13}H_{16}BrN_2O_2$: 311.0390, found: 311.0380.

3-(5-bromo-4-methoxy-2-methylphenyl)-4,4-dimethyl-1H-pyrazol-5(4H)-one (**44**)

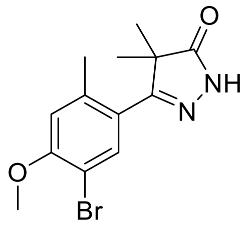

Crude keto-ester **33** (1.1 g, 3.34 mmol) was dissolved in ethanol (10 mL) and hydrazine hydrate (1.75 mL, 33.4 mmol) was added. Stirred for 18 h after which 10 mL of water was added. Solids were collected to yield 950 mg (3.0 mmol, 91% over two steps) of the title compound. ^{1}H NMR (600 MHz, DMSO-*d*6) δ 11.48 (s, 1H), 7.47 (s, 1H), 7.10 (s, 1H), 3.89 (s, 3H), 2.31 (s, 3H), 1.18 (s, 6H). ^{13}C NMR (151 MHz, DMSO-*d*6) δ 180.3, 162.5, 155.9, 139.4, 132.6, 125.2, 115.5, 107.6, 56.8, 48.6, 21.5, 21.1. LC-MS (ESI) *m/z* found: 311 $[M+H]^+$; retention time: 3.97 minutes. HRMS-ESI $[M+H]^+$ calculated for $C_{13}H_{16}BrN_2O_2$: 311.0390, found: 311.0398.

3-(3-bromo-5-methoxyphenyl)-1-isopropyl-4,4-dimethyl-1H-pyrazol-5(4H)-one (**45**)

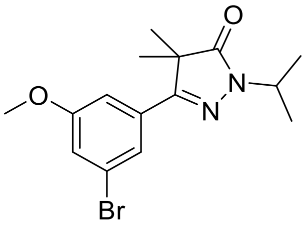

Crude dihydropyrazolone **33** (1.0 g, 3.4 mmol) was stirred in DMF (5 mL) and sodium hydride (60% in mineral oil) (148 mg, 3.70 mmol) was added. Stirred for 20 minutes, after which 2-bromopropane (0.35 mL, 3.7 mmol) was added and the mixture was

stirred for another 16 hours. The reaction was quenched with 20 mL of water and the resulting solids were collected and washed with water (20 mL) to yield 647 mg (1.9 mmol, 56% over 3 steps) of the title compound as a light yellow solid. ^{1}H-NMR: (500 MHz, Chloroform-*d*) δ 7.53 (s, 1H), 7.25 (s, 1H), 7.07 (s, 1H), 4.50 (hept, *J* = 6.7 Hz, 1H), 3.84 (s, 3H), 1.45 (s, 6H), 1.36 (d, *J* = 6.7 Hz, 6H). ^{13}C-NMR: (126 MHz, Chloroform-d) δ 177.8, 160.4, 160.0, 133.7, 123.2, 121.5, 117.8, 111.2, 55.7, 48.7, 45.4, 22.5, 20.8. LC-MS (ESI) *m*/*z* found: 339 $[M+H]^+$; retention time: 5.71 minutes, 94% purity.

3-(3-bromo-4,5-dimethoxyphenyl)-1-isopropyl-4,4-dimethyl-1H-pyrazol-5(4H)-one (**46**)

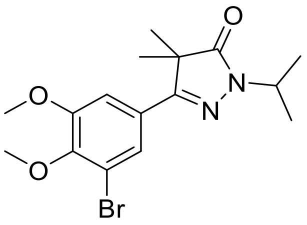

Dihydropyrazolone **34** (500 mg, 1.53 mmol) was stirred in DMF (5 mL) and sodium hydride (60% in mineral oil) (64 mg, 1.61 mmol) was added. Stirred for 10 minutes, after which 2-bromopropane (0.14 mL, 1.53 mmol) was added and the reaction was stirred for 18 hours. The reaction was quenched with water, extracted with EtOAc (40 mL), which was washed with water (2x 20 mL) and brine (20 mL). The resulting crude was purified over SiO_2 using a gradient of 80% c-hexane in EtOAc towards EtOAc to yield 390 mg (1.1 mmol, 69%) of the title compound as a white solid. ^{1}H NMR (600 MHz, Chloroform-d) δ 7.49 (d, *J* = 1.9 Hz, 1H), 7.37 (d, *J* = 1.7 Hz, 1H), 4.51 (hept, *J* = 6.7 Hz, 1H), 3.93 (s, 3H), 3.89 (s, 3H), 1.46 (s, 6H), 1.37 (d, *J* = 6.7 Hz, 6H). ^{13}C NMR (151 MHz, Chloroform-d) δ 177.7, 160.0, 153.8, 147.9, 128.2, 122.8, 117.8, 109.3, 60.7, 56.2, 48.7, 45.4, 22.7, 20.8. LC-MS (ESI) *m*/*z* found: 369 $[M+H]^+$; retention time: 5.08 minutes. HRMS-ESI $[M+H]^+$ calculated for $C_{16}H_{22}BrN_2O_2$: 369.0808, found: 369.0791.

5-(3,5-dibromo-4-methoxyphenyl)-2-isopropyl-4,4-dimethyl-2,4-dihydro-3H-pyrazol-3-one (**48**)

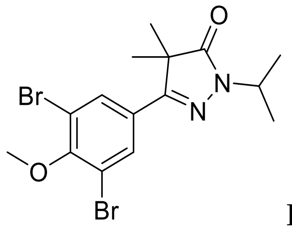

Dihydropyrazolone **36** (400 mg, 1.06 mmol) was stirred in DMF (2 mL) and sodium hydride (60% in mineral oil) (47 mg, 1.17 mmol) was added. Stirred for 10 minutes, after which 2-bromopropane (0.11 mL, 1.2 mmol) was added and the reaction was stirred for 18 hours. The reaction was quenched with water, extracted with EtOAc (40 mL), which was washed with water (2x 20 mL) and brine (20 mL). The resulting crude was purified over SiO_2 using a gradient of 80% c-hexane in EtOAc towards EtOAc to yield 350 mg (1.3 mmol, 27% over four steps) of the title compound as a white solid. LC-MS (ESI) *m/z* found: 417 $[M+H]^+$; retention time: 5.71 minutes.

3-(3-bromophenyl)-1-isopropyl-4,4-dimethyl-1H-pyrazol-5(4H)-one (**49**)

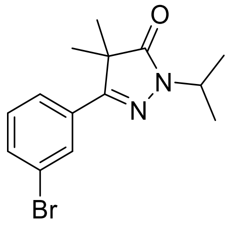

Crude dihydropyrazolone **37** (2.0 g, 7.5 mmol) was added to a round bottom flask and DMF (15 mL) was added, followed by sodium hydride (60% in mineral oil) (0.33 g, 8.2 mmol). This mixture was stirred for 30 minutes after which 2-bromopropane (0.84 mL, 9.0 mmol) was added. After stirring for 16 h at rt, the mixture was quenched in ice cold water (50 mL) and extracted with MTBE (50 mL). The organic layer was washed with brine (30 mL) and dried over Na_2SO_4, after which solids were filtered off. After evaporation of the remaining volatiles the product was purified over SiO_2 using a gradient of 10% EtOAc in c-hexane towards 40% EtOAc in c-hexane yielded 1.3 g of impure product as an off-white solid, which was used without further purification in the next step.

3-(3-bromo-4-fluorophenyl)-1-isopropyl-4,4-dimethyl-1H-pyrazol-5(4H)-one (**50**)

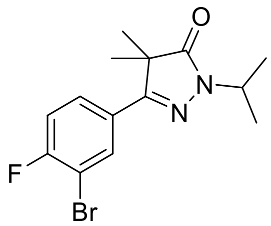

Crude dihydropyrazolone **38** (1.0 g, 3.5 mmol) was stirred in DMF (5 mL) and sodium hydride (60% in mineral oil) (154 mg, 3.86 mmol) was added. Stirred for 20 minutes, after which 2-bromopropane (0.37 mL, 3.9 mmol) was added and the mixture was stirred for another 16 hours. The reaction was quenched with 20 mL of water and the resulting solids were collected and washed with water (20 mL) to yield 990 mg (3.0 mmol, 36% over 3 steps) of the title compound as a yellow-ish solid. ^{1}H-NMR: (500 MHz, Chloroform-*d*) δ 8.05 (dd, *J* = 6.6, 2.2 Hz, 1H), 7.72 (ddd, *J* = 8.6, 4.6, 2.2 Hz, 1H), 7.18 (t, *J* = 8.4 Hz, 1H), 4.52 (hept, *J* = 6.7 Hz, 1H), 1.46 (s, 6H), 1.38 (d, *J* = 6.7 Hz, 6H). ^{13}C-NMR: (126 MHz, Chloroform-d) δ 177.6, 159.7 (d, *J* = 250 Hz), 159.3 (d, *J* = 2 Hz), 131.4, 128.9 (d, *J* = 4 Hz), 126.7 (d, *J* = 8 Hz), 116.7 (d, *J* = 22 Hz), 109.8 (d, *J* = 22 Hz), 48.6, 45.4, 22.4, 20.8. LC-MS (ESI) *m*/*z* found: 327 $[M+H]^+$; retention time: 5.24 minutes, 94% purity. HRMS-ESI $[M+H]^+$ calculated for $C_{14}H_{17}BrFN_2O$: 327.0503, found: 327.0493.

3-(3-bromo-4-chlorophenyl)-1-isopropyl-4,4-dimethyl-1H-pyrazol-5(4H)-one (**51**)

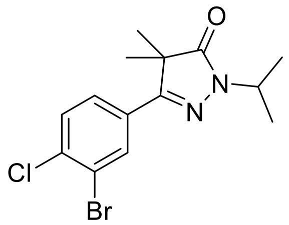

Crude dihydropyrazolone **39** (1.0 g, 3.3 mmol) was stirred in DMF (5 mL) and sodium hydride (60% in mineral oil) (146 mg, 3.65 mmol) was added. Stirred for 20 minutes, after which 2-bromopropane (0.35 mL, 3.7 mmol) was added and the mixture was stirred for another 16 hours. The reaction was quenched with 20 mL of water and the resulting solids were collected and washed with water (20 mL) to yield 880 mg (2.6 mmol, 29% over 3

steps) of the title compound as a light yellow solid. ^{1}H-NMR: (500 MHz, Chloroform-*d*) δ 8.09 (d, *J* = 2.0 Hz, 1H), 7.67 (dd, *J* = 8.5, 2.1 Hz, 1H), 7.49 (d, *J* = 8.4 Hz, 1H), 4.52 (hept, *J* = 6.7 Hz, 1H), 1.46 (s, 6H), 1.38 (d, *J* = 6.8 Hz, 6H). ^{13}C-NMR: (126 MHz, Chloroform-d) δ 177.7, 159.1, 135.7, 131.2, 131.1, 130.5, 125.8, 123.1, 48.6, 45.5, 22.4, 20.8. LC-MS (ESI) *m/z* found: 343 [M+H]$^+$; retention time: 5.59 minutes. HRMS-ESI [M+H]$^+$ calculated for $C_{14}H_{17}ClN_2O$: 343.0207, found: 343.0191.

3-(3-bromo-4-methylphenyl)-1-isopropyl-4,4-dimethyl-1H-pyrazol-5(4H)-one (**52**)

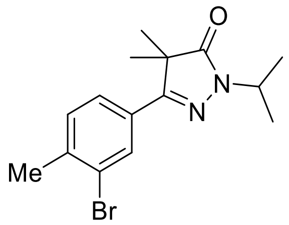

Crude dihydropyrazolone **40** (1.0 g, 3.4 mmol) was stirred in DMF (5 mL) and sodium hydride (60% in mineral oil) (148 mg, 3.7 mmol) was added. Stirred for 20 minutes, after which 2-bromopropane (0.35 mL, 3.7 mmol) was added and the mixture was stirred for another 16 hours. The reaction was quenched with 20 mL of water and the resulting solids were collected and washed with water (20 mL) to yield 647 mg (3.0 mmol, 41% over 3 steps) of the title compound as a light yellow solid. ^{1}H-NMR: (500 MHz, Chloroform-*d*) δ 8.02 (d, *J* = 1.8 Hz, 1H), 7.62 (dd, *J* = 8.0, 1.8 Hz, 1H), 7.32 – 7.21 (m, 1H), 4.52 (hept, *J* = 6.7 Hz, 1H), 2.44 (s, 3H), 1.47 (s, 6H), 1.38 (d, *J* = 6.7 Hz, 6H). ^{13}C-NMR: (126 MHz, Chloroform-d) δ 177.8, 160.2, 139.6, 130.9, 130.6, 129.9, 125.4, 124.8, 48.7, 45.3, 22.9, 22.5, 20.8. LC-MS (ESI) *m/z* found: 323 [M+H]$^+$; retention time: 5.52 minutes. HRMS-ESI [M+H]$^+$ calculated for $C_{15}H_{20}N_2O$: 323.0754, found: 323.0742.

3-(3-bromo-2-fluoro-4-methoxyphenyl)-1-isopropyl-4,4-dimethyl-1H-pyrazol-5(4H)-one (**53**)

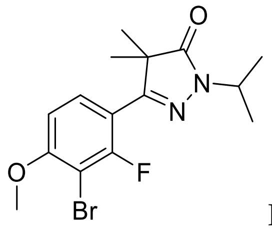

Dihydropyrazolone **41** (150 mg, 0.48 mmol) was stirred in DMF (5 mL) and sodium hydride (60% in mineral oil) (21 mg, 0.52 mmol) was added. Stirred for 20 minutes, after which 2-bromopropane (0.050 mL, 0.53 mmol) was added and the mixture was stirred for another 16 hours. The reaction was quenched with 20 mL of water and extracted with EtOAc (20 mL). The organic layer was washed with sat. aq. NH_4Cl (2x 10 mL), brine (10 mL) and dried over Na_2SO_4 after which volatiles were evaporated. The resulting crude was purified over SiO_2 using a gradient of 10% EtOAc in heptane towards 60% EtOAc in heptane yielding 140 mg (0.39 mmol,82%) of the title compound as a white solid. ^{1}H-NMR: (500 MHz, Chloroform-*d*) δ 7.61 (dd, J = 8.8, 8.1 Hz, 1H), 6.75 (dd, J = 8.9, 1.4 Hz, 1H), 4.49 (hept, J = 6.7 Hz, 1H), 3.94 (s, 3H), 1.37 - 1.29 (m, 12H). ^{3}C-NMR: (126 MHz, Chloroform-d) δ 177.6, 158.8 (d, *J* = 4 Hz), 158.4 (d, *J* = 4 Hz), 157.4 (d, *J* = 251 Hz), 128.9 (d, *J* = 5 Hz), 113.4 (d, *J* = 16 Hz), 107.2 (d, *J* = 2 Hz), 100.3 (d, *J* = 22 Hz), 56.8, 49.9, 45.3, 21.4, 21.3, 20.8. LC-MS (ESI) *m/z* found: 357 $[M+H]^+$; retention time: 5.04 minutes. HRMS-ESI $[M+H]^+$ calculated for $C_{15}H_{19}BrFN_2O_2$: 357.0608, found: 357.0581.

3-(5-bromo-2-fluoro-4-methoxyphenyl)-1-isopropyl-4,4-dimethyl-1H-pyrazol-5(4H)-one (**54**)

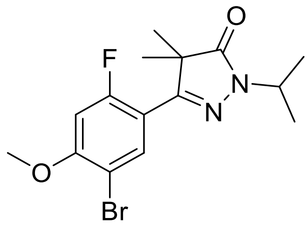

Dihydropyrazolone **42** (500 mg, 1.59 mmol) was stirred in DMF (5 mL) and sodium hydride (60% in mineral oil) (67 mg, 1.67 mmol) was added. Stirred for 10 minutes, after which 2-bromopropane (0.15 mL, 1.59 mmol) was added and the reaction was stirred for 18 hours. The reaction was quenched with water, extracted with EtOAc (40 mL),

which was washed with water (2x 20 mL) and brine (20 mL). The resulting crude was purified over SiO_2 using a gradient of 80% c-hexane in EtOAc towards EtOAc to yield 480 mg (1.3 mmol, 85%) of the title compound as a white solid. ^{1}H NMR (600 MHz, Chloroform-d) δ 7.98 (d, *J* = 7.8 Hz, 1H), 6.67 (d, *J* = 12.6 Hz, 1H), 4.50 (hept, *J* = 6.7 Hz, 1H), 3.93 (s, 3H), 1.37 – 1.35 (m, 12H). ^{13}C NMR (151 MHz, Chloroform-d) δ 177.7, 159.8 (d, *J* = 253 Hz), 158.2 (d, *J* = 3 Hz), 157.9 (d, *J* = 10 Hz), 133.2, 113.0 (d, *J* = 16 Hz), 106.9 (d, *J* = 3 Hz), 100.7 (d, *J* = 27 Hz), 56.7, 49.7, 45.4, 21.2, 21.1, 20.8. LC-MS (ESI) *m*/*z* found: 357 $[M+H]^+$; retention time: 5.05 minutes. HRMS-ESI $[M+H]^+$ calculated for $C_{15}H_{19}BrFN_2O_2$: 357.0608, found: 357.0592.

3-(3-bromo-4-methoxy-5-methylphenyl)-1-isopropyl-4,4-dimethyl-1H-pyrazol-5(4H)-one (**55**)

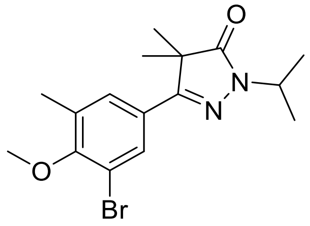

Dihydropyrazolone **43** (500 mg, 1.61 mmol) was stirred in DMF (5 mL) and sodium hydride (60% in mineral oil) (67.5 mg, 1.69 mmol) was added. Stirred for 10 minutes, after which 2-bromopropane (0.151 mL, 1.61 mmol) was added and the mixture was stirred for another 16 hours. The reaction was quenched with 20 mL of water, extracted with EtOAc (40 mL), which was washed with water (2x 20 mL) and brine (20 mL). The resulting crude was purified over SiO_2 using a gradient of 80% c-hexane in EtOAc towards EtOAc to yield 410 mg (1.2 mmol, 72%) of the title compound as a white solid.^{1}H NMR (600 MHz, Chloroform-d) δ 7.82 (d, *J* = 1.8 Hz, 1H), 7.55 (d, 1H), 4.50 (hept, *J* = 6.7 Hz, 1H), 3.84 (s, 3H), 2.37 (s, 3H), 1.44 (s, 6H), 1.36 (d, *J* = 6.7 Hz, 6H). ^{13}C NMR (151 MHz, Chloroform-d) δ 177.7, 160.1, 156.6, 133.5, 128.9, 128.4, 128.1, 117.8, 60.3, 48.7, 45.4, 22.5, 20.8, 16.8. LC-MS (ESI) *m*/*z* found: 353 $[M+H]^+$; retention time: 5.35 minutes. HRMS-ESI $[M+H]^+$ calculated for $C_{16}H_{22}BrN_2O_2$: 353.0859, found: 353.0849.

3-(5-bromo-4-methoxy-2-methylphenyl)-1-isopropyl-4,4-dimethyl-1H-pyrazol-5(4H)-one (**56**)

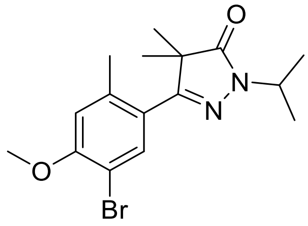

Dihydropyrazolone **44** (500 mg, 1.61 mmol) was stirred in DMF (5 mL) and sodium hydride (60% in mineral oil) (67.5 mg, 1.687 mmol) was added. Stirred for 10 minutes, after which 2-bromopropane (0.15 mL, 1.61 mmol) was added and the reaction was stirred for 18 hours. The reaction was quenched with water, extracted with EtOAc (40 mL), which was washed with water (2x 20 mL) and brine (20 mL). The resulting crude was purified over SiO_2 using a gradient of 80% c-hexane in EtOAc towards EtOAc to yield 410 mg (1.1 mmol, 72%) of the target compound as a white solid. ^{1}H NMR (600 MHz, Chloroform-d) δ 7.48 (s, 1H), 6.81 (s, 1H), 4.52 (hept, J = 6.7 Hz, 1H), 3.92 (s, 3H), 2.38 (s, 3H), 1.34 (d, J = 6.7 Hz, 6H), 1.32 (s, 6H). ^{13}C NMR (151 MHz, Chloroform-d) δ 177.0, 162.1, 156.0, 139.3, 132.6, 124.6, 114.7, 108.2, 56.3, 50.5, 45.0, 21.6, 21.5, 20.8. LC-MS (ESI) *m/z* found: 369 $[M+H]^+$; retention time: 5.07 minutes. HRMS-ESI $[M+H]^+$ calculated for $C_{16}H_{22}BrN_2O_3$: 369.080, found: 369.0803.

1-isopropyl-3-(3-methoxy-5-(pyridin-3-yl)phenyl)-4,4-dimethyl-1H-pyrazol-5(4H)-one (**57**)

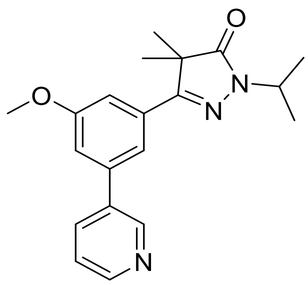

Dihydropyrazolone **45** (150 mg, 0.44 mmol) and pyridin-3-ylboronic acid (82 mg, 0.66 mmol) were added to a microwave tube with a stirring bean, subsequently DME (3 ml) and 1M sodium carbonate (1.3 mL, 1.3 mmol) were added and the mixture was degassed for 5 minutes with N_2. After addition of $PdCl_2(dppf).CH_2Cl_2$ (36 mg, 44 µmol) the

mixture was degassed for another 2 minutes and the vessel was sealed and heated for 1 hour at 120°C. The mixture was diluted with MTBE (25 mL), filtered over Celite and the organic layer was washed with 1M aqueous Na_2CO_3 (2x 25 mL) and brine (25 mL). The organic layer was dried over Na_2SO_4, solids were filtered off and the residue was concentrated *in vacuo*. Remaining crude was purified over SiO_2 using 20% EtOAc in n-heptane towards 80% EtOAc in n-heptane to yield 112 mg (0.33 mmol, 75%) of the title compound as white solid. ^{1}H-NMR: (500 MHz, Chloroform-*d*) δ 8.85 (s, 1H), 8.64 – 8.57 (m, 1H), 7.88 (d, *J* = 7.7 Hz, 1H), 7.57 (s, 1H), 7.39 (dd, *J* = 7.3, 5.1 Hz, 1H), 7.36 (s, 1H), 7.10 (s, 1H), 4.51 (hept, *J* = 6.6 Hz, 1H), 3.90 (s, 3H), 1.48 (s, 6H), 1.36 (d, *J* = 6.7 Hz, 6H). ^{13}C-NMR: (126 MHz, Chloroform-d) δ 177.9, 161.0, 160.3, 148.8, 148.1, 139.8, 136.4, 134.5, 123.9, 117.6, 114.0, 111.4, 55.6, 48.9, 45.4, 22.7, 20.8. LC-MS (ESI) *m/z* found: 338 $[M+H]^+$; retention time: 4.41 minutes. HRMS-ESI $[M+H]^+$ calculated for $C_{20}H_{24}N_3O_2$: 338.1863, found 338.1850.

3-(3,4-dimethoxy-5-(pyridin-3-yl)phenyl)-1-isopropyl-4,4-dimethyl-1H-pyrazol-5(4H)-one (**58**)

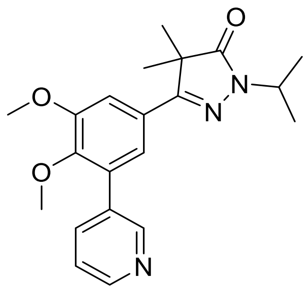

Dihydropyrazolone **46** (100 mg, 0.28 mmol) and pyridin-3-ylboronic acid (50 mg, 0.42 mmol) were charged to a microwave vial after which DME (3.5 mL) and 1M Na_2CO_3 (1.1 mL, 1.1 mmol) were added. The mixture was degassed with N_2 for 5 m after which Pd(dppf)Cl_2 (23 mg, 0.03 mmol) was added. The reaction was heated in the microwave for 1 h at 120°C. The reaction mixture was diluted with MTBE and filtered over Celite. The residue was washed with saturated $NaHCO_3$ (2x) and brine (1x). The organic phase was dried over Na_2SO_4, filtered and concentrated *in vacuo* to be further purified over SiO_2 using a

gradient of 40% EtOAc in heptane towards 80% EtOAc and subsequently recrystallized from i-PrOH:H_2O to yield 62 mg (0.18 mmol, 62%) of the title compound. ^{1}H NMR (500 MHz, Chloroform-d) δ 8.81 – 8.77 (m, 1H), 8.62 (d, *J* = 4.1 Hz, 1H), 7.93 (d, *J* = 7.9 Hz, 1H), 7.49 (d, *J* = 1.7 Hz, 1H), 7.42 (dd, *J* = 7.7, 5.0 Hz, 1H), 7.31 (d, *J* = 1.9 Hz, 1H), 4.52 (hept, *J* = 6.7 Hz, 1H), 3.99 (s, 3H), 3.67 (s, 3H), 1.48 (s, 6H), 1.38 (d, *J* = 6.7 Hz, 6H). ^{13}C NMR (126 MHz, Chloroform-d) δ 177.8, 160.8, 153.4, 149.3, 148.1, 148.0, 137.2, 133.8, 132.1, 127.6, 123.3, 120.3, 110.1, 60.9, 56.1, 48.8, 45.4, 22.8, 20.8. LC-MS (ESI) *m/z* found: 368 $[M+H]^+$; retention time: 3.64 minutes. HRMS-ESI $[M+H]^+$ calculated for $C_{21}H_{26}N_3O_3$: 368.1969, found: 368.1955.

5-(3-bromo-4-methoxy-5-(pyridin-3-yl)phenyl)-2-isopropyl-4,4-dimethyl-2,4-dihydro-3H-pyrazol-3-one (**60**)

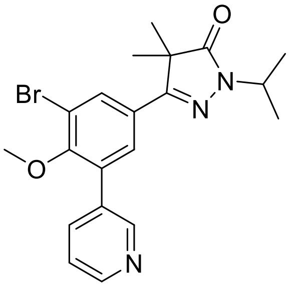

Dihydropyrazolone **48** (256 mg, 0.61 mmol) and pyridin-3-ylboronic acid (83 mg, 0.67 mmol) were charged to a microwave vial after which DME (4 mL) and 1M Na_2CO_3 (1.8 mL, 1.8 mmol) were added. The mixture was degassed with N_2 for 5 m after which Pd(dppf)Cl_2 (50 mg, 0.06 mmol) was added. The reaction was heated in the microwave for 1 h at 120°C. The reaction mixture was diluted with MTBE and filtered over Celite. The residue was washed with saturated $NaHCO_3$ (2x) and brine (1x). The organic phase was dried over Na_2SO_4, filtered and concentrated *in vacuo* to be further purified over SiO_2 using a gradient of 40% EtOAc in heptane towards 2% MeOH in EtOAc to yield 24 mg (0.06 mmol, 9%) of the title compound as a white solid.^{1}H-NMR: (600 MHz, Chloroform-*d*) δ 8.81 (s, 1H), 8.65 (dd, *J* = 4.8, 1.5 Hz, 1H), 8.02 (dd, *J* = 2.2, 0.9 Hz, 1H), 7.95 (dd, *J* = 7.8, 2.1 Hz, 1H), 7.72 (dd, *J* = 2.2, 1.0 Hz, 1H), 7.44 – 7.38 (m, 1H), 4.50 (hept, *J* = 6.7 Hz, 1H), 3.51 (s, 3H),

1.47 (s, 6H), 1.35 (d, *J* = 6.7 Hz, 6H). ^{13}C-NMR: (151 MHz, Chloroform-d) 177.6, 159.4, 155.6, 149.4, 149.0, 136.7, 133.5, 133.1, 131.0, 129.0, 127.6, 123.4, 118.8, 60.8, 48.7, 45.4, 22.5, 20.8. LC-MS (ESI) *m*/*z* found: 416 $[M+H]^+$; retention time: 4.50 minutes. HRMS-ESI $[M+H]^+$ calculated for $C_{20}H_{23}BrN_3O_2$: 308.1757, found 308.1757.

1-isopropyl-4,4-dimethyl-3-(3-(pyridin-3-yl)phenyl)-1H-pyrazol-5(4H)-one (**61**)

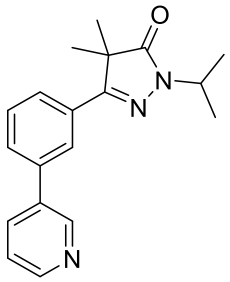

Dihydropyrazolone **49** (200 mg, 0.65 mmol) and pyridin-3-ylboronic acid (111 mg, 0.91 mmol) were added to a microwave tube with a stirring bean, subsequently DME (3 ml) and 1M sodium carbonate (2.5 mL, 2.5 mmol) were added and the mixture was degassed for 5 minutes with N_2. After addition of $PdCl_2(dppf).CH_2Cl_2$ (53 mg, 65 µmol) the mixture was degassed for another 2 minutes and the vessel was sealed and heated for 1 hour at 120°C. The mixture was diluted with MTBE (25 mL), filtered over Celite and the organic layer was washed with 1M aqueous Na_2CO_3 (2x 25 mL) and brine (25 mL). The organic layer was dried over Na_2SO_4, solids were filtered off and the residue was concentrated *in vacuo*. Remaining crude was purified over SiO_2 using 30% EtOAc in n-heptane towards 90% EtOAc in n-heptane to yield 63 mg (0.20 mmol, 6% over 4 steps) of the title compound as white solid. ^{1}H-NMR: (600 MHz, Chloroform-*d*) δ 8.85 (s, 1H), 8.60 (d, 1H), 8.03 (s, 1H), 7.88 (d, *J* = 7.8 Hz, 1H), 7.79 (d, *J* = 7.7 Hz, 1H), 7.58 (d, *J* = 7.6 Hz, 1H), 7.51 (t, *J* = 7.7 Hz, 1H), 7.38 (dd, *J* = 7.4, 5.0 Hz, 1H), 4.51 (hept, *J* = 6.5 Hz, 1H), 1.49 (s, 6H), 1.36 (d, *J* = 6.8 Hz, 6H). ^{13}C-NMR: (151 MHz, Chloroform-d) δ 177.8, 161.1, 148.9, 148.3, 138.5, 136.2, 134.4, 132.1, 129.5, 128.4, 125.7, 124.9, 123.7, 48.8, 45.4, 22.6, 20.8. LC-MS (ESI) *m*/*z* found: 308 $[M+H]^+$; retention time: 3.68 minutes. HRMS-ESI $[M+H]^+$ calculated for $C_{19}H_{22}N_3O$: 308.1757, found 308.1757.

3-(4-fluoro-3-(pyridin-3-yl)phenyl)-1-isopropyl-4,4-dimethyl-1H-pyrazol-5(4H)-one (**62**)

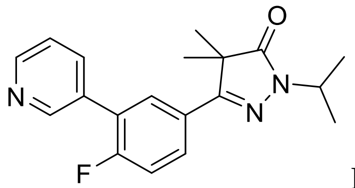

Dihydropyrazolone **50** (200 mg, 0.61 mmol) and pyridin-3-ylboronic acid (98 mg, 0.80 mmol) were added to a microwave tube with a stirring bean, subsequently DME (4 ml) and 1M sodium carbonate (1.8 mL, 1.8 mmol) were added and the mixture was degassed for 5 minutes with N_2. After addition of $PdCl_2(dppf).CH_2Cl_2$ (50 mg, 61 µmol) the mixture was degassed for another 2 minutes and the vessel was sealed and heated for 1 hour at 120°C. The mixture was diluted with MTBE (25 mL), filtered over Celite and the organic layer was washed with 1M aqueous Na_2CO_3 (2x 25 mL) and brine (25 mL). The organic layer was dried over Na_2SO_4, solids were filtered off and the residue was concentrated *in vacuo*. Remaining crude was purified over SiO_2 using 50% EtOAc in n-heptane towards 100% EtOAc to yield 154 mg (0.47 mmol, 77%) of the title compound as white solid. ^{1}H-NMR: (500 MHz, Chloroform-*d*) δ 8.83 (s, 1H), 8.69 – 8.64 (m, 1H), 7.96 – 7.88 (m, 2H), 7.81 (ddd, *J* = 8.5, 4.7, 2.3 Hz, 1H), 7.43 (dd, *J* = 7.8, 4.9 Hz, 1H), 7.29 – 7.22 (m, 1H), 4.52 (hept, *J* = 6.7 Hz, 1H), 1.49 (s, 6H), 1.37 (d, *J* = 6.7 Hz, 6H). ^{13}C-NMR: (126 MHz, Chloroform-d) δ 177.7, 160.5 (d, 251 Hz), 160.2 (d, 1 Hz), 149.6 (d, 3 Hz), 149.2, 136.3 (d, 3 Hz), 131.1, 128.5 (d, 4 Hz), 128.2 (d, 4 Hz), 127.7 (d, 9 Hz), 126.3 (d, 15 Hz), 123.4, 116.7 (d, 23.1 Hz), 48.7, 45.4, 22.6, 20.8. LC-MS (ESI) *m/z* found: 326 $[M+H]^+$; retention time: 3.99 minutes. HRMS-ESI $[M+H]^+$ calculated for $C_{19}H_{21}FN_3O$: 326.1663, found 326.1672.

3-(4-chloro-3-(pyridin-3-yl)phenyl)-1-isopropyl-4,4-dimethyl-1H-pyrazol-5(4H)-one (**63**)

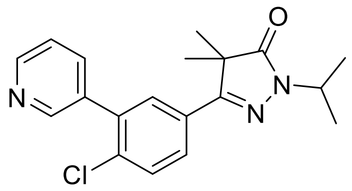

Dihydropyrazolone **51** (200 mg, 0.58 mmol) and pyridin-3-ylboronic acid (93 mg, 0.76 mmol) were added to a microwave tube with a stirring bean, subsequently DME (4 ml) and 1M sodium carbonate (1.8 mL, 1.8 mmol) were added and the mixture was degassed for 5 minutes with N_2. After addition of $PdCl_2$(dppf).CH_2Cl_2 (48 mg, 58 µmol) the mixture was degassed for another 2 minutes and the vessel was sealed and heated for 1 hour at 120°C. The mixture was diluted with MTBE (25 mL), filtered over Celite and the organic layer was washed with 1M aqueous Na_2CO_3 (2x 25 mL) and brine (25 mL). The organic layer was dried over Na_2SO_4, solids were filtered off and the residue was concentrated *in vacuo*. Remaining crude was purified over SiO_2 using 50% EtOAc in n-heptane towards 100% EtOAc to yield 128 mg (0.37 mmol, 64%) of the title compound as white solid. ^{1}H-NMR: (500 MHz, Chloroform-*d*) δ 8.73 – 8.68 (m, 1H), 8.68 – 8.62 (m, 1H), 7.85 – 7.79 (m, 2H), 7.74 (dd, *J* = 8.4, 2.2 Hz, 1H), 7.54 (d, *J* = 8.4 Hz, 1H), 7.41 (dd, *J* = 7.8, 4.9 Hz, 1H), 4.50 (hept, *J* = 6.7 Hz, 1H), 1.47 (s, 6H), 1.35 (d, *J* = 6.7 Hz, 6H). ^{13}C-NMR: (126 MHz, Chloroform-d) δ 177.7, 160.0, 149.9, 149.1, 137.5, 136.9, 134.7, 134.1, 130.5, 130.3, 128.7, 126.8, 123.0, 48.7, 45.4, 22.5, 20.8. LC-MS (ESI) *m*/*z* found: 342 $[M+H]^+$; retention time: 4.29 minutes. HRMS-ESI $[M+H]^+$ calculated for $C_{19}H_{21}ClN_3O$: 342.1368, found 342.1374.

1-isopropyl-4,4-dimethyl-3-(4-methyl-3-(pyridin-3-yl)phenyl)-1H-pyrazol-5(4H)-one (**64**)

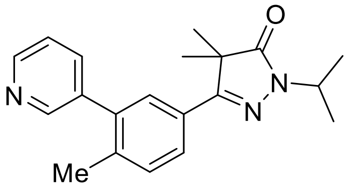

Dihydropyrazolone **52** (200 mg, 0.62 mmol) and pyridin-3-ylboronic acid (99 mg, 0.80 mmol) were added to a microwave tube with a stirring bean, subsequently DME (4 ml) and 1M sodium carbonate (1.9 mL, 1.9 mmol) were added and the mixture was degassed for 5 minutes with N_2. After addition of $PdCl_2(dppf).CH_2Cl_2$ (51 mg, 62 μmol) the mixture was degassed for another 2 minutes and the vessel was sealed and heated for 1 hour at 120°C. The mixture was diluted with MTBE (25 mL), filtered over Celite and the organic layer was washed with 1M aqueous Na_2CO_3 (2x 25 mL) and brine (25 mL). The organic layer was dried over Na_2SO_4, solids were filtered off and the residue was concentrated *in vacuo*. Remaining crude was purified over SiO_2 using 50% EtOAc in n-heptane towards 100% EtOAc to yield 175 mg (0.54 mmol, 88%) of the title compound as white solid. ^{1}H-NMR: (500 MHz, Chloroform-*d*) δ 8.66 – 8.58 (m, 2H), 7.74 – 7.64 (m, 3H), 7.38 (dd, *J* = 7.7, 4.9 Hz, 1H), 7.33 (d, *J* = 7.9 Hz, 1H), 4.49 (hept, *J* = 6.7 Hz, 1H), 2.28 (s, 3H), 1.46 (s, 6H), 1.34 (d, *J* = 6.7 Hz, 6H). ^{13}C-NMR: (126 MHz, Chloroform-d) δ 177.8, 161.2, 149.8, 148.5, 138.7, 137.6, 136.9, 136.5, 130.9, 129.2, 127.5, 125.7, 123.2, 48.8, 45.3, 22.6, 20.8, 20.4. LC-MS (ESI) *m*/*z* found: 322 $[M+H]^+$; retention time: 3.78 minutes. HRMS-ESI $[M+H]^+$ calculated for $C_{20}H_{24}N_3O$: 322.1914, found 322.1916.

5-(2-fluoro-4-methoxy-3-(pyridin-3-yl)phenyl)-2-isopropyl-4,4-dimethyl-2,4-dihydro-3H-pyrazol-3-one (**65**)

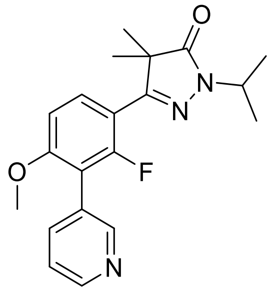

Dihydropyrazolone **53** (140 mg, 0.39 mmol) and pyridin-3-ylboronic acid (58 mg, 0.47 mmol) were charged to a microwave vial after which DME (3 mL) and 1M Na_2CO_3 (1.2 mL, 1.2 mmol) were added. The mixture was degassed with N_2 for 5 m after which Pd(dppf)Cl_2 (32 mg, 0.04 mmol) was added. The reaction was heated in the microwave for 1 h at 120°C. The reaction mixture was diluted with MTBE and filtered over Celite. The residue was washed with saturated $NaHCO_3$ (2x) and brine (1x). The organic phase was dried over Na_2SO_4, filtered and concentrated *in vacuo* to be further purified over SiO_2 using a gradient of 20% EtOAc in heptane towards 80% EtOAc and subsequently recrystallized from i-PrOH:H_2O to yield 90 mg (0.25 mmol, 65%) of the title compound.^{1}H-NMR: (500 MHz, Chloroform-*d*) δ 8.64 (s, 1H), 8.59 (d, *J* = 4.8 Hz, 1H), 7.73 (dt, *J* = 7.7, 1.6 Hz, 1H), 7.67 (t, *J* = 8.5 Hz, 1H), 7.37 (dd, *J* = 7.9, 4.7 Hz, 1H), 6.86 (dd, *J* = 8.8, 0.9 Hz, 1H), 4.50 (hept, *J* = 6.8 Hz, 1H), 3.82 (s, 3H), 1.38 – 1.30 (m, 12H). ^{13}C-NMR: (126 MHz, Chloroform-*d*) 177.6, 159.6 (d, 3 Hz), 158.8 (d, 7 Hz), 157.7 (d, 250 Hz), 151.2, 148.7, 138.1, 130.3 (d, 6 Hz), 127.2, 123.0, 115.8 (d, 20 Hz), 112.9 (d, 15 Hz), 107.1 (d, 3 Hz), 56.2, 50.0, 45.2, 21.4, 20.8. LC-MS (ESI) *m/z* found: 356 $[M+H]^+$; retention time: 3.67 minutes. HRMS-ESI $[M+H]^+$ calculated for $C_{20}H_{24}FN_3O_2$: 356.1769, found: 356.1772.

3-(2-fluoro-4-methoxy-5-(pyridin-3-yl)phenyl)-1-isopropyl-4,4-dimethyl-1H-pyrazol-5(4H)-one (**66**)

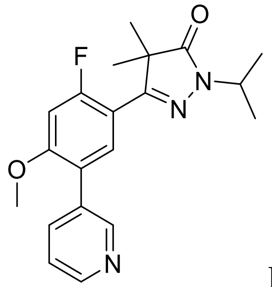

Dihydropyrazolone **54** (100 mg, 0.28 mmol) and pyridin-3-ylboronic acid (52 mg, 0.42 mmol) were charged to a microwave vial after which DME (3.5 mL) and 1M Na_2CO_3 (1.1 mL, 1.1 mmol) were added. The mixture was degassed with N_2 for 5 m after which Pd(dppf)Cl_2 (23 mg, 0.03 mmol) was added. The reaction was heated in the microwave for 1 h at 120°C. The reaction mixture was diluted with MTBE and filtered over Celite. The residue was washed with saturated $NaHCO_3$ (2x) and brine (1x). The organic phase was dried over Na_2SO_4, filtered and concentrated *in vacuo* to be further purified over SiO_2 using a gradient of 20% EtOAc in heptane towards 80% EtOAc and subsequently recrystallized from i-PrOH:H_2O to yield 64 mg (0.18 mmol, 64%) of the title compound. ^{1}H NMR (600 MHz, Chloroform-*d*) δ 8.75 (s, 1H), 8.59 (d, *J* = 4.7 Hz, 1H), 7.85 (d, *J* = 7.9 Hz, 1H), 7.74 (d, *J* = 8.3 Hz, 1H), 7.38 (dd, *J* = 7.7, 4.9 Hz, 1H), 6.77 (d, *J* = 12.9 Hz, 1H), 4.51 (hept, *J* = 6.7 Hz, 1H), 3.86 (s, 3H), 1.38 (s, 6H), 1.35 (d, *J* = 6.7 Hz, 6H). ^{13}C NMR (151 MHz, Chloroform-d) δ 177.8, 160.7 (d, *J* = 253 Hz), 159.1 (d, *J* = 3 Hz), 158.8 (d, *J* = 10 Hz), 149.8, 148.0, 137.2, 133.0, 131.3 (d, *J* = 6 Hz), 123.7, 123.2, 112.0 (d, *J* = 14 Hz), 100.1 (d, *J* = 29 Hz), 56.1, 49.8, 45.4, 21.2, 21.2, 20.8. LC-MS (ESI) *m*/*z* found: 356 $[M+H]^+$; retention time: 3.59 minutes. HRMS-ESI $[M+H]^+$ calculated for $C_{20}H_{23}FN_3O_2$: 356.1769, found: 356.1763.

1-isopropyl-3-(4-methoxy-3-methyl-5-(pyridin-3-yl)phenyl)-4,4-dimethyl-1H-pyrazol-5(4H)-one (**67**)

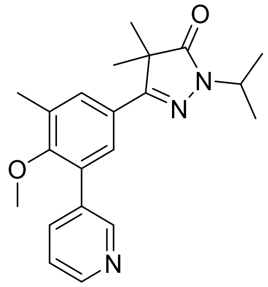

Dihydropyrazolone **55** (100 mg, 0.28 mmol) and pyridin-3-ylboronic acid (52 mg, 0.43 mmol) were charged to a microwave vial after which DME (3.5 mL) and 1M Na_2CO_3 (1.1 mL, 1.1 mmol) were added. The mixture was degassed with N_2 for 5 m after which Pd(dppf)Cl_2 (23 mg, 0.03 mmol) was added. The reaction was heated in the microwave for 1 h at 120°C. The reaction mixture was diluted with MTBE and filtered over Celite. The residue was washed with saturated $NaHCO_3$ (2x) and brine (1x). The organic phase was dried over Na_2SO_4, filtered and concentrated *in vacuo* to be further purified over SiO_2 using a gradient of 40% EtOAc in heptane towards 80% EtOAc and subsequently recrystallized from i-PrOH:H_2O to yield 52 mg (0.15 mmol, 52%) of the title compound. ^{1}H NMR (600 MHz, Chloroform-d) δ 8.84 (s, 1H), 8.64 (d, *J* = 3.9 Hz, 1H), 8.02 (d, *J* = 7.6 Hz, 1H), 7.67 (d, *J* = 2.2 Hz, 1H), 7.63 (d, *J* = 2.2 Hz, 1H), 7.48 – 7.42 (m, 1H), 4.51 (hept, *J* = 6.6 Hz, 1H), 3.42 (s, 3H), 2.41 (s, 3H), 1.48 (s, 6H), 1.37 (d, *J* = 6.7 Hz, 6H). ^{13}C NMR (151 MHz, Chloroform-d) δ 177.8, 160.9, 157.3, 148.8, 147.6, 137.4, 134.4, 132.6, 131.4, 129.3, 127.6, 126.4, 123.6, 60.3, 48.8, 45.3, 22.7, 20.8, 16.5. LC-MS (ESI) *m/z* found: 352 [M+H]$^+$; retention time: 3.88 minutes. HRMS-ESI [M+H]$^+$ calculated for $C_{21}H_{26}N_3O_2$: 352.2020, found: 352.2007.

1-isopropyl-3-(4-methoxy-2-methyl-5-(pyridin-3-yl)phenyl)-4,4-dimethyl-1H-pyrazol-5(4H)-one (**68**)

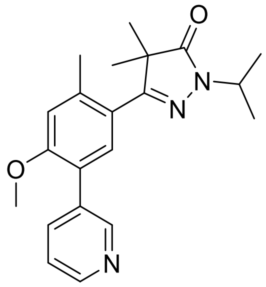

Dihydropyrazolone **56** (100 mg, 0.28 mmol) and pyridin-3-ylboronic acid (0.52 mg, 0.42 mmol) were charged to a microwave vial after which DME (3.5 mL) and 1M

Na_2CO_3 (1.1 mL, 1.1 mmol) were added. The mixture was degassed with N_2 for 5 m after which Pd(dppf)Cl_2 (23 mg, 0.03 mmol) was added. The reaction was heated in the microwave for 1 h at 120°C. The reaction mixture was diluted with MTBE and filtered over Celite. The residue was washed with saturated $NaHCO_3$ (2x) and brine (1x). The organic phase was dried over Na_2SO_4, filtered and concentrated *in vacuo* to be further purified over SiO_2 using a gradient of 40% EtOAc in heptane towards 80% EtOAc and subsequently recrystallized from i-PrOH:H_2O to yield 62 mg (0.18 mmol, 62%) of the title compound. ^{1}H NMR (600 MHz, Chloroform-d) δ 8.77 (s, 1H), 8.59 (s, 1H), 7.88 (d, J = 7.7 Hz, 1H), 7.42 – 7.36 (m, 1H), 7.30 (s, 1H), 6.95 (s, 1H), 4.56 (hept, J = 6.7 Hz, 1H), 3.88 (s, 3H), 2.50 (s, 3H), 1.38 (d, J = 6.7 Hz, 6H), 1.37 (s, 6H). ^{13}C NMR (151 MHz, Chloroform-d) δ 177.1, 163.0, 156.7, 149.7, 147.7, 140.3, 137.0, 133.7, 130.2, 124.2, 123.9, 123.2, 114.3, 55.7, 50.6, 45.0, 21.8, 21.6, 20.8. LC-MS (ESI) *m/z* found: 352 $[M+H]^+$; retention time: 3.56 minutes. HRMS-ESI $[M+H]^+$ calculated for $C_{21}H_{26}N_3O_2$: 352.2020, found: 352.2013.

methyl 3-(4-bromo-3-chlorophenyl)-2,2-dimethyl-3-oxopropanoate (**70**)

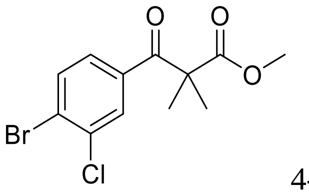

4-bromo-3-chlorobenzoic acid (5.0 g, 21.2 mmol) was dissolved in DCM (20 mL) and oxalyl chloride (2.4 ml, 27.6 mmol) was added followed by a drop of DMF. This mixture was stirred for 3 h, after which volatiles were evaporated and the mixture was redissolved in ~30 mL THF. In a different flask methyl isobutyrate (3.2 ml, 27.6 mmol) was dissolved in THF (40 mL) and the mixture was cooled down to -78 °C after which 2M LDA (in toluene, THF, heptane) (11.7 ml, 23.4 mmol) was added dropwise, while maintaining -78 °C. Upon completion of addition of the LDA the mixture was stirred for 15 minutes followed by the dropwise addition of the chlorinated 4-bromo-3-chlorobenzoic acid in THF, maintaining the temperate at -78 °C. Upon completion of the addition the mixture was allowed to warm up

to room temperature and the reaction was quenched by the addition of 200 mL water. The aqueous layer was extracted with Et_2O (250 mL) after which the organic layer was washed with brine (200 mL). The resulting crude was used in the next step without further purification.

3-(4-bromo-3-chlorophenyl)-4,4-dimethyl-1H-pyrazol-5(4H)-one (**71**)

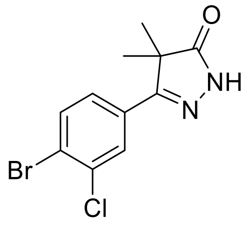

Crude keto-ester **69** (6.7 g, 21 mmol) was dissolved in ethanol (15 mL) and hydrazine hydrate (60-65%) (10 mL, 210 mmol) was added. The reaction was stirred for 16 hours after which volatiles were evaporated and the resulting crude was suspended in water and extracted with EtOAc (2x 100 mL). The combined organic layers were washed with water (2x 150 mL) and brine (150 mL), dried over Na_2SO_4 and solids were filtered off. After evaporation of volatiles the resulting crude was purified over SiO_2 using a gradient of 20% EtOAc towards 50% EtOAc yielding 1.5 g (5.0 mmol, 24% over two steps) of the title compound as a white solid. ^{1}H-NMR: (500 MHz, Chloroform-*d*) δ 10.13 (s, 1H), 7.87 (d, *J* = 2.1 Hz, 1H), 7.65 (d, *J* = 8.4 Hz, 1H), 7.52 (dd, *J* = 8.4, 2.1 Hz, 1H), 1.49 (s, 6H). ^{13}C-NMR: (126 MHz, Chloroform-d) δ 181.1, 168.4, 139.3, 134.8, 133.9, 127.7, 125.1, 122.8, 20.6.LC-MS (ESI) *m*/*z* found: 318 $[M+H]^+$; retention time: 5.26 minutes.

3-(4-bromo-3-chlorophenyl)-1-isopropyl-4,4-dimethyl-1H-pyrazol-5(4H)-one (**72**)

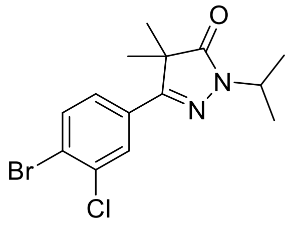

Dihydropyrazolone **70** (1.0 g, 3.3 mmol) was dissolved in DMF (7 mL) and sodium hydride (60% in mineral oil) (0.15 g, 3.7 mmol) was added and the mixture was stirred for 30 minutes after which 2-bromopropane (0.35 mL, 3.7 mmol) was added. The

mixture was stirred for 16 hours at rt after which the reaction was quenched with 20 mL of water. The aqueous layer was extracted with MTBE (25 mL) and this organic layer was then washed with water (2x 25 mL) and brine (25 mL). After drying over Na_2SO_4 and filtration of solids was followed by evaporation of volatiles, which gave a crude which was purified over SiO_2 using a gradient of 10% EtOAc in n-heptane towards 40% EtOAc in n-heptane. Evaporation gave 850 mg (2.5 mmol, 75%) of the title compound as a white solid. LC-MS (ESI) *m/z* found: 265 $[M+H]^+$; retention time: 5.71 minutes, purity: 94%.

2-chloro-4-(1-isopropyl-4,4-dimethyl-5-oxo-4,5-dihydro-1H-pyrazol-3-yl)benzonitrile (**73**)

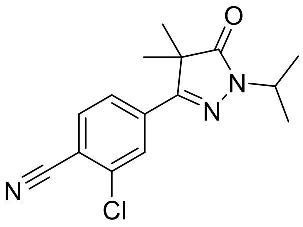

Dihydropyrazolone **72** (700 mg, 2.0 mmol) was dissolved in DMF (10 mL) and copper cyanide (401 mg, 4.5 mmol) was added. The mixture was stirred for 8 hours at 160oC, after which the reaction was quenched in 1M NaOH (20 mL) and extracted with MTBE (20 mL). The organic layer was washed with 1M aqueous Na_2CO_3 (20 mL) and brine (20 mL) after which it was dried over Na_2SO_4. After filtering off the solids, remaining volatiles were evaporated and the resulting crude was purified over SiO_2 using a gradient of 10% EtOAc in n-heptane towards 45% EtOAc in n-heptane to yield 350 mg (1.2 mmol, 60%) of the title compound as a white solid. ^{1}H-NMR: (500 MHz, Chloroform-*d*) δ 7.97 (d, *J* = 1.5 Hz, 1H), 7.76 (dd, *J* = 8.2, 1.6 Hz, 1H), 7.71 (d, *J* = 8.2 Hz, 1H), 4.53 (hept, *J* = 6.7 Hz, 1H), 1.47 (s, 6H), 1.37 (d, *J* = 6.7 Hz, 6H). ^{13}C-NMR: (126 MHz, Chloroform-d) δ 177.7, 158.2, 137.4, 136.4, 134.2, 127.0, 124.2, 115.7, 113.5, 48.3, 45.7, 22.4, 20.9. LC-MS (ESI) *m/z* found: 290 $[M+H]^+$; retention time: 4.97 minutes. HRMS-ESI $[M+H]^+$ calculated for $C_{15}H_{17}N_3O$: 290.1055, found 290.1041.

4-(1-isopropyl-4,4-dimethyl-5-oxo-4,5-dihydro-1H-pyrazol-3-yl)-2-(pyridin-3-yl)benzonitrile (**74**)

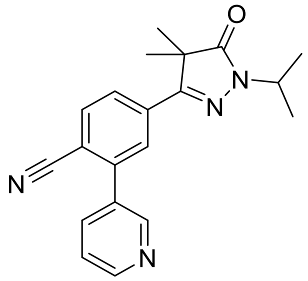

Dihydropyrazolone **73** (100 mg, 0.35 mmol) and pyridin-3-ylboronic acid (64 mg, 0.52 mmol) were added to a microwave tube with a stirring bean, subsequently DME (3 ml) and 1M sodium carbonate (1.0 mL, 1.0 mmol) were added and the mixture was degassed for 5 minutes with N_2. After addition of $PdCl_2$(dppf).CH_2Cl_2 (28 mg, 35 µmol) the mixture was degassed for another 2 minutes and the vessel was sealed and heated for 1 hour at 120°C. The mixture was diluted with MTBE (25 mL), filtered over Celite and the organic layer was washed with 1M aqueous Na_2CO_3 (2x 25 mL) and brine (25 mL). The organic layer was dried over Na_2SO_4, solids were filtered off and the residue was concentrated *in vacuo*. Remaining crude was purified over SiO_2 using 50% EtOAc in n-heptane towards 100% EtOAc to yield 70 mg (0.25 mmol, 61%) of the title compound as white solid. ^{1}H-NMR: (500 MHz, Chloroform-*d*) δ 8.80 (d, *J* = 2.4 Hz, 1H), 8.76 – 8.69 (m, 1H), 8.01 – 7.94 (m, 2H), 7.90 (dd, *J* = 8.2, 1.7 Hz, 1H), 7.84 (d, *J* = 8.2 Hz, 1H), 7.47 (dd, *J* = 7.9, 4.8 Hz, 1H), 4.52 (hept, *J* = 6.7 Hz, 1H), 1.49 (s, 6H), 1.36 (d, *J* = 6.7 Hz, 6H). ^{13}C-NMR: (126 MHz, Chloroform-d) δ 177.7, 159.0, 150.2, 149.2, 142.3, 136.1, 135.5, 134.3, 133.7, 127.2, 125.5, 123.5, 117.8, 111.9, 48.5, 45.7, 22.5, 20.9. LC-MS (ESI) *m/z* found: 333 $[M+H]^+$; retention time: 4.17 minutes. HRMS-ESI $[M+H]^+$ calculated for $C_{20}H_{21}N_4O$: 333.1710, found 333.1701.

ethyl 3-(3-bromo-4-methoxyphenyl)-3-oxopropanoate (**76**)

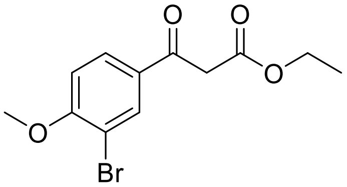

Benzoic acid **75** (5.6 g, 24.2 mmol) in THF (20 mL), di(1H-imidazol-1-yl) methanone (5.9 g, 36.4 mmol) was added. The reaction was stirred at 40°C for 2 hours. In a separate flask a suspension of potassium 3-ethoxy-3-oxopropanoate (4.1 g, 24.2 mmol) in acetonitrile (20 mL), triethylamine (10.0 mL, 71.5 mmol) and magnesium chloride (5.8 g, 60.6 mmol) was prepared and stirred for 15 minutes after which it was added to the other flask. This suspension was stirred for 3 hours at room temperature after which it was quenched with 200 mL water and extracted with EtOAc (250 mL). The organic layer was washed with water (2x 200 mL), brine (200 mL) and dried over Na_2SO_4.The resulting crude was purified over SiO_2 using a gradient of 10% EtOAc in n-heptane towards 40% EtOAc in n-heptane yielding 3.2 g (10.6 mmol, 44%) of the title compound as a white solid.^{1}H NMR (500 MHz, Chloroform-d) δ 8.14 (d, *J* = 2.2 Hz, 1H), 7.99 (dd, *J* = 8.7, 2.2 Hz, 1H), 7.25 (d, *J* = 8.7 Hz, 1H), 4.16 (s, 2H), 4.11 (q, *J* = 7.1 Hz, 2H), 3.95 (s, 3H), 1.17 (t, *J* = 7.1 Hz, 3H).^{13}C NMR (126 MHz, Chloroform-d) δ 191.6, 168.2, 160.0, 133.7, 130.8, 130.3, 112.9, 111.4, 61.1, 57.3, 45.8, 14.5. LC-MS (ESI) *m/z* found: no mass observed; retention time: 5.99 minutes. `

ethyl 1-(3-bromo-4-methoxybenzoyl)cyclopentanecarboxylate (**77**)

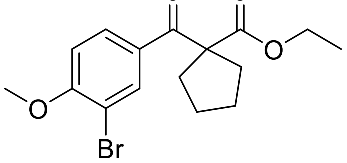

Keto-ester **76** (1.0 g, 3.3 mmol), 1,4-dibromobutane (0.60 mL, 4.9 mmol) and potassium carbonate (1.61 g, 11.6 mmol) were stirred in DMSO (10 mL) for 16 h. The reaction was quenched with water (30 mL) and extracted with MTBE (30 mL). The organic layer was washed with water (2x 20 mL) and brine (20 mL), dried over $MgSO_4$. After

evaporation of volatiles the remaining crude was purified over SiO_2 using a gradient of 80% heptane in EtOAc towards 50% EtOAc in heptane to yield 600 mg (1.69 mmol, 51%) of the title compound as an off white solid. ^{1}H NMR (500 MHz, Chloroform-d) δ 8.11 (d, *J* = 2.2 Hz, 1H), 7.77 (dd, *J* = 8.7, 2.2 Hz, 1H), 6.87 (d, *J* = 8.7 Hz, 1H), 4.06 (q, *J* = 7.1 Hz, 2H), 3.93 (s, 3H), 2.40 – 2.20 (m, 4H), 1.81 – 1.57 (m, 4H), 1.02 (t, *J* = 7.1 Hz, 3H). ^{13}C NMR (126 MHz, Chloroform-d) δ 193.5, 174.6, 159.1, 134.4, 129.8, 129.0, 111.8, 110.8, 63.4, 61.4, 56.4, 34.9, 26.2, 13.8. LC-MS (ESI) *m/z* found: no mass observed; retention time: 6.10 minutes.

methyl 1-(3-bromo-4-methoxybenzoyl)cyclopent-3-enecarboxylate (**78**)

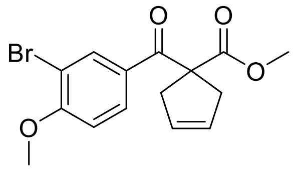

Benzoic acid **75** (1.0 g, 4.3 mmol) was suspended in DCM (10 mL) while cooling to 0°C. Subsequently oxalyl dichloride (0.48 mL, 5.6 mmol) and a few drops of DMF were added and the mixture was allowed to warm up to room temperature. The mixture was stirred for 2 h after which volatiles were evaporated. The remaining solids were re-dissolved in 10 mL of THF. In a separate flask methyl cyclopent-3-enecarboxylate (0.71 g, 5.6 mmol) was stirred in THF (10 mL) at -78°C and a 2M LDA (2.6 mL, 5.2 mmol) was added dropwise while maintaining -78°C. Upon full addition, the mixture was stirred for 45 m after which the acid chloride in THF was added dropwise, maintaining the temperate at -78°C. The reaction was allowed to warm up to room temperature after which it was quenched with water and extracted with diethyl ether. The organic phase was washed twice with sat. aq. Na_2CO_3 and once with brine. The organic layer was then dried with $MgSO_4$, filtered and evaporated to dryness. The crude was used in the next step without further purification. Crude ^{1}H NMR (500 MHz, Chloroform-d) δ 8.14 (d, *J* = 2.1 Hz, 1H), 7.76 (dd, *J* = 8.7, 2.1 Hz, 1H), 6.89 (d, *J* = 8.6 Hz, 1H), 5.61 (s, 2H), 3.96 (s, 3H), 3.63 (s, 3H), 3.27 – 3.06 (m, 4H).

methyl 4-(3-bromo-4-methoxybenzoyl)-1-methylpiperidine-4-carboxylate (**79**)

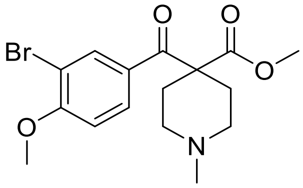

Benzoic acid **75** (1.0 g, 4.3 mmol) was suspended in DCM (10 mL) while cooling to 0°C. Subsequently oxalyl dichloride (0.48 mL, 5.6 mmol) and a few drops of DMF were added and the mixture was allowed to warm up to room temperature. The mixture was stirred for 2 h after which volatiles were evaporated. The remaining solids were re-dissolved in 10 mL of THF. In a separate flask methyl 1-methylpiperidine-4-carboxylate (0.80 mL, 5.6 mmol) was stirred in THF (10 mL) at -78°C and a 2M LDA (2.6 mL, 5.2 mmol) was added dropwise while maintaining -78°C. Upon full addition, the mixture was stirred for 45 m after which the acid chloride in THF was added dropwise, maintaining the temperate at -78°C. The reaction was allowed to warm up to room temperature after which it was quenched with water and extracted with diethyl ether. The organic phase was washed twice with sat. aq. Na_2CO_3 and once with brine. The organic layer was then dried with $MgSO_4$, filtered and evaporated to dryness. The crude was used in the next step without further purification. Crude ^{1}H NMR (500 MHz, Chloroform-d) δ 8.11 (s, 1H), 7.79 (d, *J* = 8.6 Hz, 1H), 6.87 (d, *J* = 8.6 Hz, 1H), 3.95 (s, 3H), 3.71 – 3.64 (m, 4H), 2.54 – 2.36 (m, 4H), 2.27 – 2.22 (m, 6H). LC-MS (ESI) *m*/*z* found: 370 $[M+H]^+$; retention time: 3.40 minutes.

methyl 4-(3-bromo-4-methoxybenzoyl)tetrahydro-2H-pyran-4-carboxylate (**80**)

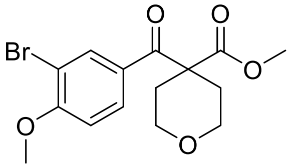

Benzoic acid **75** (1.0 g, 4.3 mmol) was suspended in DCM (10 mL) while cooling to 0°C. Subsequently oxalyl dichloride (0.48 mL, 5.6 mmol) and a few drops of DMF were added and the mixture was allowed to warm up to room temperature. The mixture was stirred for 2 h after which volatiles were evaporated. The remaining solids were re-

dissolved in 10 mL of THF. In a separate flask methyl tetrahydro-2H-pyran-4-carboxylate (0.81 g, 5.6 mmol) was stirred in THF (10 mL) at -78°C and a 2M LDA (2.6 mL, 5.2 mmol) was added dropwise while maintaining -78°C. Upon full addition, the mixture was stirred for 45 m after which the acid chloride in THF was added dropwise, maintaining the temperate at -78°C. The reaction was allowed to warm up to room temperature after which it was quenched with water and extracted with diethyl ether. The organic phase was washed twice with sat. aq. Na_2CO_3 and once with brine. The organic layer was then dried with $MgSO_4$, filtered and evaporated to dryness. The crude was used in the next step without further purification. Crude ^1H NMR (500 MHz, Chloroform-d) δ 8.14 (d, *J* = 2.3 Hz, 1H), 7.81 (dd, *J* = 8.7, 2.2 Hz, 1H), 6.92 – 6.86 (m, 1H), 3.97 (s, 3H), 3.85 – 3.77 (m, 2H), 3.76 – 3.66 (m, 2H), 2.29 – 2.18 (m, 4H), 1.44 (s, 3H).

4-(3-bromo-4-methoxyphenyl)-2,3-diazaspiro[4.4]non-3-en-1-one (**81**)

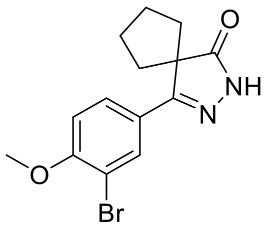

Keto-ester **77** (500 mg, 1.41 mmol) was dissolved in ethanol (8 mL) and hydrazine (0.69 mL, 14.1 mmol) was added. The mixture was heated in the microwave at 120°C for 2 hours. The resulting mixture was evaporated to dryness, coated on SiO_2 and purifiied over SiO_2 using a gradient of 20% EtOAc in heptane towards 90% EtOAc in heptane to yield 180 mg (0.56 mmol, 40%) of the title compound as a white solid. ^1H NMR (500 MHz, Chloroform-d) δ 9.08 (s, 1H), 7.93 (d, *J* = 2.2 Hz, 1H), 7.59 (dd, *J* = 8.6, 2.2 Hz, 1H), 6.89 (d, *J* = 8.6 Hz, 1H), 3.90 (s, 3H), 2.23 – 2.04 (m, 6H), 2.04 – 1.90 (m, 2H). ^{13}C NMR (126 MHz, Chloroform-d) δ 183.3, 161.2, 157.0, 131.3, 126.5, 124.5, 112.2, 111.7, 56.4, 55.5, 35.7, 27.5. LC-MS (ESI) *m/z* found: 323 $[M+H]^+$; retention time: 5.17 minutes. HRMS-ESI $[M+H]^+$ calculated for $C_{14}H_{16}N_2O_2$: 323.0390, found: 323.0372.

4-(3-bromo-4-methoxyphenyl)-2,3-diazaspiro[4.4]nona-3,7-dien-1-one (**82**)

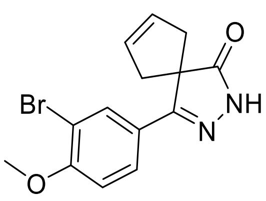

Keto-ester **78** (900 mg, 2.52 mmol) was dissolved in ethanol (3 mL) and hydrazine (0.63 mL, 20.2 mmol) was added. The mixture was stirred overnight. The resulting mixture was quenched with water and precipitated solids were collected and washed with water (20 mL) to yield 900 mg (2.8 mmol, 65% over two steps) of the title compound as a yellow-white solid. ^{1}H NMR (500 MHz, Chloroform-d) δ 11.46 (s, 1H), 7.82 (d, *J* = 2.2 Hz, 1H), 7.53 (dd, *J* = 8.7, 2.2 Hz, 1H), 7.20 (d, *J* = 8.7 Hz, 1H), 5.92 (s, 2H), 3.88 (s, 3H), 2.80 – 2.70 (m, 4H). LC-MS (ESI) *m*/*z* found: 321 $[M+H]^+$; retention time: 4.17 minutes. HRMS-ESI $[M+H]^+$ calculated for $C_{14}H_{14}BrN_2O_2$: 321.0233, found: 321.0247.

4-(3-bromo-4-methoxyphenyl)-8-methyl-2,3,8-triazaspiro[4.5]dec-3-en-1-one (**83**)

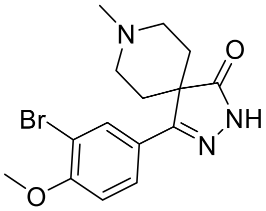

Keto-ester **79** (900 mg, 2.43 mmol) was dissolved in ethanol (3 mL) and hydrazine (0.61 mL, 19.5 mmol) was added. The mixture was stirred overnight. The resulting mixture was evaporated to dryness, coated on SiO_2 and purifiied over SiO_2 using a gradient of 60% EtOAc in heptane towards 10% MeOH in EtOAc to yield 470 mg (1.33 mmol, 31% over two steps) of the title compound as a yellow-white solid. ^{1}H NMR (500 MHz, DMSO-d$_6$) δ 11.47 (s, 1H), 7.94 (d, *J* = 2.2 Hz, 1H), 7.76 (dd, *J* = 8.7, 2.2 Hz, 1H), 7.21 (d, *J* = 8.8 Hz, 1H), 3.90 (s, 3H), 2.72 (td, *J* = 11.9, 2.6 Hz, 2H), 2.60 – 2.54 (m, 2H), 2.25 (s, 3H), 2.21 (td, *J* = 13.4, 4.6 Hz, 2H), 1.64 (d, *J* = 14.1 Hz, 2H). ^{13}C NMR (126 MHz, DMSO-d$_6$) δ 180.3, 158.6, 156.6, 130.9, 127.6, 125.1, 113.1, 111.5, 56.9, 48.8, 46.6, 40.5, 29.3. LC-MS (ESI) *m*/*z* found:

352 [M+H]$^+$; retention time: 2.90 minutes. HRMS-ESI [M+H]$^+$ calculated for $C_{15}H_{19}BrN_3O_2$: 352.0655, found: 352.0667.

4-(3-bromo-4-methoxyphenyl)-8-oxa-2,3-diazaspiro[4.5]dec-3-en-1-one (Tcr385) (**84**)

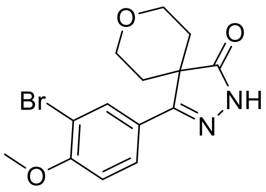

Keto-ester **80** (900 mg, 2.52 mmol) was dissolved in ethanol (3 mL) and hydrazine (0.63 mL, 20.2 mmol) was added. The mixture was stirred overnight. The resulting mixture was evaporated to dryness, coated on SiO_2 and purifiied over SiO_2 using a gradient of 30% EtOAc in heptane towards 90% EtOAc in heptane to yield 350 mg (0.87 mmol, 20% over two steps) of the title compound as a yellow-white solid.^{1}H NMR (500 MHz, DMSO-*d6*) δ 11.58 (s, 1H), 7.94 (d, 1H), 7.76 (dd, 1H), 7.20 (d, *J* = 8.7 Hz, 1H), 4.14 (t, *J* = 11.2 Hz, 2H), 3.89 (s, 3H), 3.73 (dd, *J* = 11.4, 4.9 Hz, 2H), 2.22 (td, *J* = 13.4, 5.0 Hz, 2H), 1.59 (d, *J* = 13.8 Hz, 2H). ^{13}C NMR (126 MHz, DMSO) δ 180.4, 157.9, 156.7, 131.0, 127.7, 124.9, 113.1, 111.6, 61.0, 56.9, 46.7, 29.0. LC-MS (ESI) *m*/*z* found: 314 [M+H]$^+$; retention time: 3.85 minutes.

4-(3-bromo-4-methoxyphenyl)-2-isopropyl-2,3-diazaspiro[4.4]non-3-en-1-one (**85**)

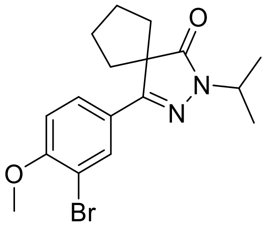

Dihydropyrazolone **81** (125 mg, 0.39 mmol) was stirred in DMF (1.5 mL) and sodium hydride (60% in mineral oil) (23 mg, 0.58 mmol) was added. After stirring for 30 m, 2-bromopropane (0.058 mL, 0.62 mmol) was added and the mixture was heated to 50°C for 2 hours. The reaction was quenched with water (20 mL) and the precipitate was collected and washed with water (5 mL). The resulting solids were dried *in vacuo* yielding 135 mg (0.37 mmol, 96%) of the title compound as a white solid. ^{1}H NMR (500 MHz, Chloroform-d) δ 8.02

(d, J = 2.2 Hz, 1H), 7.60 (dd, J = 8.7, 2.2 Hz, 1H), 6.91 (d, J = 8.7 Hz, 1H), 4.47 (h, J = 6.7 Hz, 1H), 3.93 (s, 3H), 2.25 – 1.88 (m, 8H), 1.35 (d, J = 6.8 Hz, 6H). ^{13}C NMR (126 MHz, Chloroform-d) δ 179.6, 159.2, 156.7, 131.2, 126.3, 124.8, 112.2, 111.5, 57.1, 56.4, 45.1, 35.7, 27.5, 20.85. LC-MS (ESI) *m*/*z* found: 365 $[M+H]^+$; retention time: 5.85 minutes. HRMS-ESI $[M+H]^+$ calculated for $C_{17}H_{22}BrN_2O_2$: 365.0859, found: 365.0842.

4-(3-bromo-4-methoxyphenyl)-2-isopropyl-2,3-diazaspiro[4.4]nona-3,7-dien-1-one (Tcr403) (**86**)

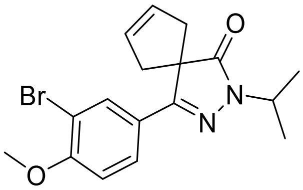

Dihydropyrazolone **82** (720 mg, 2.24 mmol) was stirred in DMF (5 mL) and sodium hydride (60% in mineral oil) (99 mg, 2.5 mmol) was added. After stirring for 30 m, 2-bromopropane (0.25 mL, 2.7 mmol) was added and the mixture was stirred overnight. The reaction was quenched with water (10 mL) and extracted with EtOAc, (15 mL) the organic layer was washed with sat. aq. Na_2CO_3 (2x 10 mL), brine (10 mL) and dried over $MgSO_4$. Evaporation of volatiles yielded 710 mg (2.0 mmol, 87%) of the title compound as an off-white solid. ^{1}H NMR (500 MHz, Chloroform-d) δ 8.04 (d, J = 2.2 Hz, 1H), 7.54 (dd, J = 8.6, 2.2 Hz, 1H), 6.87 (d, J = 8.7 Hz, 1H), 5.89 (s, 2H), 4.48 (hept, J = 6.7 Hz, 1H), 3.92 (s, 3H), 2.96 – 2.87 (m, 2H), 2.87 – 2.79 (m, 2H), 1.36 (d, J = 6.7 Hz, 6H). ^{13}C NMR (126 MHz, Chloroform-d) δ 178.8, 160.0, 157.0, 130.7, 129.3, 125.8, 124.3, 112.4, 111.6, 56.3, 54.1, 45.3, 43.1, 20.9. LC-MS (ESI) *m*/*z* found: 363 $[M+H]^+$; retention time: 5.28 minutes. HRMS-ESI $[M+H]^+$ calculated for $C_{17}H_{20}BrN_2O_2$: 363.0703, found: 363.0715.

4-(3-bromo-4-methoxyphenyl)-2-isopropyl-8-methyl-2,3,8-triazaspiro[4.5]dec-3-en-1-one (Tcr402) (**87**)

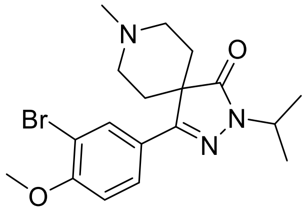

Dihydropyrazolone **83** (400 mg, 1.14 mmol) was stirred in DMF (2.5 mL) and sodium hydride (60% in mineral oil) (50 mg, 1.25 mmol) was added. After stirring for 30 m, 2-bromopropane (0.13 mL, 1.36 mmol) was added and the mixture was stirred overnight. The reaction was quenched with water (10 mL) and extracted with EtOAc, (15 mL) the organic layer was washed with sat. aq. Na_2CO_3 (2x 10 mL), brine (10 mL) and dried over $MgSO_4$. Evaporation of volatiles yielded 330 mg (0.84 mmol, 74%) of the title compound as an off-white solid. ^{1}H NMR (500 MHz, Chloroform-d) δ 8.18 (s, 1H), 7.79 (d, *J* = 8.4 Hz, 1H), 6.87 (d, *J* = 8.6 Hz, 1H), 4.48 (hept, *J* = 6.7 Hz, 1H), 3.94 (s, 3H), 3.05 – 2.91 (m, 2H), 2.74 (d, *J* = 9.9 Hz, 2H), 2.52 – 2.38 (m, 5H), 1.71 (d, *J* = 13.7 Hz, 2H), 1.36 (d, *J* = 6.7 Hz, 6H). ^{13}C NMR (126 MHz, Chloroform-d) δ 176.6, 157.6, 156.7, 131.0, 127.7, 124.7, 113.1, 111.6, 56.9, 48.9, 48.5, 46.6, 44.6, 29.4, 21.1. LC-MS (ESI) *m/z* found: 394 $[M+H]^+$; retention time: 3.86 minutes.

4-(3-bromo-4-methoxyphenyl)-2-isopropyl-8-oxa-2,3-diazaspiro[4.5]dec-3-en-1-one (**88**)

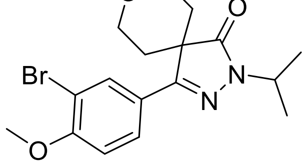

Dihydropyrazolone **84** (300 mg, 0.88 mmol) was stirred in DMF (2 mL) and sodium hydride (60% in mineral oil) (39 mg, 0.97 mmol) was added. After stirring for 30 m, 2-bromopropane (0.10 mL, 1.06 mmol) was added and the mixture was stirred overnight. The reaction was quenched with water (10 mL) and extracted with EtOAc, (15 mL) the organic layer was washed with sat. aq. Na_2CO_3 (2x 10 mL), brine (10 mL) and dried over $MgSO_4$. Evaporation of volatiles yielded 310 mg (0.88 mmol, 92%) of the title compound as

an off-white solid. ^{1}H NMR (500 MHz, Chloroform-d) δ 7.89 – 7.81 (m, 2H), 7.01 (d, *J* = 8.6 Hz, 1H), 4.48 (p, *J* = 6.5 Hz, 1H), 4.39 (t, *J* = 12.0 Hz, 2H), 3.87 (m, 3.91 – 3.81, 5H), 2.46 (td, *J* = 13.1, 4.8 Hz, 2H), 1.61 (d, *J* = 13.8 Hz, 2H), 1.34 (d, *J* = 6.6 Hz, 6H). ^{13}C NMR (126 MHz, Chloroform-d) δ 177.0, 157.9, 157.7, 136.8, 129.1, 127.9, 123.6, 110.8, 61.6, 55.8, 48.6, 44.8, 29.4, 20.8. LC-MS (ESI) *m/z* found: 381 $[M+H]^+$; retention time: 5.02 minutes. HRMS-ESI $[M+H]^+$ calculated for $C_{17}H_{22}BrN_2O_3$: 381.0808, found: 381.0818.

2-isopropyl-4-(4-methoxy-3-(pyridin-3-yl)phenyl)-2,3-diazaspiro[4.4]non-3-en-1-one (**89**)

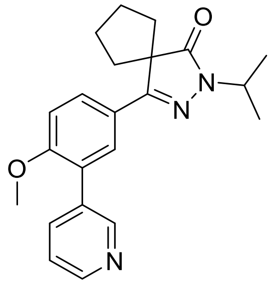

Dihydropyrazolone **85** (50 mg, 0.14 mmol) and pyridin-3-ylboronic acid (18 mg, 0.15 mmol) were charged to a microwave tube after which DME (2 mL) and 1M Na_2CO_3 (0.4 mL, 0.4 mmol) were added. The mixture was degassed with N_2 for 5 m after which $Pd(dppf)Cl_2$ (8 mg, 10 µmol) was added. The reaction was heated at 120°C for 1 hour in the microwave. The reaction mixture was diluted with EtOAc (25 mL) and filtered over Celite. The residue was washed with saturated $NaHCO_3$ (2x 20 mL) and brine (1x 20 mL). The organic phase was dried over Na_2SO_4, filtered and concentrated *in vacuo.* The remaining crude was purified over SiO_2 using a gradient of 30% EtOAc in heptane towards 100% EtOAc to yield 25 mg (0.069 mmol, 50%) of the title compound as a white solid. ^{1}H NMR (500 MHz, Chloroform-d) δ 8.76 (d, *J* = 2.2 Hz, 1H), 8.57 (dd, *J* = 4.8, 1.7 Hz, 1H), 7.86 (dt, *J* = 7.8, 1.9 Hz, 1H), 7.78 (d, *J* = 2.3 Hz, 1H), 7.69 (dd, *J* = 8.6, 2.3 Hz, 1H), 7.35 (dd, *J* = 7.9, 4.7 Hz, 1H), 7.00 (d, *J* = 8.7 Hz, 1H), 4.45 (p, *J* = 6.7 Hz, 1H), 3.85 (s, 3H), 2.24 – 1.87 (m, 8H), 1.33 (d, *J* = 6.7 Hz, 6H). ^{13}C NMR (126 MHz, Chloroform-d) δ 179.7, 160.2, 157.6, 150.1, 148.3, 137.0, 133.8, 128.7, 127.5, 127.5, 123.9, 123.1, 111.2, 57.3, 55.8, 45.2, 35.9, 27.6, 20.9. LC-MS (ESI)

m/*z* found: 364 [M+H]$^+$; retention time: 4.47 minutes. HRMS-ESI [M+H]$^+$ calculated for $C_{22}H_{36}N_3O_2$: 364.2020, found: 364.2012.

2-isopropyl-4-(4-methoxy-3-(pyridin-3-yl)phenyl)-2,3-diazaspiro[4.4]nona-3,7-dien-1-one (**90**)

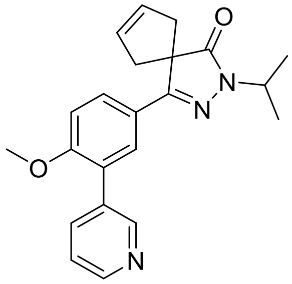

Dihydropyrazolone **86** (150 mg, 0.41 mmol) and pyridin-3-ylboronic acid (76 mg, 0.62 mmol) were charged to a microwave tube after which DME (4 mL) and 1M Na_2CO_3 (1.3 mL, 1.3 mmol) were added. The mixture was degassed with N_2 for 5 m after which Pd(dppf)Cl_2 (34 mg, 41 µmol) was added. The reaction was heated at 120°C for 1 hour in the microwave. The reaction mixture was diluted with EtOAc (25 mL) and filtered over Celite. The residue was washed with saturated $NaHCO_3$ (2x 20 mL) and brine (1x 20 mL). The organic phase was dried over Na_2SO_4, filtered and concentrated *in vacuo.* The remaining crude was purified over SiO_2 using a gradient of 30% EtOAc in heptane towards 100% EtOAc to yield 89 mg (0.25 mmol, 60%) of the title compound as a transparent oil which solidified over time. ^{1}H NMR (500 MHz, Chloroform-d) δ 8.75 (s, 1H), 8.57 (d, J = 4.2 Hz, 1H), 7.85 (dt, J = 8.0, 1.9 Hz, 1H), 7.80 (d, J = 2.2 Hz, 1H), 7.65 (dd, J = 8.6, 2.0 Hz, 1H), 7.35 (dd, J = 7.8, 4.9 Hz, 1H), 6.98 (d, J = 8.7 Hz, 1H), 5.88 (s, 2H), 4.49 (hept, J = 6.7 Hz, 1H), 3.85 (s, 3H), 3.00 – 2.81 (m, 4H), 1.35 (d, J = 6.7 Hz, 6H). ^{13}C NMR (126 MHz, Chloroform-d) δ 178.9, 161.0, 157.8, 150.1, 148.2, 136.9, 133.7, 129.4, 128.1, 127.6, 126.9, 123.4, 123.0, 111.2, 55.7, 54.2, 45.3, 43.2, 20.9. LC-MS (ESI) *m*/*z* found: 362 [M+H]$^+$; retention time: 4.00 minutes. HRMS-ESI [M+H]$^+$ calculated for $C_{22}H_{24}N_3O_2$: 362.1863, found: 362.1874.

2-isopropyl-4-(4-methoxy-3-(pyridin-3-yl)phenyl)-8-methyl-2,3,8-triazaspiro[4.5]dec-3-en-1-one (**91**)

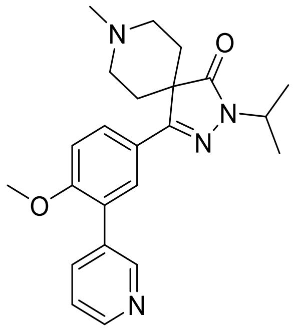

Dihydropyrazolone **87** (150 mg, 0.38 mmol) and pyridin-3-ylboronic acid (70 mg, 0.57 mmol) were charged to a microwave tube after which DME (4 mL) and 1M Na_2CO_3 (1.2 mL, 1.2 mmol) were added. The mixture was degassed with N_2 for 5 m after which Pd(dppf)Cl_2 (31 mg, 38 µmol) was added. The reaction was heated at 120°C for 1 hour in the microwave. The reaction mixture was diluted with EtOAc (25 mL) and filtered over Celite. The residue was washed with saturated $NaHCO_3$ (2x 20 mL) and brine (1x 20 mL). The organic phase was dried over Na_2SO_4, filtered and concentrated *in vacuo.* The remaining crude was purified over SiO_2 using a gradient of 60% EtOAc in heptane towards 10% MeOH in EtOAc to yield 38 mg (0.097 mmol, 26%) of the title compound as a transparent oil which solidified over time. ^{1}H NMR (500 MHz, Chloroform-d) δ 8.75 (d, J = 1.7 Hz, 1H), 8.57 (dd, J = 4.8, 1.5 Hz, 1H), 7.90 (d, J = 2.2 Hz, 1H), 7.88 – 7.80 (m, 2H), 7.35 (dd, J = 7.8, 4.9 Hz, 1H), 6.97 (d, J = 8.7 Hz, 1H), 4.46 (hept, J = 6.7 Hz, 1H), 3.84 (s, 3H), 3.00 (t, J = 11.4 Hz, 2H), 2.73 (d, J = 9.3 Hz, 2H), 2.49 (td, J = 13.3, 4.1 Hz, 2H), 2.41 (s, 3H), 1.72 (d, J = 14.1 Hz, 2H), 1.33 (d, J = 6.7 Hz, 6H). ^{13}C NMR (126 MHz, Chloroform-d) δ 176.79, 158.73, 157.60, 150.23, 148.31, 136.91, 133.58, 129.18, 128.14, 127.76, 123.80, 122.96, 110.71, 55.74, 48.87, 48.77, 46.35, 44.77, 29.72, 20.75. LC-MS (ESI) *m*/*z* found: 393 $[M+H]^+$; retention time: 3.10 minutes. HRMS-ESI $[M+H]^+$ calculated for $C_{23}H_{29}N_4O_2$: 393.2285, found: 393.2302.

2-isopropyl-4-(4-methoxy-3-(pyridin-3-yl)phenyl)-8-oxa-2,3-diazaspiro[4.5]dec-3-en-1-one (**92**)

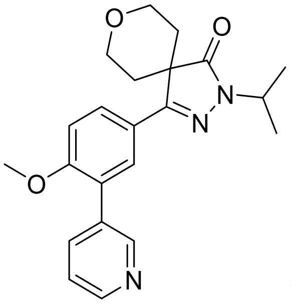

Dihydropyrazolone **88** (200 mg, 0.53 mmol) and pyridin-3-ylboronic acid (97 mg, 0.79 mmol) were charged to a microwave tube after which DME (4 mL) and 1M Na_2CO_3 (1.6 mL, 1.6 mmol) were added. The mixture was degassed with N_2 for 5 m after which Pd(dppf)Cl_2 (43 mg, 52 µmol) was added. The reaction was heated at 120°C for 1 hour in the microwave. The reaction mixture was diluted with EtOAc (25 mL) and filtered over Celite. The residue was washed with saturated $NaHCO_3$ (2x 20 mL) and brine (1x 20 mL). The organic phase was dried over Na_2SO_4, filtered and concentrated *in vacuo.* The remaining crude was purified over SiO_2 using a gradient of 30% EtOAc in heptane towards 100% EtOAc to yield 43 mg (0.11 mmol, 22%) of the title compound as a transparent oil which solidified over time. ^{1}H NMR (500 MHz, Chloroform-d) δ 9.04 (s, 1H), 8.86 (s, 1H), 7.93 – 7.82 (m, 3H), 7.47 (d, *J* = 7.4 Hz, 1H), 7.03 (d, *J* = 8.6 Hz, 1H), 4.50 (hept, *J* = 6.7 Hz, 1H), 4.41 (td, *J* = 12.1, 2.2 Hz, 2H), 3.94 – 3.79 (m, 5H), 2.47 (td, *J* = 13.3, 5.2 Hz, 2H), 1.62 (d, *J* = 13.9 Hz, 2H), 1.36 (d, *J* = 6.7 Hz, 6H). ^{13}C N MR (126 MHz, Chloroform-d) δ 177.0, 157.9, 157.7, 150.1, 148.2, 136.7, 133.3, 131.4, 129.1, 128.2, 127.9, 123.6, 110.9, 61.6, 55.8, 48.6, 44.8, 29.4, 20.8. LC-MS (ESI) *m*/*z* found: 380 $[M+H]^+$; retention time: 3.46 minutes. HRMS-ESI $[M+H]^+$ calculated for $C_{22}H_{26}N_3O_3$: 380.1969, found: 380.1983.

5-(3-bromo-4-methoxyphenyl)-1H-pyrazol-3-ol (**93**)

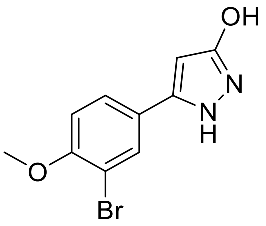

Keto-ester **76** (2.5 g, 8.3 mmol) was dissolved in ethanol (10 mL) and hydrazine (1.3 ml, 41.5 mmol) was added. The reaction mixture was stirred for 2 hours after which 150 mL of water was added followed by MTBE (200 mL). This resulted in a two-layer system with a substantial amount of solids in between. Extraction of the aqueous layer with MTBE (3x 150 mL) and subsequent drying over $MgSO_4$ and evaporation of volatiles yielded 800 mg (3.0 mmol, 36%) of the title compound. Collection of the solids between both layers yielded another 1.3 g (4.8 mmol, 58%) of the title compound. Combination of both batches yielded 2.1 g (7.8 mmol, 94%) of the title compound as an off-white solid. ^{1}H NMR (500 MHz, Chloroform-d) δ 7.91 (d, J = 2.2 Hz, 1H), 7.66 (dd, J = 8.6, 2.2 Hz, 1H), 7.15 (d, J = 8.6 Hz, 1H), 5.88 (s, 1H), 3.88 (s, 3H). LC-MS (ESI) *m*/*z* found: 269 $[M+H]^{+}$; retention time: 4.43 minutes.

6-(3-bromo-4-methoxyphenyl)-2,3-dihydropyrazolo[5,1-b]oxazole (**94**)

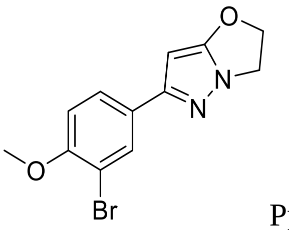

Pyrazole **93** (100 mg, 0.37 mmol) was dissolved in DMF (2 ml), potassium carbonate (128 mg, 0.93 mmol) and 1,2-dibromoethane (0.034 ml, 0.39 mmol) were added. Reaction was refluxed for 8 hours after which a second equivalent of 1,2-dibromoethane (0.034 ml, 0.39 mmol) was added. Reaction was refluxed for another 4 hours. The crude reaction mixture was diluted with MTBE (20 mL) and extracted with NaHCO3 (3x 30 ml). Organic layer was washed with brine and dried over $MgSO_4$. The remaining crude was purified

over SiO_2 using a gradient of 30% EtOAc in heptane towards 80% EtOAc in heptane, yielding 40 mg (0.14 mmol, 37%) of the title compound as a white solid. ^{1}H NMR (500 MHz, Chloroform-d) δ 7.93 (d, *J* = 2.1 Hz, 1H), 7.63 (dd, *J* = 8.5, 2.1 Hz, 1H), 6.90 (d, *J* = 8.5 Hz, 1H), 5.62 (s, 1H), 5.09 – 4.99 (m, 2H), 4.37 – 4.27 (m, 2H), 3.91 (s, 3H). LC-MS (ESI) *m/z* found: 295 $[M+H]^+$; retention time: 4.81 minutes. HRMS-ESI $[M+H]^+$ calculated for $C_{12}H_{12}BrN_2O_2$: 295.0077, found: 295.0059.

6-(4-methoxy-3-(pyridin-3-yl)phenyl)-2,3-dihydropyrazolo[5,1-b]oxazole (**95**)

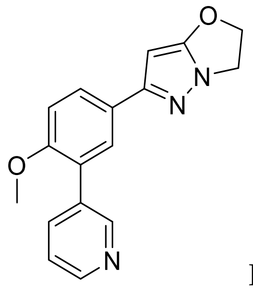

Dihydropyrazolooxazole **94** (140 mg, 0.48 mmol) and pyridin-3-ylboronic acid (70 mg, 0.57 mmol) were charged to a microwave vial after which DME (3 mL) and 1M Na_2CO_3 (1.4 mL, 1.4 mmol) were added. The mixture was degassed with N_2 for 5 m after which $Pd(dppf)Cl_2$ (39 mg, 0.05 mmol) was added. The reaction was heated in the microwave for 1 h at 120°C. The reaction mixture was diluted with MTBE (25 mL) and filtered over Celite. The residue was washed with saturated $NaHCO_3$ (2x) and brine (1x). The organic phase was dried over Na_2SO_4, filtered and concentrated *in vacuo* to be further purified over SiO_2 using a gradient of 15% EtOAc in heptane towards 100% EtOAc to yield 15 mg (0.05 mmol, 11%) of the title compound. ^{1}H NMR (500 MHz, Chloroform-d) δ 8.92 (s, 1H), 8.66 (s, 1H), 7.90 (d, *J* = 7.8 Hz, 1H), 7.72 (d, *J* = 7.5 Hz, 2H), 7.45 – 7.32 (m, 1H), 7.02 (d, *J* = 8.5 Hz, 1H), 5.67 (s, 1H), 5.04 (t, *J* = 7.9 Hz, 2H), 4.32 (t, *J* = 7.9 Hz, 2H), 3.84 (s, 3H). ^{13}C NMR (126 MHz, Chloroform-d) δ 159.5, 156.3, 154.7, 150.1, 148.3, 137.1, 130.6, 127.8, 127.3, 126.9, 126.4, 116.9, 112.4, 77.6, 76.1, 56.2, 45.2. LC-MS (ESI) *m/z* found: 294 $[M+H]^+$; retention time: 3.71 minutes. HRMS-ESI $[M+H]^+$ calculated for $C_{17}H_{16}N_3O_2$: 294.1237, found: 294.1233.

2-(4-methoxy-3-(pyridin-3-yl)phenyl)acetonitrile (**97**)

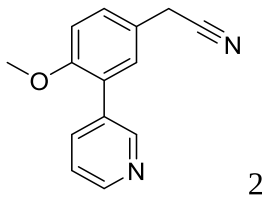

2-(3-bromo-4-methoxyphenyl)acetonitrile (**96**) (500 mg, 2.2 mmol) and pyridin-3-ylboronic acid (353 mg, 2.9 mmol) were charged to a microwave vial after which DME (10 mL) and 1M Na_2CO_3 (6.6 mL, 6.6 mmol) were added. The mixture was degassed with N_2 for 5 m after which Pd(dppf)Cl_2 (90 mg, 0.11 mmol) was added. The reaction was heated in the microwave for 1 h at 120°C. The reaction mixture was diluted with EtOAc (50 mL) and filtered over Celite. The residue was washed with saturated $NaHCO_3$ (2x 40 mL) and brine (40 mL). The organic phase was dried over Na_2SO_4, filtered and concentrated *in vacuo* to be further purified over SiO_2 using a gradient of 35% EtOAc in heptane towards 100% EtOAc to yield 450 mg (2.01 mmol, 91%) of the title compound. ^{1}H NMR (500 MHz, Chloroform-d) δ 8.80 – 8.70 (m, 1H), 8.57 (d, *J* = 4.7 Hz, 1H), 7.89 – 7.79 (m, 1H), 7.39 – 7.30 (m, 2H), 7.26 – 7.22 (m, 1H), 7.00 (dd, *J* = 8.5, 0.9 Hz, 1H), 3.82 (d, *J* = 1.3 Hz, 3H), 3.75 (d, *J* = 0.8 Hz, 2H). ^{13}C NMR (126 MHz, Chloroform-d) δ 156.3, 149.9, 148.1, 136.9, 133.5, 130.2, 129.0, 127.7, 123.1, 122.3, 118.0, 111.8, 55.8, 22.9. LC-MS (ESI) *m/z* found: 225 $[M+H]^+$; retention time: 2.51 minutes. HRMS-ESI $[M+H]^+$ calculated for $C_{14}H_{13}N_2O$: 225.1022, found: 225.1033.

5-(4-methoxy-3-(pyridin-3-yl)benzyl)-1,3,4-thiadiazol-2-amine (**98**)

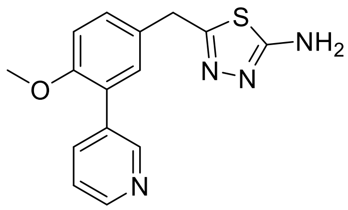

Acetonitrile **97** (250 mg, 1.12 mmol) was dissolved in TFA (2 ml) and hydrazinecarbothioamide (122 mg, 1.34 mmol) was added. The mixture was stirred at 60°C for 6 h after which the reaction was cooled down to rt and quenched with sat. aq. Na_2CO_3. The

aqueous layer was extracted with DCM (40 mL) after which the organic layer was washed with brine (40 mL) and dried over Na_2SO4. After evaporation of volatiles the remaining crude was purified over SiO_2 using a gradient of EtOAc towards 15% MeOH in EtOAc. ^{1}H NMR (500 MHz, Chloroform-d) δ 8.79 (d, *J* = 2.2 Hz, 1H), 8.64 (dd, *J* = 5.1, 1.6 Hz, 1H), 8.13 (dt, *J* = 8.0, 1.9 Hz, 1H), 7.64 (dd, *J* = 8.0, 5.0 Hz, 1H), 7.34 (dd, *J* = 6.0, 2.4 Hz, 2H), 7.14 (d, *J* = 9.1 Hz, 1H), 4.17 (s, 2H), 3.78 (s, 3H). ^{13}C NMR (126 MHz, Chloroform-d) δ 169.3, 158.4, 155.6, 148.3, 146.7, 139.1, 134.6, 131.2, 130.9, 130.7, 126.0, 124.4, 112.6, 56.2, 35.0. LC-MS (ESI) *m/z* found: 299 $[M+H]^+$; retention time: 2.31 minutes. HRMS-ESI $[M+H]^+$ calculated for $C_{15}H_{15}N_4OS$: 299.0946, found: 299.0961.

2-isopropyl-5-(4-methoxy-3-(pyridin-3-yl)phenyl)-4,4-dimethyl-2,4-dihydro-3H-pyrazole-3-thione (**99**)

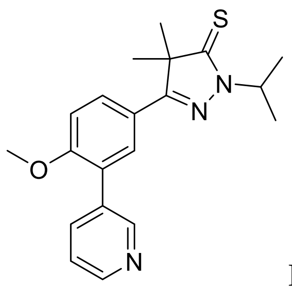

NPD-0227 (**3**) (50 mg, 0.15 mmol) was dissolved in THF (2 ml) and Lawessons Reagent (120 mg, 0.30 mmol) was added. Reaction mixture was refluxed for 32 hour after which it was diluted with MTBE (40 mL) and extracted with sat. aq. $NaHCO_3$ (3x 25 mL). The organic layer was dried over $MgSO_4$ and volatiles were evaporated. The resulting crude was purified over SiO_2 using a gradient of 30% EtOAc in n-heptane towards 90% EtOAc in n-heptane, yielding 40 mg (0.11 mmol, 76%) of the title compound as an off-white solid. ^{1}H NMR (500 MHz, Chloroform-d) δ 8.78 (d, *J* = 2.3 Hz, 1H), 8.60 (dd, *J* = 4.8, 1.7 Hz, 1H), 7.96 – 7.86 (m, 3H), 7.43 – 7.35 (m, 1H), 7.05 (d, *J* = 8.5 Hz, 1H), 5.23 (hept, *J* = 6.6 Hz, 1H), 3.88 (s, 3H), 1.57 (s, 6H), 1.42 (d, *J* = 6.6 Hz, 6H). ^{13}C NMR (126 MHz, Chloroform-d) δ 201.9, 169.3, 158.5, 150.1, 148.4, 137.1, 133.6, 129.6, 128.6, 127.8, 123.5, 123.2, 111.3, 62.0, 55.9,

49.7, 26.9, 20.1. LC-MS (ESI) *m/z* found: 354 [M+H]$^+$; retention time: 4.97 minutes. HRMS-ESI [M+H]$^+$ calculated for $C_{20}H_{24}N_3OS$: 354.1635, found: 354.1628.

4-(3-bromo-4-methoxyphenyl)-4-oxobutanoic acid (**101**)

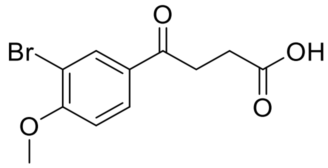

1-bromo-2-methoxybenzene (3.3 ml, 26.7 mmol) was dissolved in nitrobenzene (25 mL) and $AlCl_3$ (8.9 g, 66.8 mmol) was added and the mixture was cooled to 0°C. Succinic anhydride (3.2 g, 32.1 mmol) was dissolved in nitrobenzene (30 mL) and was added dropwise to the cooled reaction mixture. Upon completion of addition the mixture was heated to 60oCfor 3 h, after which it was allowed to cool down and quenched in ice-cold 1M HCl. The resulting solids were filtered off and washed with n-heptane (100 mL). The solids were dried in vacuo to yield 3.2 g (11.2 mmol, 42%) as a white solid.^{1}H NMR (500 MHz, Chloroform-d) δ 12.16 (s, 1H), 8.14 (d, *J* = 2.1 Hz, 1H), 8.03 (dd, *J* = 8.7, 2.1 Hz, 1H), 7.25 (d, *J* = 8.7 Hz, 1H), 3.95 (s, 3H), 3.22 (t, *J* = 6.7 Hz, 2H), 2.56 (t, *J* = 5.6 Hz, 2H). ^{13}C NMR (126 MHz, Chloroform-d) δ 196.6, 174.3, 159.5, 133.0, 130.9, 130.1, 112.8, 111.3, 57.2, 33.2, 28.3. LC-MS (ESI) *m/z* found: 287 [M+H]$^+$; retention time: 4.56 minutes. HRMS-ESI [M+H]$^+$ calculated for $C_{11}H_{12}BrO_4$: 286.9913, found: 286.9914

6-(3-bromo-4-methoxyphenyl)-4,5-dihydropyridazin-3(2H)-one (**102**)

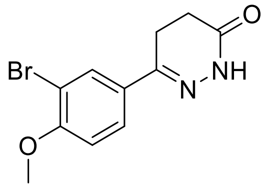

Keto-acid **101** (2.0 g, 7.0 mmol) was dissolved in EtOH (15 ml) and hydrazine monohydrate (1.0 ml, 20.9 mmol) was added. The mixture was heated at 85°C for 1 h after which the reaction mixture was cooled to rt upon which a white precipitate appeared. Solids were collected, washed with water (25 mL) and dried *in vacuo* to yield 1.8 g (6.2 mmol,

89%) of the title compound as a white solid. ^{1}H NMR (500 MHz, Chloroform-d) δ 10.91 (s, 1H), 7.94 (d, *J* = 2.2 Hz, 1H), 7.73 (dd, *J* = 8.7, 2.2 Hz, 1H), 7.16 (d, *J* = 8.7 Hz, 1H), 3.89 (s, 3H), 2.92 (t, *J* = 8.2 Hz, 2H), 2.43 (t, *J* = 8.2 Hz, 2H). ^{13}C NMR (126 MHz, Chloroform-d) δ 167.0, 156.1, 148.0, 130.0, 129.9, 126.6, 112.4, 110.8, 56.4, 26.0, 21.7. LC-MS (ESI) *m*/*z* found: 283 $[M+H]^+$; retention time: 4.53 minutes. HRMS-ESI $[M+H]^+$ calculated for $C_{11}H_{12}BrN_2O_2$: 283.0077, found: 283.0059.

6-(3-bromo-4-methoxyphenyl)-2-isopropyl-4,5-dihydropyridazin-3(2H)-one (**103**)

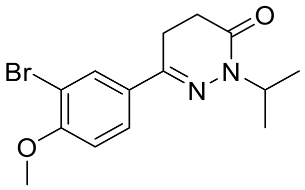

Dihydropyridazinone **102** (250 mg, 0.88 mmol) was stirred in DMF (2 mL) and sodium hydride (60% in mineral oil) (49 mg, 1.2 mmol) was added. Stirred for 10 minutes, after which 2-bromopropane (0.12 mL, 1.2 mmol) was added and the reaction was heated at 50°C for 3 hours. The reaction was quenched with water, extracted with EtOAc (30 mL), which was washed with water (2x 20 mL) and brine (20 mL). The resulting crude was purified over SiO_2 using a gradient of 60% c-hexane in EtOAc towards EtOAc to yield 250 mg (0.77 mmol, 87%) of the title compound as a white solid. ^{1}H NMR (500 MHz, Chloroform-d) δ 7.99 (d, *J* = 2.4 Hz, 1H), 7.67 (dd, *J* = 8.6, 1.9 Hz, 1H), 6.92 (d, *J* = 8.8 Hz, 1H), 5.02 (hept, *J* = 6.6 Hz, 1H), 3.93 (s, 3H), 2.85 (t, *J* = 8.5 Hz, 2H), 2.55 (t, *J* = 8.5 Hz, 2H), 1.27 (d, *J* = 6.7 Hz, 6H). ^{13}C NMR (126 MHz, Chloroform-d) δ 164.5, 156.8, 148.2, 130.8, 130.0, 126.2, 112.0, 111.4, 56.4, 46.5, 27.4, 22.1, 20.4. LC-MS (ESI) *m*/*z* found: 325 $[M+H]^+$; retention time: 4.76 minutes. HRMS-ESI $[M+H]^+$ calculated for $C_{14}H_{18}BrN_2O_2$: 325.0546, found: 325.0531.

2-isopropyl-6-(4-methoxy-3-(pyridin-3-yl)phenyl)-4,5-dihydropyridazin-3(2H)-one (**104**)

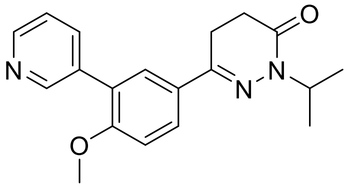

Dihydropyridazinone **103** (100 mg, 0.31 mmol) and pyridin-3-ylboronic acid (45 mg, 0.37 mmol) were charged to a microwave vial after which DME (3 mL) and 1M Na_2CO_3 (1.0 mL, 1.0 mmol) were added. The mixture was degassed with N_2 for 5 m after which Pd(dppf)Cl_2 (25 mg, 0.03 mmol) was added. The reaction was heated in the icrowave for 1 h at 120°C. The reaction mixture was diluted with MTBE and filtered over Celite. The residue was washed with saturated $NaHCO_3$ (2x) and brine (1x). The organic phase was dried over Na_2SO_4, filtered and concentrated *in vacuo* to be further purified over SiO_2 using a gradient of 50% EtOAc in heptane towards 100% EtOAc to yield 63 mg (0.20 mmol, 63%) of the title compound. ^{1}H NMR (500 MHz, Chloroform-d) δ 9.27 – 8.58 (m, 2H), 7.92 (d, J = 7.0 Hz, 1H), 7.79 (d, J = 9.7 Hz, 2H), 7.48 (s, 1H), 7.06 (d, J = 8.5 Hz, 1H), 5.05 (hept, J = 6.4 Hz, 1H), 3.89 (s, 3H), 2.93 (t, J = 8.1 Hz, 2H), 2.60 (t, J = 8.1 Hz, 2H), 1.29 (d, J = 6.6 Hz, 6H). ^{13}C NMR (126 MHz, Chloroform-d) δ 164.6, 157.7, 149.8, 149.3, 147.9, 137.1, 134.0, 129.1, 128.3, 127.3, 127.1, 123.2, 111.1, 55.8, 46.5, 27.5, 22.3, 20.4. LC-MS (ESI) m/z found: 324 $[M+H]^+$; retention time: 3.37 minutes. HRMS-ESI $[M+H]^+$ calculated for $C_{19}H_{22}N_3O_2$: 324.1707, found: 324.1702.

3-(3-bromo-4-methoxyphenyl)-4,4-dimethyl-1H-pyrazole-5(4H)-thione (**106**)

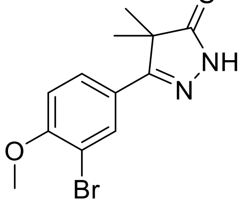

Dihydropyrazolone **105** (1.0 g, 3.4 mmol) was added to toluene (10 mL) and Lawessons reagent (1.6 g, 4.0 mmol). The mixture was refluxed for 18 h after which the reaction was cooled to rt. The resulting solids were collected and washed with cyclohexane (20

mL), yielding 0.73 g (2.3 mmol, 69%) of the title compound as a white solid. ^{1}H NMR (500 MHz, Chloroform-d) δ 10.47 (s, 1H), 8.13 (d, *J* = 2.2 Hz, 1H), 7.81 (dd, *J* = 8.7, 2.2 Hz, 1H), 6.96 (d, *J* = 8.7 Hz, 1H), 3.97 (s, 3H), 1.59 (s, 6H). ^{13}C NMR (126 MHz, Chloroform-d) δ 207.6, 169.1, 157.7, 131.9, 127.3, 124.1, 112.4, 111.7, 60.2, 56.4, 26.5. LC-MS (ESI) *m*/*z* found: 313 $[M+H]^+$; retention time: 4.55 minutes.

3-(3-bromo-4-methoxyphenyl)-5-hydrazono-4,4-dimethyl-4,5-dihydro-1H-pyrazole (**107**)

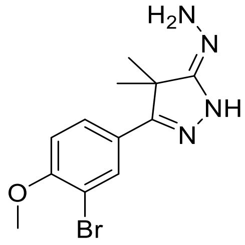

Dihydropyrazolethione **106** (0.5 g, 1.60 mmol) was dissolved in ethanol (40 mL) and hydrazine hydrate (0.90 ml, 16.0 mmol) was added. The mixture was heated at 70°C for 3 h, after which it was cooled town to room temperature and water (40 mL) was added. The precipitate was collected and dried *in vacuo*, yielding 0.33 g (1.06 mmol, 66%) of the title compound as a white solid. ^{1}H NMR (500 MHz, Chloroform-d) δ 8.12 (d, *J* = 2.1 Hz, 1H), 8.03 (d, *J* = 2.1 Hz, 1H), 7.80 (dd, *J* = 8.7, 2.1 Hz, 1H), 7.69 (dd, *J* = 8.5, 2.0 Hz, 1H), 6.94 (d, *J* = 8.8 Hz, 1H), 6.90 (d, *J* = 8.7 Hz, 1H), 3.95 (s, 1H), 3.92 (s, 3H), 1.56 (s, 1H), 1.52 (s, 6H). The peaks at 3.95 and 1.56 are contributed to the other possible isomer. LC-MS (ESI) *m*/*z* found: 311 $[M+H]^+$; retention time: 2.95 minutes.

6-(3-bromo-4-methoxyphenyl)-3-cyclopropyl-7,7-dimethyl-7H-pyrazolo[5,1-c][1,2,4]triazole (**108**)

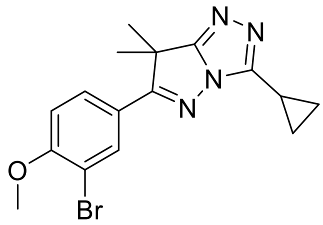

Hydrazono-dihydropyrazole **107** (200 mg, 0.64 mmol) was dissolved in pyridine (2 ml) and cyclopropanecarbonyl chloride (0.12 ml, 1.28 mmol) was added. The

mixture was heated to 70°C for 2 hours. Subsequently the mixture was diluted with DMF (2 mL) and heated to 150°C for 2 hours. The mixture was diluted with DCM (40 mL), extracted with $NaHCO_3$ (2x 40 mL) and brine (40 mL). The organic layer was dried over Na2SO4, concentrated *in vacuo* and the resulting crude was purified over SiO_2 using a gradient of 30% EtOAc in n-heptane towards 100% EtOAc, yielding 110 mg (0.31 mmol, 47%) of the title compound as a light brown solid. ^{1}H NMR (500 MHz, Chloroform-d) δ 8.19 (s, 1H), 7.86 (d, *J* = 8.7 Hz, 1H), 6.99 (d, *J* = 8.6 Hz, 1H), 3.98 (s, 3H), 2.33 – 2.23 (m, 1H), 1.75 (s, 6H), 1.36 – 1.29 (m, 2H), 1.18 – 1.11 (m, 2H). ^{13}C NMR (126 MHz, Chloroform-d) δ 175.6, 162.2, 158.6, 148.7, 132.7, 128.3, 123.0, 112.6, 111.7, 56.5, 45.2, 23.9, 7.9, 5.9. LC-MS (ESI) *m/z* found: 361 $[M+H]^+$; retention time: 4.66 minutes, purity = 90% @ 254 nM.

3-cyclopropyl-6-(4-methoxy-3-(pyridin-3-yl)phenyl)-7,7-dimethyl-7H-pyrazolo[5,1-c][1,2,4]triazole (**109**)

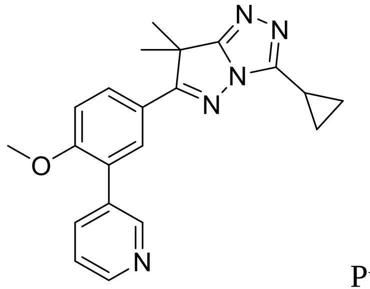

Pyrazolotriazole **108** (90 mg, 0.25 mmol) and pyridin-3-ylboronic acid (46 mg, 0.37 mmol) were charged to a microwave vial after which DME (3 mL) and 1M Na_2CO_3 (0.8 mL, 0.8 mmol) were added. The mixture was degassed with N_2 for 5 m after which $Pd(dppf)Cl_2$ (20 mg, 0.03 mmol) was added. The reaction was heated in the microwave for 1 h at 120°C. The reaction mixture was diluted with EtOAc (30 mL) and filtered over Celite. The residue was washed with saturated $NaHCO_3$ (2x 20 mL) and brine (20 mL). The organic phase was dried over Na_2SO_4, filtered and concentrated *in vacuo* to be further purified over SiO_2 using a gradient of 50% EtOAc in heptane towards 4% MeOH in EtOAc to yield 27 mg (0.08 mmol, 30%) of the title compound. ^{1}H NMR (600 MHz, Chloroform-d) δ 8.80 (s, 1H), 8.63 (s, 1H), 8.00 – 7.91 (m, 2H), 7.88 (d, *J* = 7.6 Hz, 1H), 7.44 – 7.35 (m, 1H), 7.10 (d, *J* =

8.6 Hz, 1H), 3.91 (s, 3H), 2.32 – 2.21 (m, 1H), 1.78 (s, 6H), 1.33 – 1.27 (m, 2H), 1.16 – 1.06 (m, 2H). ^{13}C NMR (150 MHz, Chloroform-d) δ 177.5, 176.3, 159.3, 150.0, 148.5, 138.3, 136.9, 133.2, 130.2, 129.4, 129.2, 128.1, 122.3, 111.3, 55.9, 45.1, 24.0, 7.8, 5.9. LC-MS (ESI) *m*/*z* found: 360 $[M+H]^+$; retention time: 3.63 minutes. HRMS-ESI $[M+H]^+$ calculated for $C_{21}H_{22}N_5O$: 360.1819, found: 360.1833.

2-oxo-2-phenylethyl 3-bromo-4-methoxybenzoate (**111**)

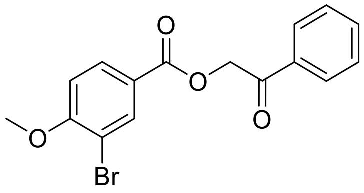

Benzoic acid **75** (1.0 g, 4.3 mmol) was added to round bottom flask and DMF (20 ml) was added, followed by sodium hydride (60% in mineral oil) (0.18 g, 4.5 mmol). The mixture was stirred for 15 m after which 2-bromo-1-phenylethanone (0.86 g, 4.3 mmol) was added. The reaction mixture was stirred for another 3 hours after which it was quenched with water and extracted with MTBE (50 mL). The organic layer was washed with brine and dried over $MgSO_4$. Volatiles were evaporated and the resulting crude was recrystallized from EtOH. Resulting crystals were washed with 10 mL of EtOH yielding 1.25 g (3.6 mmol, 83%) of the title compound. ^{1}H NMR (300 MHz, Chloroform-d) δ 8.34 (d, *J* = 2.1 Hz, 1H), 8.09 (dd, *J* = 8.6, 2.1 Hz, 1H), 7.99 – 7.92 (m, 2H), 7.67 – 7.59 (m, 1H), 7.56 – 7.47 (m, 2H), 6.95 (d, *J* = 8.7 Hz, 1H), 5.56 (s, 2H), 3.98 (s, 3H). LC-MS (ESI) *m*/*z* found: no mass observed; retention time: 4.89 minutes.

2-(3-fluorophenyl)-2-oxoethyl 3-bromo-4-methoxybenzoate (**112**)

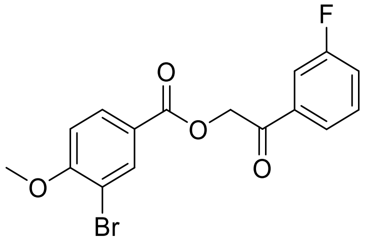

Benzoic acid **75** (1.0 g, 4.3 mmol) was added to round bottom flask and DMF (20 ml) was added, followed by sodium hydride (60% in mineral oil) (0.18 g, 4.5 mmol). The mixture was stirred for 15 m after which 2-bromo-1-(3-fluorophenyl)ethanone (0.94 g, 4.3 mmol) was added. The reaction mixture was stirred for another 3 hours after which it was quenched with water and extracted with MTBE (50 mL). The organic layer was washed with brine and dried over $MgSO_4$. Volatiles were evaporated yielding 1.49 g (4.1 mmol, 94%) of the title compound. ^{1}H NMR (600 MHz, Chloroform-d) δ 8.36 – 8.27 (m, 1H), 8.08 (dd, *J* = 8.7, 2.0 Hz, 1H), 7.74 (d, *J* = 7.8 Hz, 1H), 7.68 – 7.61 (m, 1H), 7.55 – 7.45 (m, 1H), 7.33 (td, *J* = 9.2, 8.7, 1.8 Hz, 1H), 6.95 (d, *J* = 8.7 Hz, 1H), 5.52 (s, 2H), 3.98 (s, 3H), 1.55 (s, 3H). LC-MS (ESI) *m*/*z* found: no mass observed; retention time: 4.97 minutes.

2-(4-fluorophenyl)-2-oxoethyl 3-bromo-4-methoxybenzoate (**113**)

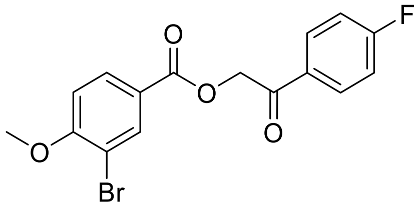

Benzoic acid **75** (1.0 g, 4.3 mmol) was added to round bottom flask and DMF (20 ml) was added, followed by sodium hydride (60% in mineral oil) (0.18 g, 4.5 mmol). The mixture was stirred for 15 m after which 2-bromo-1-(4-fluorophenyl)ethanone (0.94 g, 4.3 mmol) was added. The reaction mixture was stirred for another 3 hours after which it was quenched with water and extracted with MTBE (50 mL). The organic layer was washed with brine and dried over $MgSO_4$. Volatiles were evaporated and the resulting crude was recrystallized from EtOH. Resulting crystals were washed with 10 mL of EtOH yielding 1.35

g (3.7 mmol, 85%) of the title compound. ^{1}H NMR (600 MHz, Chloroform-d) δ 8.36 – 8.30 (m, 1H), 8.08 (d, *J* = 8.6 Hz, 1H), 8.03 – 7.95 (m, 2H), 7.19 (t, *J* = 7.9 Hz, 2H), 6.95 (d, *J* = 8.6 Hz, 1H), 5.52 (s, 2H), 3.98 (s, 3H). ^{13}C NMR (150 MHz, Chloroform-d) δ 190.6, 166.2 (d, *J* = 256 Hz), 164.6, 159.9, 135.2, 131.1, 130.6 (d, *J* = 4 Hz),130.5 (d, *J* = 9 Hz), 122.8, 116.2 (d, *J* = 22 Hz), 111.6, 111.1, 66.3, 56.5. LC-MS (ESI) *m*/*z* found: no mass observed; retention time: 4.95 minutes. HRMS-ESI $[M+H]^+$ calculated for $C_{16}H_{12}BrFO_4$: 365.9903, found: 388.9795 $[M+Na]^+$.

1-oxo-1-phenylpropan-2-yl 3-bromo-4-methoxybenzoate (**114**)

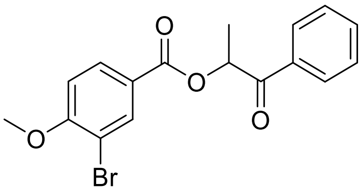

Benzoic acid **75** (1.0 g, 4.3 mmol) was added to round bottom flask and DMF (20 ml) was added, followed by sodium hydride (60% in mineral oil) (0.18 g, 4.5 mmol). The mixture was stirred for 15 m after which 2-bromo-1-phenylpropan-1-one (0.92 g, 4.3 mmol) was added. The reaction mixture was stirred for another 3 hours after which it was quenched with water and extracted with EtOAc (50 mL). The organic layer was washed with brine and dried over $MgSO_4$. Volatiles were evaporated yielding 1.35 g (3.7 mmol, 85%) of the title compound as an off-white solid. ^{1}H NMR (300 MHz, Chloroform-d) δ 8.29 (d, *J* = 2.0 Hz, 1H), 8.06 – 7.96 (m, 3H), 7.60 (t, 1H), 7.54 – 7.44 (m, 2H), 6.92 (d, *J* = 8.7 Hz, 1H), 6.18 (q, *J* = 7.0 Hz, 1H), 3.96 (s, 3H), 1.67 (d, *J* = 7.0 Hz, 3H). LC-MS (ESI) *m*/*z* found: no mass observed; retention time: 5.01 minutes.

2-(3-bromo-4-methoxyphenyl)-4-phenyloxazole (**116**)

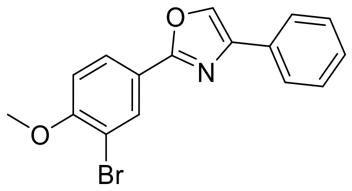

Keto-ester **112** (500 mg, 1.43 mmol) was added to a microwave tube, followed by ammonium acetate (552 mg, 7.16 mmol) and acetic acid (5 mL). The mixture was heated in the microwave at 170°C for 6 hours, after which the reaction mixture was diluted with EtOAc and washed with sat. aq. Na_2CO_3 (2x 50 mL) and brine (50 mL). The organic layer was dried over $MgSO_4$ and volatiles were evaporated. The resulting crude was purified over SiO_2 using a gradient of 50% heptane in EtOAc towards 5% MeOH in EtOAc yielding 50 mg (0.15 mmol, 11%) of the title compound as a white solid. ^{1}H NMR (600 MHz, Chloroform-d) δ 8.35 (d, J = 2.0 Hz, 1H), 8.07 (dd, J = 8.6, 2.0 Hz, 1H), 7.96 (s, 1H), 7.83 (d, J = 7.3 Hz, 2H), 7.46 (t, J = 7.7 Hz, 2H), 7.36 (t, J = 7.4 Hz, 1H), 7.01 (d, J = 8.6 Hz, 1H), 3.99 (s, 3H). ^{13}C NMR (150 MHz, Chloroform-d) δ 160.6, 157.6, 142.0, 133.3, 131.6, 131.0, 128.8, 128.2, 127.1, 125.6, 121.5, 112.1, 111.7, 56.4. LC-MS (ESI) *m/z* found: 330 $[M+H]^+$; retention time: 5.58 minutes. HRMS-ESI $[M+H]^+$ calculated for $C_{16}H_{13}BrNO_2$: 330.0124, found: 330.0112.

2-(3-bromo-4-methoxyphenyl)-4-(3-fluorophenyl)oxazole) MSMTcr676) (**117**)

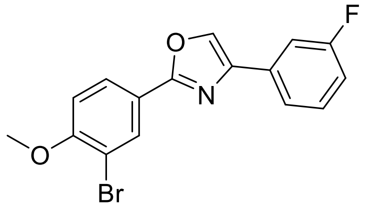

Keto-ester **113** (500 mg, 1.36 mmol) was added to a microwave tube, followed by ammonium acetate (525 mg, 6.81 mmol) and acetic acid (5 mL). The mixture was heated in the microwave at 170°C for 6 hours, after which the reaction mixture was diluted with EtOAc and washed with sat. aq. Na_2CO_3 (2x 50 mL) and brine (50 mL). The organic layer was dried over $MgSO_4$ and volatiles were evaporated. The resulting crude was purified over SiO_2 using a gradient of 50% heptane in EtOAc towards 5% MeOH in EtOAc yielding 55 mg

(0.16 mmol, 12%) of the title compound as a white solid. ^{1}H NMR (600 MHz, Chloroform-d) δ 8.34 (s, 1H), 8.06 (d, *J* = 8.6 Hz, 1H), 7.97 (s, 1H), 7.59 (d, *J* = 7.7 Hz, 1H), 7.56 (d, *J* = 9.9 Hz, 1H), 7.41 (q, *J* = 7.5 Hz, 1H), 7.05 (t, *J* = 8.4 Hz, 1H), 7.01 (d, *J* = 8.6 Hz, 1H), 4.00 (s, 3H). ^{13}C NMR (150 MHz, Chloroform-d) δ 164.0, 162.4, 160.8, 157.7, 141.0, 141.0, 133.8, 133.2, 133.2, 131.6, 130.3, 130.3, 127.1, 121.2, 121.2, 121.2, 115.0, 114.9, 112.7, 112.6, 112.1, 111.8, 56.4. LC-MS (ESI) *m/z* found: 348 [M+H]$^{+}$; retention time: 5.66 minutes. HRMS-ESI [M+H]$^{+}$ calculated for $C_{16}H_{12}BrFNO_2$: 348.0030, found: 348.0022.

2-(3-bromo-4-methoxyphenyl)-4-(4-fluorophenyl)oxazole (**118**)

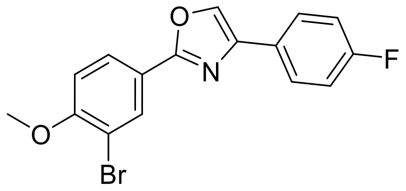

Keto-ester **117** (500 mg, 1.36 mmol) was added to a microwave tube, followed by ammonium acetate (525 mg, 6.81 mmol) and acetic acid (5 mL). The mixture was heated in the microwave at 170°C for 6 hours, after which the reaction mixture was diluted with EtOAc and washed with sat. aq. Na_2CO_3 (2x 50 mL) and brine (50 mL). The organic layer was dried over $MgSO_4$ and volatiles were evaporated. The resulting crude was purified over SiO_2 using a gradient of 50% heptane in EtOAc towards 5% MeOH in EtOAc yielding 140 mg (0.40 mmol, 30%) of the title compound as a white solid. ^{1}H NMR (600 MHz, Chloroform-*d*) δ 8.31 (d, *J* = 2.0 Hz, 1H), 8.03 (dd, *J* = 8.6, 2.1 Hz, 1H), 7.89 (s, 1H), 7.78 (dd, *J* = 8.6, 5.5 Hz, 2H), 7.12 (t, *J* = 8.7 Hz, 2H), 6.99 (d, *J* = 8.6 Hz, 1H), 3.97 (s, 3H). ^{13}C NMR (151 MHz, Chloroform-*d*) δ 163.5, 161.8, 160.7, 157.6, 141.2, 132.9, 131.6, 127.4, 127.4, 127.2, 127.2, 127.1, 121.4, 115.8, 115.7, 112.1, 111.8, 56.4. LC-MS (ESI) *m/z* found: 348 [M+H]$^{+}$; retention time: 6.28 minutes. HRMS-ESI [M+H]$^{+}$ calculated for $C_{16}H_{12}BrFNO_2$: 348.0030, found: 348.0035.

2-(3-bromo-4-methoxyphenyl)-5-methyl-4-phenyloxazole (**119**)

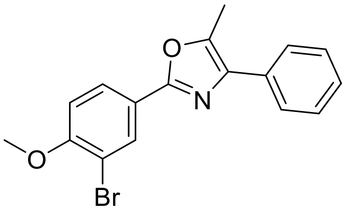

Keto-ester **118** (500 mg, 1.43 mmol) was added to a microwave tube, followed by ammonium acetate (531 mg, 6.88 mmol) and acetic acid (5 mL). The mixture was heated in the microwave at 170°C for 6 hours, after which the reaction mixture was diluted with EtOAc and washed with sat. aq. Na_2CO_3 (2x 50 mL) and brine (50 mL). The organic layer was dried over $MgSO_4$ and volatiles were evaporated. The resulting crude was purified over SiO_2 using a gradient of 50% heptane in EtOAc towards 5% MeOH in EtOAc yielding 105 mg (0.31 mmol, 22%) of the title compound as a white solid. ^{1}H NMR (600 MHz, Chloroform-d) δ 8.30 (d, *J* = 1.9 Hz, 1H), 8.02 (dd, *J* = 8.6, 1.5 Hz, 1H), 7.74 (d, *J* = 7.6 Hz, 2H), 7.47 (t, *J* = 7.7 Hz, 2H), 7.35 (t, *J* = 7.4 Hz, 1H), 6.99 (d, *J* = 8.6 Hz, 1H), 3.98 (s, 3H), 2.62 (s, 3H). ^{13}C NMR (150 MHz, Chloroform-d) δ 158.1, 157.2, 143.9, 135.9, 132.3, 131.2, 128.6, 127.4, 126.8, 126.6, 121.7, 112.0, 111.7, 56.4, 12.0. LC-MS (ESI) *m/z* found: 344 $[M+H]^+$; retention time: 5.74 minutes. HRMS-ESI $[M+H]^+$ calculated for $C_{17}H_{15}BrNO_2$: 344.0281, found: 344.0273.

2-(4-methoxy-3-(pyridin-3-yl)phenyl)-4-phenyloxazole (**121**)

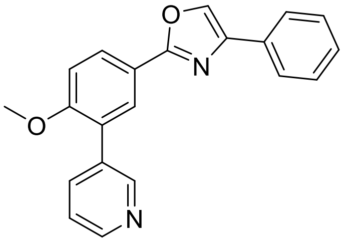

Oxazole **116** (50 mg, 0.15 mmol) and pyridin-3-ylboronic acid (28 mg, 0.23 mmol) were charged to a microwave vial after which DME (3 mL) and 1M Na_2CO_3 (0.6 mL, 0.6 mmol) were added. The mixture was degassed with N_2 for 5 m after which $Pd(dppf)Cl_2$ (12 mg, 0.02 mmol) was added. The reaction was heated in the microwave for 1 h

at 120°C. The reaction mixture was diluted with EtOAc (30 mL) and filtered over Celite. The residue was washed with saturated $NaHCO_3$ (2x 20 mL) and brine (20 mL). The organic phase was dried over Na_2SO_4, filtered and concentrated *in vacuo* to be further purified over SiO_2 using a gradient of 30% EtOAc in heptane towards 100% EtOAc to yield 18 mg (0.06 mmol, 36%) of the title compound. ^{1}H NMR (600 MHz, Chloroform-d) δ 8.86 (d, 1H), 8.61 (dd, 1H), 8.14 (dd, *J* = 8.6, 2.1 Hz, 1H), 8.10 (d, *J* = 2.1 Hz, 1H), 7.98 – 7.94 (m, 2H), 7.82 (d, *J* = 7.4 Hz, 2H), 7.45 – 7.39 (m, 3H), 7.33 (t, *J* = 7.4 Hz, 1H), 7.11 (d, *J* = 8.6 Hz, 1H), 3.91 (s, 3H). ^{13}C NMR (150 MHz, Chloroform-d) δ 161.5, 158.3, 149.7, 147.6, 142.0, 137.5, 133.8, 133.2, 131.1, 129.0, 128.8, 128.2, 128.1, 127.2, 125.6, 123.2, 120.8, 111.4, 55.8. LC-MS (ESI) *m*/*z* found: 329 $[M+H]^+$; retention time: 4.15 minutes. HRMS-ESI $[M+H]^+$ calculated for $C_{21}H_{17}N_2O_2$: 329.1285, found: 329.1278.

4-(3-fluorophenyl)-2-(4-methoxy-3-(pyridin-3-yl)phenyl)oxazole (**122**)

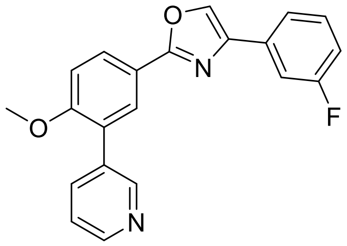

Oxazole **117** (50 mg, 0.14 mmol) and pyridin-3-ylboronic acid (27 mg, 0.22 mmol) were charged to a microwave vial after which DME (3 mL) and 1M Na_2CO_3 (0.6 mL, 0.6 mmol) were added. The mixture was degassed with N_2 for 5 m after which Pd(dppf)Cl_2 (12 mg, 0.01 mmol) was added. The reaction was heated in the microwave for 1 h at 120°C. The reaction mixture was diluted with EtOAc (30 mL) and filtered over Celite. The residue was washed with saturated $NaHCO_3$ (2x 20 mL) and brine (20 mL). The organic phase was dried over Na_2SO_4, filtered and concentrated *in vacuo* to be further purified over SiO_2 using a gradient of 20% EtOAc in c-hexane towards 70% EtOAc in c-hexane to yield 37 mg (0.11 mmol, 74%) of the title compound. ^{1}H NMR (600 MHz, Chloroform-d) δ 8.84 (s, 1H), 8.61 (d, *J* = 4.0 Hz, 1H), 8.12 (dd, *J* = 8.6, 2.0 Hz, 1H), 8.08 (d, *J* = 1.9 Hz, 1H), 7.95 (s, 1H),

7.92 (d, *J* = 7.8 Hz, 1H), 7.57 (d, *J* = 7.7 Hz, 1H), 7.54 (d, *J* = 9.8 Hz, 1H), 7.39 (dd, *J* = 7.8, 5.5 Hz, 2H), 7.10 (d, *J* = 8.6 Hz, 1H), 7.02 (td, *J* = 8.4, 2.3 Hz, 1H), 3.90 (s, 3H). ^{13}C NMR (150 MHz, Chloroform-d) δ 163.2 (d, *J* = 245 Hz), 161.7, 158.4, 150.1, 148.2, 141.0, 137.0, 133.6, 133.5, 133.4 (d, *J* = 9 Hz), 130.3 (d, *J* = 9 Hz), 129.1, 128.1, 127.5, 123.1, 121.2 (d, *J* = 3Hz), 120.5, 114.9 (d, *J* = 22 Hz), 112.6 (d, *J* = 23 Hz), 111.4, 55.8. LC-MS (ESI) *m*/*z* found: 347 $[M+H]^+$; retention time: 4.30 minutes. HRMS-ESI $[M+H]^+$ calculated for $C_{21}H_{16}FN_2O_2$: 347.1190, found: 347.1181.

4-(4-fluorophenyl)-2-(4-methoxy-3-(pyridin-3-yl)phenyl)oxazole (**123**)

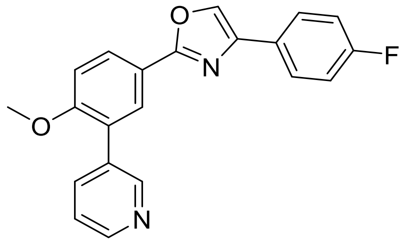

Oxazole **118** (90 mg, 0.26 mmol) and pyridin-3-ylboronic acid (45 mg, 0.36 mmol) were charged to a microwave vial after which DME (3 mL) and 1M Na_2CO_3 (0.8 mL, 0.8 mmol) were added. The mixture was degassed with N_2 for 5 m after which Pd(dppf)Cl_2 (21 mg, 0.03 mmol) was added. The reaction was heated in the microwave for 1 h at 120°C. The reaction mixture was diluted with EtOAc (30 mL) and filtered over Celite. The residue was washed with saturated $NaHCO_3$ (2x 20 mL) and brine (20 mL). The organic phase was dried over Na_2SO_4, filtered and concentrated *in vacuo* to be further purified over SiO_2 using a gradient of 30% EtOAc in heptane towards 100% EtOAc to yield 59 mg (0.17 mmol, 66%) of the title compound. ^{1}H NMR (500 MHz, Chloroform-*d*) δ 8.85 (s, 1H), 8.61 (s, 1H), 8.15 – 8.05 (m, 2H), 7.95 – 7.87 (m, 2H), 7.82 – 7.74 (m, 2H), 7.38 (s, 1H), 7.16 – 7.06 (m, 3H), 3.90 (s, 3H). ^{13}C NMR (126 MHz, Chloroform-*d*) δ 163.6, 161.6 (d, 3.5 Hz), 158.3, 149.2 (d, 247.1 Hz), 141.1, 136.9, 133.5, 132.7, 129.0, 128.0, 127.5, 127.4 (d, 8.1 Hz), 123.1, 120.6, 115.7 (d, 21.6 Hz), 111.3, 55.8. LC-MS (ESI) *m*/*z* found: 347 $[M+H]^+$; retention time: 4.25 minutes. HRMS-ESI $[M+H]^+$ calculated for $C_{21}H_{16}FN_2O_2$: 347.1190, found: 347.1189.

2-(4-methoxy-3-(pyridin-3-yl)phenyl)-5-methyl-4-phenyloxazole (**124**)

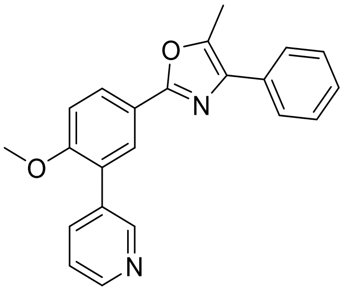

Oxazole **119** (100 mg, 0.29 mmol) and pyridin-3-ylboronic acid (54 mg, 0.44 mmol) were charged to a microwave vial after which DME (3 mL) and 1M Na_2CO_3 (1.1 mL, 1.1 mmol) were added. The mixture was degassed with N_2 for 5 m after which Pd(dppf)Cl_2 (24 mg, 0.03 mmol) was added. The reaction was heated in the microwave for 1 h at 120°C. The reaction mixture was diluted with EtOAc (30 mL) and filtered over Celite. The residue was washed with saturated $NaHCO_3$ (2x 20 mL) and brine (20 mL). The organic phase was dried over Na_2SO_4, filtered and concentrated *in vacuo* to be further purified over SiO_2 using a gradient of 20% EtOAc in c-hexane towards 70% EtOAc in c-hexane to yield 45 mg (0.13 mmol, 45%) of the title compound. ^{1}H NMR (600 MHz, Chloroform-d) δ 8.85 (s, 1H), 8.60 (d, *J* = 4.6 Hz, 1H), 8.10 (dd, *J* = 8.6, 2.0 Hz, 1H), 8.05 (d, *J* = 2.0 Hz, 1H), 7.97 (d, *J* = 7.8 Hz, 1H), 7.73 (d, *J* = 7.6 Hz, 2H), 7.47 – 7.39 (m, 3H), 7.32 (t, *J* = 7.4 Hz, 1H), 7.09 (d, *J* = 8.6 Hz, 1H), 3.90 (s, 3H), 2.61 (s, 3H). ^{13}C NMR (150 MHz, Chloroform-d) δ 158.9, 157.9, 149.6, 147.4, 143.7, 137.6, 135.9, 133.9, 132.4, 128.6, 128.6, 127.8, 127.3, 127.1, 126.8, 123.3, 121.0, 111.4, 55.8, 12.0. LC-MS (ESI) *m/z* found: 343 $[M+H]^+$; retention time: 4.29 minutes. HRMS-ESI $[M+H]^+$ calculated for $C_{22}H_{19}N_2O_2$: 343.1441, found: 3343.1433.

2-(3-bromo-4-methoxyphenyl)-5-cyclopentyl-1,3,4-oxadiazole (**128**)

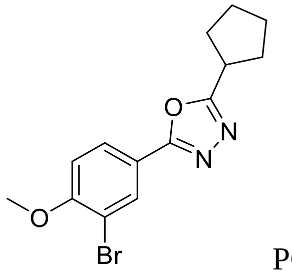

$POCl_3$ (2.0 ml, 21.5 mmol) was added to hydrazide **126** (400 mg, 1.63 mmol) and cyclopentanecarboxylic acid (186 mg, 1.63 mmol) after which the mixture was refluxed for 1 hour. The reaction was quenched by pouring it over crushed ice after which the resulting solids were collected and dried *in vacuo* to yield 420 mg (1.30 mmol, 80%) of the title compound as a white solid. ^1H NMR (500 MHz, Chloroform-d) δ 8.18 (d, *J* = 2.1 Hz, 1H), 7.96 (dd, *J* = 8.6, 2.1 Hz, 1H), 6.98 (d, *J* = 8.6 Hz, 1H), 4.01 – 3.93 (m, 5H), 3.40 (td, *J* = 11.9, 2.0 Hz, 2H), 2.18 – 2.08 (m, 1H), 1.70 (dd, *J* = 13.0, 2.1 Hz, 2H), 1.46 (dtd, *J* = 13.3, 11.8, 4.5 Hz, 2H). ^{13}C NMR (126 MHz, Chloroform-d) δ 165.2, 163.6, 158.4, 131.7, 127.5, 117.7, 112.3, 111.9, 67.6, 56.5, 33.5, 32.6, 32.4. LC-MS (ESI) *m/z* found: 353 $[M+H]^+$; retention time: 4.15 minutes.

2-(3-bromo-4-methoxyphenyl)-5-((tetrahydro-2H-pyran-4-yl)methyl)-1,3,4-oxadiazole (**129**)

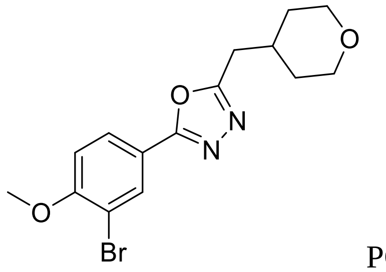

$POCl_3$ (2.0 ml, 21.5 mmol) was added to hydrazide **126** (400 mg, 1.63 mmol) and 2-(tetrahydro-2H-pyran-4-yl)acetic acid (235 mg, 1.63 mmol) after which the mixture was refluxed for 1 h. The reaction was quenched by pouring it over crushed ice after which the resulting solids were collected and dried *in vacuo* to yield 310 mg (0.88 mmol, 54%) of the title compound as a white solid. ^1H NMR (600 MHz, Chloroform-d) δ 8.18 (d, 1H), 7.96 (dd, *J* = 8.6, 1.8 Hz, 1H), 6.98 (d, *J* = 8.6 Hz, 1H), 3.96 (s, 3H), 3.36 (p, *J* = 8.1 Hz, 1H), 2.18

– 2.10 (m, 2H), 2.02 – 1.93 (m, 2H), 1.89 – 1.80 (m, 2H), 1.77 – 1.68 (m, 2H). ^{13}C NMR (150 MHz, Chloroform-d) δ 170.0, 163.3, 158.2, 131.6, 127.4, 117.9, 112.2, 111.9, 56.4, 36.0, 31.1, 26.9, 25.5. LC-MS (ESI) *m*/*z* found: 323 $[M+H]^+$; retention time: 4.89 minutes.

2-(3-bromo-4-methoxyphenyl)-5-phenyl-1,3,4-oxadiazole (**130**)

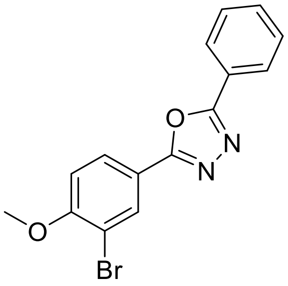

$POCl_3$ (2.0 ml, 21.5 mmol) was added to hydrazide **126** (400 mg, 1.63 mmol) and benzoic acid (149 mg, 1.22 mmol) after which the mixture was refluxed for 1 h. The reaction was quenched by pouring it over crushed ice after which the resulting solids were collected and dried *in vacuo* to yield 230 mg (0.49 mmol, 40%) of the title compound in ~70% purity (based on ^{1}H-NMR; other 30% is benzoic acid). Used in the next step without further purification. ^{1}H NMR (300 MHz, DMSO-d_6) δ 8.31 (d, *J* = 2.1 Hz, 1H), 8.20 – 8.10 (m, 3H), 7.69 – 7.58 (m, 3H), 7.36 (d, *J* = 8.7 Hz, 1H), 3.97 (s, 3H). LC-MS (ESI) *m*/*z* found: 331 $[M+H]^+$; retention time: 5.00 minutes.

2-(3-bromo-4-methoxyphenyl)-5-(3-fluorophenyl)-1,3,4-oxadiazole (**131**)

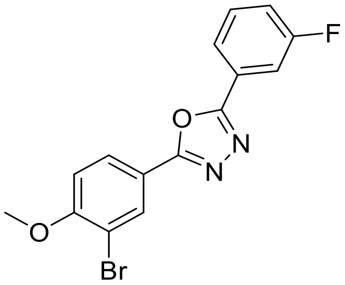

$POCl_3$ (2.0 ml, 21.5 mmol) was added to hydrazide **126** (300 mg, 1.22 mmol) and 3-fluorobenzoic acid (172 mg, 1.22 mmol) after which the mixture was refluxed for 1 hour. The reaction was quenched by pouring it over crushed ice after which the resulting solids were purified over SiO_2 using a gradient of 10% EtOAc in c-hexane towards

60% EtOAc in c-hexane yielding 300 mg (0.71 mmol, 58%) of the title compound as a white solid in 82% purity (based LC-MS). Used in the next step without further purification. ^{1}H NMR (300 MHz, DMSO-d$_6$) δ 8.39 – 8.30 (m, 1H), 8.15 (d, *J* = 8.2 Hz, 1H), 8.00 (d, *J* = 8.1 Hz, 2H), 7.68 (q, *J* = 7.4 Hz, 1H), 7.59 – 7.45 (m, 1H), 7.35 (d, *J* = 8.6 Hz, 1H), 3.97 (s, 3H). LC-MS (ESI) *m/z* found: 349 $[M+H]^+$; retention time: 5.10 minutes, purity: 82% @ 254 nM.

2-cyclopentyl-5-(4-methoxy-3-(pyridin-3-yl)phenyl)-1,3,4-oxadiazole (**133**)

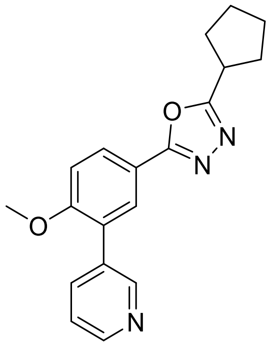

Oxadiazole **128** (100 mg, 0.31 mmol) and pyridin-3-ylboronic acid (49 mg, 0.40 mmol) were charged to a microwave vial after which DME (3 mL) and 1M Na_2CO_3 (1.0 mL, 1.0 mmol) were added. The mixture was degassed with N_2 for 5 m after which Pd(dppf)Cl_2 (13 mg, 0.02 mmol) was added. The reaction was heated in the microwave for 1 h at 120°C. The reaction mixture was diluted with EtOAc (30 mL) and filtered over Celite. The residue was washed with saturated $NaHCO_3$ (2x 20 mL) and brine (20 mL). The organic phase was dried over Na_2SO_4, filtered and concentrated *in vacuo* to be further purified over SiO_2 using a gradient of 40% EtOAc in c-hexane towards 100% EtOAc to yield 49 mg (0.15 mmol, 49%) of the title compound. ^{1}H NMR (500 MHz, Chloroform-d) δ 8.84 (s, 1H), 8.63 (s, 1H), 8.05 (dd, *J* = 8.6, 1.9 Hz, 1H), 7.96 (d, *J* = 1.9 Hz, 1H), 7.88 (d, *J* = 7.7 Hz, 1H), 7.42 – 7.34 (m, 1H), 7.10 (d, *J* = 8.7 Hz, 1H), 3.89 (s, 3H), 3.37 (p, *J* = 8.0 Hz, 1H), 2.18 – 2.10 (m, 2H), 2.02 – 1.94 (m, 2H), 1.88 – 1.80 (m, 2H), 1.77 – 1.67 (m, 2H). ^{13}C NMR (126 MHz, Chloroform-d) δ 169.9, 164.2, 159.0, 150.1, 148.3, 136.9, 133.3, 129.1, 128.5, 127.8, 123.2,

117.2, 111.6, 55.9, 36.1, 31.2, 25.5. LC-MS (ESI) *m/z* found: 322 $[M+H]^+$; retention time: 3.50 minutes. HRMS-ESI $[M+H]^+$ calculated for $C_{19}H_{20}N_3O_2$: 322.1562, found: 322.1562.

2-(4-methoxy-3-(pyridin-3-yl)phenyl)-5-((tetrahydro-2H-pyran-4-yl)methyl)-1,3,4-oxadiazole (**134**)

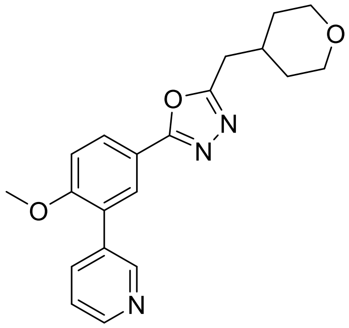

Oxadiazole **129** (100 mg, 0.28 mmol) and pyridin-3-ylboronic acid (45 mg, 0.37 mmol) were charged to a microwave vial after which DME (3 mL) and 1M Na_2CO_3 (1.0 mL, 1.0 mmol) were added. The mixture was degassed with N_2 for 5 m after which $Pd(dppf)Cl_2$ (12 mg, 0.01 mmol) was added. The reaction was heated in the microwave for 1 h at 120°C. The reaction mixture was diluted with EtOAc (30 mL) and filtered over Celite. The residue was washed with saturated $NaHCO_3$ (2x 20 mL) and brine (20 mL). The organic phase was dried over Na_2SO_4, filtered and concentrated *in vacuo* to be further purified over SiO_2 using a gradient of 40% EtOAc in c-hexane towards 5% MeoH in EtOAc to yield 42 mg (0.12 mmol, 42%) of the title compound. ^1H NMR (600 MHz, Chloroform-d) δ 8.81 (s, 1H), 8.63 – 8.56 (m, 1H), 8.07 (dd, *J* = 8.6, 1.8 Hz, 1H), 7.97 (d, *J* = 1.8 Hz, 1H), 7.90 (d, *J* = 7.7 Hz, 1H), 7.42 – 7.36 (m, 1H), 7.11 (d, *J* = 8.7 Hz, 1H), 3.98 (dd, *J* = 11.4, 3.6 Hz, 2H), 3.91 (s, 3H), 3.46 – 3.38 (m, 2H), 2.87 (d, *J* = 7.1 Hz, 2H), 2.19 – 2.12 (m, 2H), 1.72 (d, *J* = 12.9 Hz, 2H), 1.47 (qd, *J* = 12.4, 4.4 Hz, 2H). ^{13}C NMR (150 MHz, Chloroform-d) δ 165.1, 164.4, 159.1, 149.9, 148.2, 137.1, 133.2, 129.2, 128.6, 127.7, 123.2, 117.0, 111.6, 67.6, 55.9, 33.6, 32.7, 32.5. LC-MS (ESI) *m/z* found: 352 $[M+H]^+$; retention time: 2.86 minutes. HRMS-ESI $[M+H]^+$ calculated for $C_{20}H_{22}N_3O_3$: 352.1656, found: 352.1653.

2-(4-methoxy-3-(pyridin-3-yl)phenyl)-5-phenyl-1,3,4-oxadiazole (**135**)

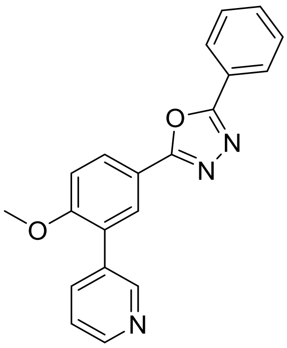

Oxadiazole **130** (74 mg, 0.30 mmol) and pyridin-3-ylboronic acid (48 mg, 0.39 mmol) were charged to a microwave vial after which DME (3 mL) and 1M Na_2CO_3 (1.0 mL, 1.0 mmol) were added. The mixture was degassed with N_2 for 5 m after which Pd(dppf)Cl_2 (12 mg, 0.02 mmol) was added. The reaction was heated in the microwave for 1 h at 120°C. The reaction mixture was diluted with EtOAc (30 mL) and filtered over Celite. The residue was washed with saturated $NaHCO_3$ (2x 20 mL) and brine (20 mL). The organic phase was dried over Na_2SO_4, filtered and concentrated *in vacuo* to be further purified over SiO_2 using a gradient of 40% EtOAc in c-hexane towards 100% EtOAc to yield 48 mg (0.15 mmol, 65%) of the title compound. ^{1}H NMR (500 MHz, Chloroform-d) δ 8.87 (s, 1H), 8.63 (d, *J* = 4.3 Hz, 1H), 8.19 (dd, *J* = 8.6, 2.2 Hz, 1H), 8.14 (dd, *J* = 7.9, 1.7 Hz, 2H), 8.09 (d, *J* = 2.2 Hz, 1H), 8.01 (dt, *J* = 8.0, 1.9 Hz, 1H), 7.59 – 7.50 (m, 3H), 7.48 (dd, *J* = 7.9, 4.9 Hz, 1H), 7.16 (d, *J* = 8.7 Hz, 1H), 3.93 (s, 3H). ^{13}C NMR (126 MHz, Chloroform-d) δ 164.4, 164.1, 159.2, 149.0, 147.1, 138.1, 133.7, 131.7, 129.3, 129.1, 129.0, 127.3, 126.9, 123.9, 123.6, 117.1, 111.8, 56.0. LC-MS (ESI) *m*/*z* found: 330 $[M+H]^+$; retention time: 3.63 minutes. HRMS-ESI $[M+H]^+$ calculated for $C_{17}H_{22}BrN_2O_4$: 330.1237, found: 330.1233.

2-(3-fluorophenyl)-5-(4-methoxy-3-(pyridin-3-yl)phenyl)-1,3,4-oxadiazole (**136**)

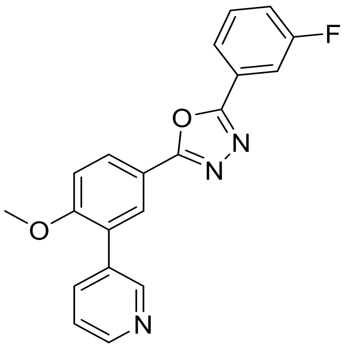

Oxadiazole **131** (100 mg, 0.29 mmol) and pyridin-3-ylboronic acid (46 mg, 0.37 mmol) were charged to a microwave vial after which DME (3 mL) and 1M Na_2CO_3 (1.0 mL, 1.0 mmol) were added. The mixture was degassed with N_2 for 5 m after which Pd(dppf)Cl_2 (12 mg, 0.02 mmol) was added. The reaction was heated in the microwave for 1 h at 120°C. The reaction mixture was diluted with EtOAc (30 mL) and filtered over Celite. The residue was washed with saturated $NaHCO_3$ (2x 20 mL) and brine (20 mL). The organic phase was dried over Na_2SO_4, filtered and concentrated *in vacuo* to be further purified over SiO_2 using a gradient of 40% EtOAc in c-hexane towards 100% EtOAc to yield 32 mg (0.09 mmol, 32%) of the title compound. ^{1}H NMR (600 MHz, Chloroform-d) δ 8.78 (d, *J* = 1.7 Hz, 1H), 8.61 (dd, *J* = 4.8, 1.4 Hz, 1H), 8.22 (dd, *J* = 8.6, 2.2 Hz, 1H), 8.13 (d, *J* = 2.2 Hz, 1H), 8.04 – 7.98 (m, 3H), 7.68 (td, *J* = 8.3, 6.0 Hz, 1H), 7.55 – 7.49 (m, 2H), 7.42 (d, *J* = 8.7 Hz, 1H), 3.92 (s, 3H). ^{13}C NMR (151 MHz, Chloroform-d) δ 164.5, 163.3 (d, *J* = 3 Hz), 162.8 (d, *J* = 244 Hz), 159.7, 150.0, 148.8, 137.5, 133.1, 132.2 (d, *J* = 8 Hz), 129.4 (d, *J* = 22 Hz), 127.8, 126.3, 125.9 (d, 9.0 Hz), 123.9, 123.4 (d, 2.8 Hz), 119.4 (d, *J* = 20 Hz), 116.4, 114.0 (d, *J* = 24 Hz), 113.2, 56.7. LC-MS (ESI) *m/z* found: 348 $[M+H]^+$; retention time: 3.80 minutes. HRMS-ESI $[M+H]^+$ calculated for $C_{20}H_{15}FN_3O_2$: 348.1143, found: 348.1146.

3-bromo-4-methoxybenzamide (**137**)

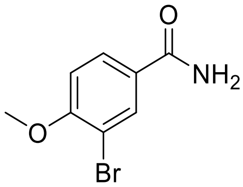

Benzoic acid **75** (6,3 g, 27.3 mmol) was suspended in DCM (50 mL) and oxalyl chloride (2.63 ml, 30.0 mmol) and a few drops of DMF were added. The mixture was stirred for 18 h after which volatiles were evaporated and the solids were redissolved in DCM (50 mL). Subsequently ammonium hydroxide (30% solution in water)(273 mmol, 31.9 g) was added and the mixture was stirred for 30 minutes. Solids were filtered off, washed with water and dried *in vacuo* to yield 4.7 g (20.4 mmol, 75%) of the title compound as a white solid.^{1}H NMR (300 MHz, DMSO-*d*6) δ 8.10 (d, *J* = 2.2 Hz, 1H), 7.95 (s, 1H), 7.90 (dd, *J* = 8.6, 2.2 Hz, 1H), 7.33 (s, 1H), 7.17 (d, *J* = 8.6 Hz, 1H), 3.90 (s, 3H). LC-MS (ESI) *m*/*z* found: 230 $[M+H]^+$; retention time: 3.03 minutes. HRMS-ESI $[M+H]^+$ calculated for $C_8H_9BrNO_2$: 229.9811, found: 229.9804.

3-bromo-4-methoxybenzothioamide (**138**)

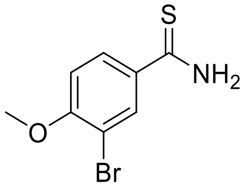

Benzamide **137** (2.5 g, 10.9 mmol) was suspended in toluene (50 mL) and Lawessons Reagent (2.64 g, 6.52 mmol) was added. The mixture was refluxed for 18 h after which the mixture was cooled down to rt, diluted with EtOAc (50 mL) and washed with water (2x 100 mL) and brine (100 mL). The resulting crude was purified over SiO_2 using a gradient of 30% EtOAc in heptane towards 100% EtOAc to yield 350 mg (1.4 mmol, 13%) of the title compound as a brown-orange solid. LC-MS (ESI) *m*/*z* found: 246 $[M+H]^+$; retention time: 3.70 minutes.

2-(3-bromo-4-methoxyphenyl)-4-cyclopropylthiazole (**139**)

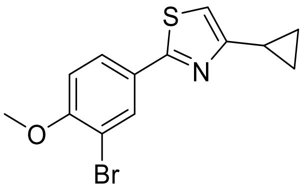

Thioamide **138** (100 mg, 0.41 mmol) was added to 2-Propanol (2 mL) followed by 2-bromo-1-cyclopropylethanone (66 mg, 0.40 mmol) and the mixture was stirred for 2 h at rt, followed by 2 h at reflux. The mixture was quenched with water (20 mL) and extracted with EtOAc (25 mL). The organic layer was washed with brine and dried over Na_2SO_4 after which volatiles were evaporated. The resulting solids were used in the next step without further purification. ^{1}H NMR (300 MHz, Chloroform-d) δ 8.12 (d, J = 2.0 Hz, 1H), 7.86 – 7.74 (m, 1H), 6.90 (d, J = 8.6 Hz, 1H), 6.77 (s, 1H), 3.93 (s, 3H), 1.27 (t, J = 7.2 Hz, 1H), 0.98 – 0.91 (m, 4H). LC-MS (ESI) *m/z* found: 310 $[M+H]^+$; retention time: 5.61 minutes.

2-(3-bromo-4-methoxyphenyl)-4-phenylthiazole (**140**)

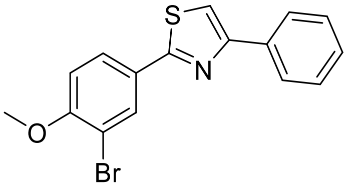

Thioamide **138** (100 mg, 0.41 mmol) was added to 2-propanol (2 mL) followed by 2-bromo-1-phenylethanone (81 mg, 0.40 mmol) and the mixture was stirred for 2 hours at rt, followed by 2 h at reflux. The mixture was quenched with water (20 mL) and extracted with EtOAc (25 mL). The organic layer was washed with brine and dried over Na_2SO_4 after which volatiles were evaporated. The resulting solids were used in the next step without further purification. ^{1}H NMR (300 MHz, Chloroform-d) δ 8.27 (d, J = 2.2 Hz, 1H), 8.03 – 7.89 (m, 3H), 7.50 – 7.40 (m, 3H), 7.39 – 7.31 (m, 1H), 6.96 (d, J = 8.6 Hz, 1H), 3.96 (s, 3H). LC-MS (ESI) *m/z* found: 348 $[M+H]^+$; retention time: 5.90 minutes.

4-cyclopropyl-2-(4-methoxy-3-(pyridin-3-yl)phenyl)thiazole (**141**)

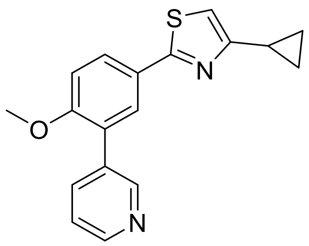

Thiazole **139** (126 mg, 0.41 mmol) and pyridin-3-ylboronic acid (65 mg, 0.53 mmol) were charged to a microwave vial after which DME (3 mL) and 1M Na_2CO_3 (1.2 mL, 1.2 mmol) were added. The mixture was degassed with N_2 for 5 m after which Pd(dppf)Cl_2 (17 mg, 0.02 mmol) was added. The reaction was heated in the microwave for 1 h at 120°C. The reaction mixture was diluted with EtOAc (30 mL) and filtered over Celite. The residue was washed with saturated $NaHCO_3$ (2x 20 mL) and brine (20 mL). The organic phase was dried over Na_2SO_4, filtered and concentrated *in vacuo* to be further purified over SiO_2 using a gradient of 40% EtOAc in c-hexane towards 100% EtOAc to yield 44 mg (0.14 mmol, 35% over two steps) of the title compound. ^{1}H NMR (600 MHz, Chloroform-d) δ 8.80 (s, 1H), 8.57 (d, *J* = 4.1 Hz, 1H), 7.92 – 7.84 (m, 3H), 7.34 (dd, *J* = 4.9, 2.7 Hz, 1H), 7.00 (d, *J* = 8.6 Hz, 1H), 6.76 (s, 1H), 3.85 (s, 3H), 2.06 (p, 1H), 0.94 (d, *J* = 6.7 Hz, 4H). ^{13}C NMR (150 MHz, Chloroform-d) δ 166.64, 159.81, 157.80, 150.29, 148.25, 136.85, 133.60, 128.80, 127.79, 127.50, 122.97, 111.37, 110.01, 55.77, 12.34, 8.06. LC-MS (ESI) *m/z* found: 309 $[M+H]^+$; retention time: 4.06 minutes. HRMS-ESI $[M+H]^+$ calculated for $C_{17}H_{22}BrN_2O_4$: 309.1061, found: 309.1056.

2-(4-methoxy-3-(pyridin-3-yl)phenyl)-4-phenylthiazole (**142**)

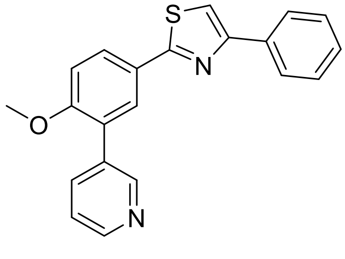

Thiazole **140** (141 mg, 0.41 mmol) and pyridin-3-ylboronic acid (65 mg, 0.53 mmol) were charged to a microwave vial after which DME (3 mL) and 1M Na_2CO_3

(1.2 mL, 1.2 mmol) were added. The mixture was degassed with N_2 for 5 m after which Pd(dppf)Cl_2 (17 mg, 0.02 mmol) was added. The reaction was heated in the microwave for 1 h at 120°C. The reaction mixture was diluted with EtOAc (30 mL) and filtered over Celite. The residue was washed with saturated $NaHCO_3$ (2x 20 mL) and brine (20 mL). The organic phase was dried over Na_2SO_4, filtered and concentrated *in vacuo* to be further purified over SiO_2 using a gradient of 40% EtOAc in c-hexane towards 100% EtOAc to yield 62 mg (0.18 mmol, 44% over two steps) of the title compound. ^{1}H NMR (600 MHz, Chloroform-d) δ 8.86 (s, 1H), 8.62 (s, 1H), 8.05 – 8.01 (m, 2H), 8.01 – 7.97 (m, 2H), 7.91 (dt, *J* = 7.9, 1.9 Hz, 1H), 7.47 – 7.42 (m, 3H), 7.40 – 7.33 (m, 2H), 7.07 (d, *J* = 8.2 Hz, 1H), 3.89 (s, 3H). ^{13}C NMR (150 MHz, Chloroform-d) δ 167.2, 158.1, 156.2, 150.3, 148.3, 136.9, 134.5, 133.6, 129.0, 128.7, 128.2, 128.0, 127.7, 127.1, 123.1, 112.0, 111.5, 55.8. LC-MS (ESI) *m/z* found: 345 $[M+H]^+$; retention time: 4.68 minutes. HRMS-ESI $[M+H]^+$ calculated for $C_{21}H_{17}N_2OS$: 345.1056, found: 345.1063.
